# Dietary Analyses and Personalized Advice in Patients with Inflammatory Bowel Disease: The DAPA Study

**DOI:** 10.3390/nu18162626

**Published:** 2026-08-11

**Authors:** Marjo J. E. Campmans-Kuijpers, Gerard Dijkstra

**Affiliations:** 1 Department of Gastroenterology & Hepatology, University Medical Centre Groningen, 9713 GZ Groningen, The Netherlands; 2 Faculty of Medical Sciences, University of Groningen, 9712 CP Groningen, The Netherlands

**Keywords:** nutrition, eHealth, inflammatory bowel disease, food-related quality of life, diet quality, dietary assessment

## Abstract

**Background/Objectives**: Patients with inflammatory bowel disease (IBD) frequently experience food-related symptoms and express a strong need for dietary guidance, yet evidence-based recommendations remain limited. This often leads to self-imposed dietary restrictions, potentially compromising nutritional intake and food-related quality of life (Fr-QoL). The primary aim of this study was to evaluate changes in Fr-QoL, following personalized feedback on an IBD-specific food frequency questionnaire and access to an online nutritional knowledge portal over a 6-month period. Secondary exploratory outcomes included changes in dietary intake and diet quality. **Methods**: In this nationwide pre–post study, 108 patients with IBD completed baseline and 6-month assessments. Dietary intake was measured using the validated GINQ-FFQ and Fr-QoL using a 29-item questionnaire. Nutrient intake was compared with EFSA reference values. Participants received automated personalized feedback and had access to educational tools and dietitian resources. Changes over time were analyzed using paired statistical tests. **Results:** A significant improvement in Fr-QoL was observed (mean difference 8.1 points, 95% CI 5.5–10.7; *p* < 0.001; Cohen’s d = 0.39). Baseline intake was characterized by high fat and low fiber consumption. After 6 months, total energy intake decreased (*p* < 0.001), along with reductions in protein, fat, carbohydrates, fiber, and several micronutrients, including iron, folate, vitamin B6, vitamin C, potassium, magnesium, and zinc. Diet quality did not improve, and adherence to EFSA guidelines remained suboptimal. The digital tools were positively evaluated. **Conclusions:** In this single-arm pre–post study, Fr-QoL scores were higher at follow-up than at baseline among participants who completed both assessments, whereas dietary intake and overall diet quality did not improve. The findings support the feasibility and acceptability of the developed digital tools for dietary assessment and nutrition education, although their effectiveness requires further evaluation in controlled studies. Additional strategies, including structured dietitian-led support combined with digital resources, may be required to optimize nutritional outcomes in patients with IBD.

## 1. Introduction

Patients with inflammatory bowel disease (IBD) frequently report food-related symptoms and express a strong need for dietary guidance to prevent disease flares. However, gastroenterologists and dietitians often lack clear, evidence-based dietary recommendations. Consequently, many patients independently experiment with dietary restrictions, which are often associated with inadequate nutritional intake and nutrient deficiencies [1]. Educating patients regarding their IBD, including their nutritional status, is important for their empowerment and disease management [2].

This lack of clarity regarding appropriate food choices has been associated with nutritional inadequacies and lower food-related quality of life (Fr-QoL) [3]. Uncertainty about what can be safely consumed often results in dietary anxiety and reduced participation in social eating situations. Therefore, it is crucial that patients with IBD receive clear, evidence-based dietary guidance delivered by dietitians with specific expertise in IBD.

Nevertheless, evidence increasingly suggests that nutrition plays an important role in the management of IBD. In pediatric patients with Crohn’s disease, exclusive enteral nutrition (EEN) is recommended for the induction of remission, since pharmacological treatments such as corticosteroids are associated with impaired linear growth [4,5]. More recently, studies have demonstrated that in both pediatric and adult patients with refractory IBD, enteral nutrition combined with an exclusion diet, the so-called Crohn’s disease Exclusion Diet (CDED), can induce remission, even after failure of advanced and costly medical therapies [6,7]. In addition, an increasing body of literature suggests that anti-inflammatory dietary patterns may positively influence disease activity and patient outcomes [8]. Given the high prevalence of overlapping irritable bowel syndrome (IBS)-like symptoms in patients with IBD, it is clinically relevant to assess whether short-term implementation of a low-FODMAP diet may provide symptomatic benefit in selected patients [9]. Dietary guidance is regarded as important by 80% to 89% of patients with IBD, yet only 8% to 16% reported receiving adequate dietary information [10]. However, nutritional information related to diet and inflammatory bowel disease (IBD) is limited in accessibility for patients. In addition, patients often lack practical guidance on how to optimize key aspects of their dietary intake, such as fiber and calcium consumption. To address these gaps, we developed evidence-based nutritional knowledge into a dedicated knowledge tool that provides clear answers to frequently asked questions (FAQs). This tool was designed to support patients in evaluating their dietary quality and to facilitate consistent, evidence-based nutritional counseling by dietitians and IBD nurses.

For both patients with inflammatory bowel disease (IBD) and healthcare professionals involved in their care, it is essential to have insight into patients’ dietary intake. Therefore, patients completed the GINQ-FFQ, a food frequency questionnaire specifically developed for individuals with IBD [11,12]. This questionnaire was automated to enable patients to independently review and understand their dietary intake and to receive structured feedback on how to improve nutritional quality.

Importantly, all dietary interventions in IBD should be delivered under the supervision of trained dietitians to ensure nutritional adequacy, prevent deficiencies, and support long-term disease control. Because gastroenterology-focused dietitians have a broad scope of practice, there are only a limited number of well-trained IBD-specialized dietitians in the Netherlands. To address this gap, we established a nationwide educational program to train IBD-specialized dietitians. These dietitians were registered in a special list that was made available to all IBD healthcare professionals.

To reduce unnecessary outpatient visits and enhance disease monitoring, patients were followed using the validated eHealth platform MyIBDCoach, which has been shown to safely monitor disease activity, relevant lifestyle factors, and early signs of disease flares [13]. To facilitate personalized nutritional management, we integrated a newly developed IBD-specific dietary assessment tool, the food-related quality of life questionnaire [14] linked to individualized dietary advice, and a registry of IBD-trained dietitians into the MyIBDCoach application. Patients themselves frequently express a strong interest in using nutrition to influence the course of their disease. Providing patients with structured feedback and access to relevant e-learning modules through this platform was expected to facilitate greater insight into dietary habits and Fr-QoL.

To address these gaps, we developed a digital nutrition support program consisting of personalized dietary feedback, an IBD-specific nutritional knowledge tool, and access to a directory of IBD-specialized dietitians through the MyIBDCoach platform.

The present study was designed as an implementation study to evaluate the uptake, usability, and preliminary effectiveness of this digital nutrition support program in patients with IBD.

The primary aim of this study was to evaluate changes in Fr-QoL after six months of access to a digital nutrition support program consisting of personalized dietary feedback, an IBD-specific nutritional knowledge tool, and a directory of IBD-specialized dietitians.

Secondary exploratory outcomes included changes in dietary intake and diet quality. Additional implementation objectives were to evaluate the use and appreciation of these digital tools, facilitate the establishment of a nationwide network of IBD-specialized dietitians, and improve access to evidence-based nutritional information for patients with IBD.

## 2. Materials and Methods

### 2.1. Study Design and Participants

This nationwide pre–post implementation study evaluated the uptake, usability, and preliminary effectiveness of a digital nutrition support program in patients with IBD. This single-arm intervention study aimed to recruit at least 100 individuals with IBD. They were recruited between August 2024 and July 2025. Participants were recruited either through their treating IBD healthcare professionals or via national online recruitment campaigns by Crohn & Colitis NL, the Dutch patient organization. Eligible participants included individuals with IBD who already used an IBD-related mobile application (e.g., MyIBDCoach or IBDream), as well as those who expressed interest in assessing and improving their dietary intake. Participation was possible after providing written informed consent through an online consent form. All participants had to be willing to complete two questionnaires at baseline: (1) the food-related quality of life questionnaire (Fr-QoL), and (2) an IBD-specific food frequency questionnaire (GINQ-FFQ).

Following completion of the baseline questionnaires, participants received personalized feedback on both questionnaires and were granted access to an online nutritional knowledge tool (Supplementary File S1). Six months later, participants were invited to complete the same questionnaires again, together with a set of evaluation questions. Upon completion, updated personalized feedback was provided.

The intervention consisted of three components: (1) personalized dietary feedback generated through the GINQ-FFQ, (2) access to an online nutritional knowledge tool (“Food and IBD”), and (3) access to a directory of IBD-specialized dietitians through the MyIBDCoach platform.

The primary outcome of this implementation study was change in food-related quality of life (Fr-QoL) between baseline and 6-month follow-up. Secondary exploratory outcomes included changes in dietary intake and diet quality. Additional implementation outcomes included evaluation and use of the digital tools, as well as the establishment of a nationwide network of IBD-specialized dietitians.

The study was performed according to the Declaration of Helsinki and approved by the medical ethics committee of the University Medical Center Groningen (UMCG) (METc 2023/301, date of approval 16 June 2023).

### 2.2. Dietary Intake by IBD Food Frequency Questionnaire (GINQ-FFQ) and Feedback

Dietary intake was assessed by the GINQ-FFQ, a validated IBD food frequency questionnaire [11]. Patient intake results were evaluated in relation to individual nutritional requirements based on European Food Safety Authority (EFSA) reference values, taking into account sex, age, body weight, height, physical activity level, and, where applicable, pregnancy or lactation status [15]. Compliance with EFSA dietary reference values was defined as achieving the Population Reference Intake (PRI), or the Adequate Intake (AI) when no PRI had been established. For macronutrients, compliance was defined as intake within the EFSA reference intake range. Upon completion of the GINQ-FFQ at baseline and after the intervention, feedback was provided for total energy intake, macronutrients, and most vitamins and minerals, presented visually as an intake level on a continuous slider relative to individual requirements. For each nutrient, participants received a visual slider indicating whether intake was below, within, or above the individual EFSA reference range. Personalized advice was generated automatically by comparing individual nutrient intakes obtained from the GINQ-FFQ with the corresponding EFSA dietary reference values. Participants also received a brief explanatory text describing the physiological importance of each nutrient, together with practical food-based guidance on how to increase or decrease intake when indicated. Representative examples of the personalized feedback report are provided in Supplementary File S2. Up to 3 reminders were sent by email when participants did not complete the questionnaires.

In addition to quantitative feedback, participants received a brief explanatory text describing the physiological importance of each nutrient, together with practical guidance on how to increase or decrease intake when indicated. The FFQ was fully automated, quantitatively linked to EFSA reference values, and designed to deliver standardized, personalized feedback. The content and usability of the tool were evaluated by patients to ensure clarity of information and compliance with regulatory requirements, confirming that it does not fall under the Medical Device Regulation. An example was provided online (Supplementary File S2).

### 2.3. Food-Related Quality of Life (Fr-QoL)

Fr-QoL was assessed using the validated 29-item Fr-QoL questionnaire, specifically developed for patients with IBD. Each item was scored on a 5-point Likert scale ranging from 1 (strongly disagree) to 5 (strongly agree) [14]. The total Fr-QoL score was calculated by summing all item scores, resulting in a total score ranging from 29 to 145, with higher scores indicating better food-related quality of life. Although lower scores generally reflect greater food-related concerns and restrictions, no universally accepted cut-off values or minimally clinically important difference have been established for this instrument. Therefore, Fr-QoL scores were analyzed and interpreted as a continuous measure. Lower Fr-QoL scores were associated with active disease, greater dietary restriction, increased food-related anxiety, and reduced overall quality of life. Participants received feedback on their Fr-QoL score.

### 2.4. Nutritional Knowledge Tool: “Nutrition in IBD”

The nutritional knowledge tool provided people with IBD access to online up-to-date, evidence-based dietary information presented in a frequently asked questions (FAQ) format. Topics included an overview of IBD, the intestinal microbiome, strategies to achieve adequate dietary fiber intake, and approaches to ensure sufficient protein consumption. The content of the knowledge tool was reviewed by a dietitian, a physician, and a layperson to ensure clinical accuracy and comprehensibility. An overview of the topics covered in the knowledge tool is provided in Table A1. The complete Dutch-language version of the knowledge tool is available as Supplementary File S1.

### 2.5. Directory of IBD-Trained Dietitians

Registered dietitians were eligible to participate in the training program. The program was developed and delivered by two IBD-specialized dietitians, IBD nurses, and a gastroenterologist, and consisted of lectures and interactive workshops with a total study load of approximately 12 h. Topics included the pathophysiology of IBD, evidence-based dietary management of IBD, nutritional deficiencies, enteral nutrition, and dietary counseling strategies.

A total of 150 dietitians successfully completed the program and were included in a national directory of IBD-trained dietitians. This directory was accessible to healthcare professionals involved in IBD care, including gastroenterologists, IBD nurses, and nurse specialists, and was also available online for patients. This directory enabled patients and healthcare providers to identify an IBD-trained dietitian in their local area. Participants who required additional dietary support could contact a dietitian listed in the directory. Consultations with dietitians are covered by Dutch health insurance under the conditions of the individual insurance policy.

### 2.6. Dietary Quality Scores

Diet quality was evaluated using complementary nutrient-based approaches. The Dietary Inflammatory Index (DII) was computed to quantify the inflammatory potential of the diet, based on standardized and energy-adjusted intakes of individual nutrients and bioactive components, which were scored according to their pro- or anti-inflammatory properties derived from the literature, with higher scores indicating a more pro-inflammatory profile [16]. Adherence to a Mediterranean dietary pattern was assessed using a nutrient-adapted alternate Mediterranean diet score (aMED), in which established component criteria were operationalized using nutrient proxies (e.g., dietary fiber as a marker of plant-based foods, and the monounsaturated-to-saturated fatty acid ratio as an indicator of fat quality), with higher scores reflecting greater adherence [17]. In addition, dietary patterns were identified using principal component analysis (PCA), an exploratory multivariate dimension-reduction technique applied to correlated food group intake variables [18]. Orthogonal components were extracted based on the covariance structure of the data, and factor loadings were used to characterize the contribution of individual food groups to each component. Individual factor scores were subsequently calculated using the regression method and were used to quantify adherence to each dietary pattern.

### 2.7. Statistical Analysis

Analyses were performed according to the predefined outcome hierarchy. Fr-QoL was the primary outcome. Dietary intake and diet quality measures were analyzed as secondary exploratory outcomes. Use and appreciation of the digital tools were analyzed descriptively as implementation outcomes.

Baseline characteristics were presented as mean ± standard deviation (SD) for normally distributed continuous variables, median (interquartile range, IQR) for non-normally distributed variables, and number (percentage) for categorical variables. To assess potential attrition bias, baseline characteristics of participants who completed follow-up were compared with those of non-completers using independent-samples t-tests, Mann–Whitney U tests, χ^2^ tests, or Fisher’s exact tests, as appropriate.

Analyses of changes between baseline and follow-up were performed using complete-case data. Changes in Fr-QoL scores and dietary intake between baseline and follow-up were analyzed using paired-samples t tests or Wilcoxon signed-rank tests, as appropriate. For physical activity, ANOVA was used to compare groups. One-sample t tests were used to assess whether mean nutrient intakes differed significantly from EFSA reference values. As a sensitivity analysis, a linear mixed-effects model with participant as a random effect and time (baseline versus follow-up) as a fixed effect was performed using all available Fr-QoL observations.

To explore factors associated with follow-up Fr-QoL, a multivariable linear regression analysis was performed. Follow-up Fr-QoL was included as the dependent variable, while consultation with an IBD dietitian (diet use), frequency of knowledge tool use (KT use), and perceived usefulness of the knowledge tool for adapting dietary behavior (KT weight) were included as independent variables.

Dietary patterns were identified using principal component analysis (PCA) applied to food group intake variables. The suitability of the data for PCA was assessed using the Kaiser–Meyer–Olkin (KMO) measure of sampling adequacy and Bartlett’s test of sphericity. Components were initially extracted based on eigenvalues >1. The final number of components retained was determined using inspection of the scree plot and assessment of component interpretability. Varimax rotation was applied to improve interpretability of the component structure, and food groups with factor loadings ≥ |0.30| were considered important contributors to a dietary pattern. Individual factor scores were calculated using the regression method.

As this study was designed as an implementation study and no formal sample size calculation was performed, all inferential analyses should be interpreted as exploratory and hypothesis-generating rather than confirmatory. Given the exploratory nature of the dietary and nutritional analyses, no adjustment for multiple comparisons was applied. Therefore, findings for secondary and exploratory outcomes should be interpreted with caution. A two-sided *p* value < 0.05 was considered statistically significant. IBM SPSS Statistics 30.0 (IBM Corp., Armonk, NY, USA).was used. Questionnaire data were collected and managed using REDCap electronic data capture tools, version 16.0.43 (Vanderbilt University, Nashville, TN, USA), hosted at University Medical Centre Groningen.

## 3. Results

### 3.1. Study Population

#### 3.1.1. Baseline Characteristics

**Figure 1:**
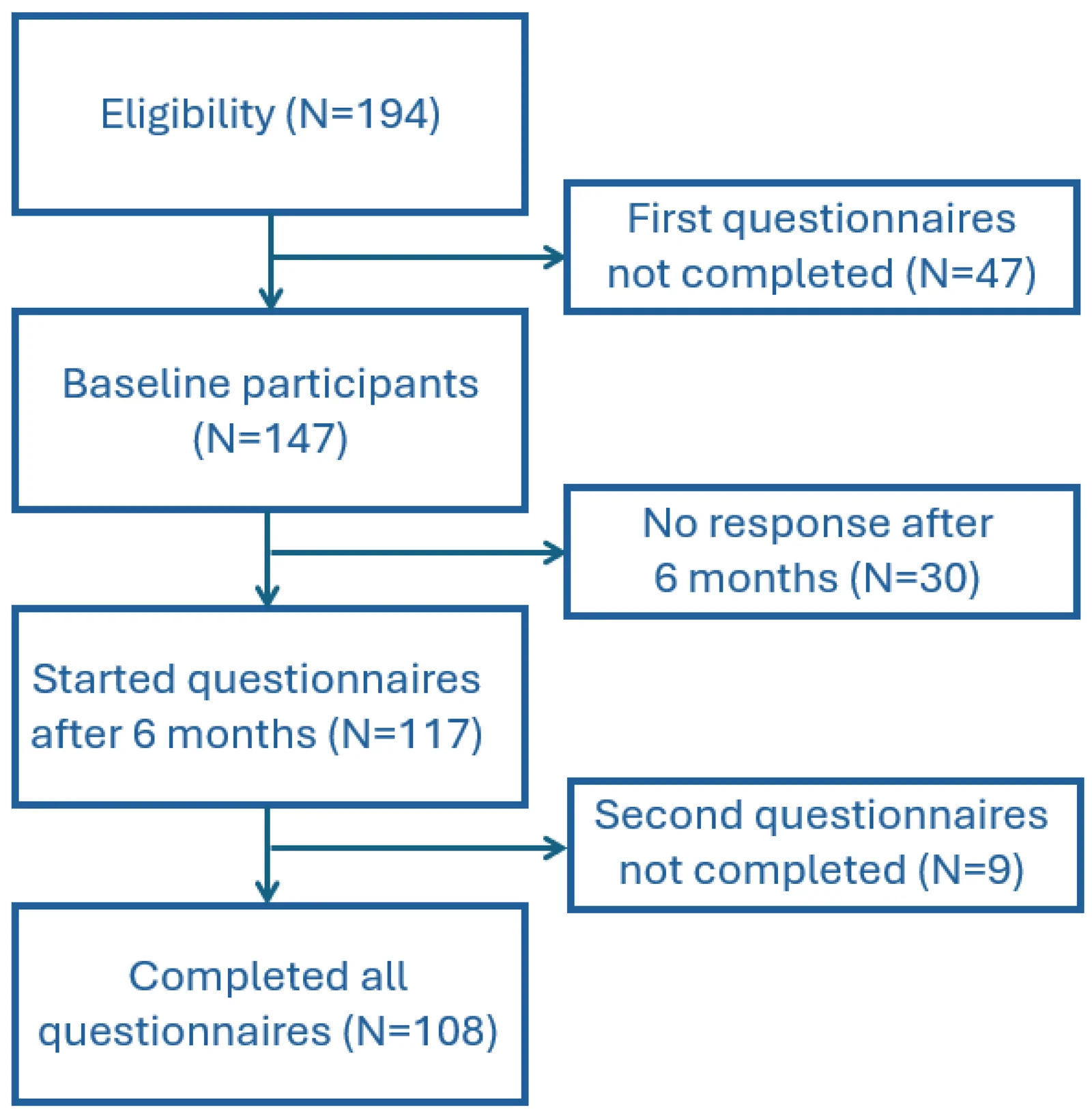
Flowchart of inclusion process.

A total of 194 individuals expressed interest in participating in the study, of whom 147 completed the baseline questionnaires. After 6 months, 117 participants started the questionnaires, of whom 108 participants filled these out completely (Figure 1).

Among the 108 participants who completed both assessments, 72 (67%) were female; 53.2% had Crohn’s disease (CD), 42.2% ulcerative colitis (UC), and 3.7% IBD unclassified (IBDU). Mean age was 44.5 years (SD 15.8), and the mean body mass index (BMI) was 25.0 kg/m^2^ (SD 4.8) (Table 1).

#### 3.1.2. Attrition Bias Analysis

To assess attrition bias, baseline characteristics of participants who completed both FFQs (*n* = 108) were compared with those who did not complete the 6-month assessment (*n* = 39) (Table A2). Completers were older (44.5 ± 15.8 vs. 38.8 ± 12.9 years, *p* = 0.049) and had higher baseline Fr-QoL scores (91.5 ± 22.6 vs. 79.9 ± 21.7, mean difference 11.5 points, 95% CI 3.6–19.5, *p* = 0.005). No statistically significant differences were observed for sex, IBD subtype, disease duration, BMI, physical activity level, total energy intake, protein intake, fat intake, BMR, EI/BMR ratio, or prevalence of underreporting according to Goldberg cut-offs. Baseline intakes of potassium, magnesium and non-haem iron were higher among completers than non-completers. These findings suggest the presence of attrition bias, as participants who completed follow-up were older and had a better food-related quality of life at baseline than those who did not complete the study.

According to the Goldberg cut-offs, 28 of 108 completers (26%) and 9 of 39 non-completers (23%) were classified as under-reporters, indicating no evidence of differential dietary misreporting between groups. Baseline characteristics of completers and non-completers are shown in Table A2.

#### 3.1.3. Sensitivity Analysis

To evaluate the potential impact of missing follow-up data, a sensitivity analysis using a linear mixed-effects model was performed including all available Fr-QoL observations from the 147 participants who completed the baseline assessment. The model demonstrated a significant effect of time on Fr-QoL (F = 48.45, *p* < 0.001). Estimated mean Fr-QoL scores increased from 88.3 at baseline to 97.3 at follow-up, corresponding to a mean improvement of 9.0 points (95% CI 6.4–11.5). These findings were consistent with the complete-case analysis.

### 3.2. Patient-Level Outcomes

#### 3.2.1. Change in Fr-QoL

Among the 108 participants who completed both assessments, Fr-QoL scores increased from 91.5 *±* 22.6 at baseline to 99.7 *±* 22.5 after 6 months. The mean improvement was 8.1 points (95% CI 5.5 to 10.7; *p* < 0.001), corresponding to a small to moderate effect size (Cohen’s d = 0.39). To evaluate the potential impact of missing follow-up data, a sensitivity analysis using a linear mixed-effects model was performed, including all available Fr-QoL observations from the 147 participants who completed the baseline assessment. The model demonstrated a significant effect of time on Fr-QoL (F = 48.45, *p* < 0.001). Estimated mean Fr-QoL scores increased from 88.3 at baseline to 97.3 at follow-up, corresponding to a mean improvement of 9.0 points (95% CI 6.4–11.5). These findings were consistent with the complete-case analysis.

Intervention exposure varied across participants. Of the 108 participants who completed the evaluation questionnaire, 22 (20.4%) reported consulting an IBD dietitian during the study period. Regarding the knowledge tool, 15 participants (13.9%) reported using it multiple times, whereas 36 (33.3%) reported using it occasionally. Data on whether participants accessed the individualized feedback reports or consulted external online nutrition resources were not collected.

A multiple linear regression analysis was performed to examine the association between diet-related factors and follow-up Fr-QoL. The model included Diet use, KT weight, and KT use as independent variables and follow-up Fr-QoL as the dependent variable. The overall model explained 7.7% of the variance in follow-up Fr-QoL (R^2^ = 0.077) but did not reach statistical significance (F(3,96) = 2.67, *p* = 0.052) (Table 2).

Among the predictors, only diet use was significantly associated with follow-up Fr-QoL (B = −11.69, SE = 4.85, β = −0.238, *p* = 0.018). No significant associations were observed for KT weight (B = −3.50, SE = 4.22, β = −0.103, *p* = 0.409) or KT use (B = 0.71, SE = 2.60, β = 0.034, *p* = 0.786).

#### 3.2.2. Change in Nutritional Intake

Comparing the nutritional intakes of patients who completed the GINQ-FFQ at both time points, most nutrient intakes were lower after the intervention than at baseline. These analyses should be interpreted as exploratory, as no adjustment for multiple comparisons was applied. Total energy intake was significantly lower at follow-up, particularly among women (Table 3). Total fat intake, including omega-3 and omega-6 fatty acids, also decreased significantly. Carbohydrate intake was lower at follow-up, accompanied by lower starch intake. Fiber intake decreased from a mean of 26.3 (SD 11.0) to 23.3 (SD 12.0) g. Iron intake was also lower at follow-up, particularly non-haem iron intake. In addition, vitamin B2 and folic acid intakes were lower at follow-up.

#### 3.2.3. Compliance with EFSA Reference Values

At both timepoints, all patients consumed excessive amounts of fat, mainly due to a high intake of saturated fats, whereas almost all patients met the recommended intake for omega-3 fatty acids. Protein intake was compliant with EFSA guidelines in 55% of patients at baseline and 56% after 6 months. The proportion of patients meeting fiber recommendations decreased from 30% at baseline to 26% at follow-up. For an overview of compliance with the EFSA guidelines, see Table A3 and Table A4.

#### 3.2.4. Diet Quality Scores

Principal component analysis (PCA) was performed on food group intake variables. The data were considered suitable for PCA, with a Kaiser–Meyer–Olkin (KMO) measure of sampling adequacy of 0.636 and a significant Bartlett’s test of sphericity (χ^2^ = 812.994, df = 231, *p* < 0.001). PCA initially identified eight components with eigenvalues >1, explaining 65.6% of the total variance. However, inspection of the scree plot indicated a leveling-off after the fifth component, and components 6–8 were not readily interpretable as meaningful dietary patterns. Therefore, five dietary patterns were retained for further analyses. These five patterns explained 49.2% of the total variance after Varimax rotation. Food groups with factor loadings ≥ |0.30| were considered meaningful contributors to a dietary pattern (Table A5). The first pattern was labeled Snack and Convenience pattern, characterized by high intakes of savory snacks, sweets, bread, sauces, ready meals, fats, cakes, and potatoes. The second pattern, labeled Plant-based pattern, was characterized by high consumption of legumes, nuts and seeds, grains, vegetables, and fruit, and a lower intake of processed meats. The third pattern, labeled Animal protein pattern, was characterized by higher intakes of eggs, meat and poultry, and fish. The fourth pattern, labeled Traditional staple pattern, was characterized by higher consumption of potatoes, vegetables, fruit, and cold cuts, and lower intake of cakes and beverages. The fifth pattern, labeled health-conscious pattern, was characterized by higher intake of fats, nuts and seeds, vegetables, and fruit and lower consumption of alcohol, non-alcoholic beverages, and cheese (Table A6). In multivariable regression analyses adjusted for age, sex, and BMI, no statistically significant associations were observed between the identified dietary patterns and quality of life. However, a borderline positive association was observed for the Animal protein dietary pattern (β = 0.19, *p* = 0.065), suggesting a potential relationship that warrants further investigation.

The dietary inflammatory index (DII) at baseline was −0.414 and changed to −0.152 after six months. Thus, the DII did not differ significantly between baseline and follow-up (Table A7). Consistent with this, no significant associations were observed between DII and quality of life.

The mean aMED score remained unchanged from baseline to post-intervention (4.95 ± 1.30 vs. 5.00 ± 1.27; *p* = 0.791), indicating no significant change in Mediterranean diet adherence.

### 3.3. User Evaluation and Intervention Exposure

#### 3.3.1. Evaluation of the Food Frequency Questionnaire

**Figure A1:**
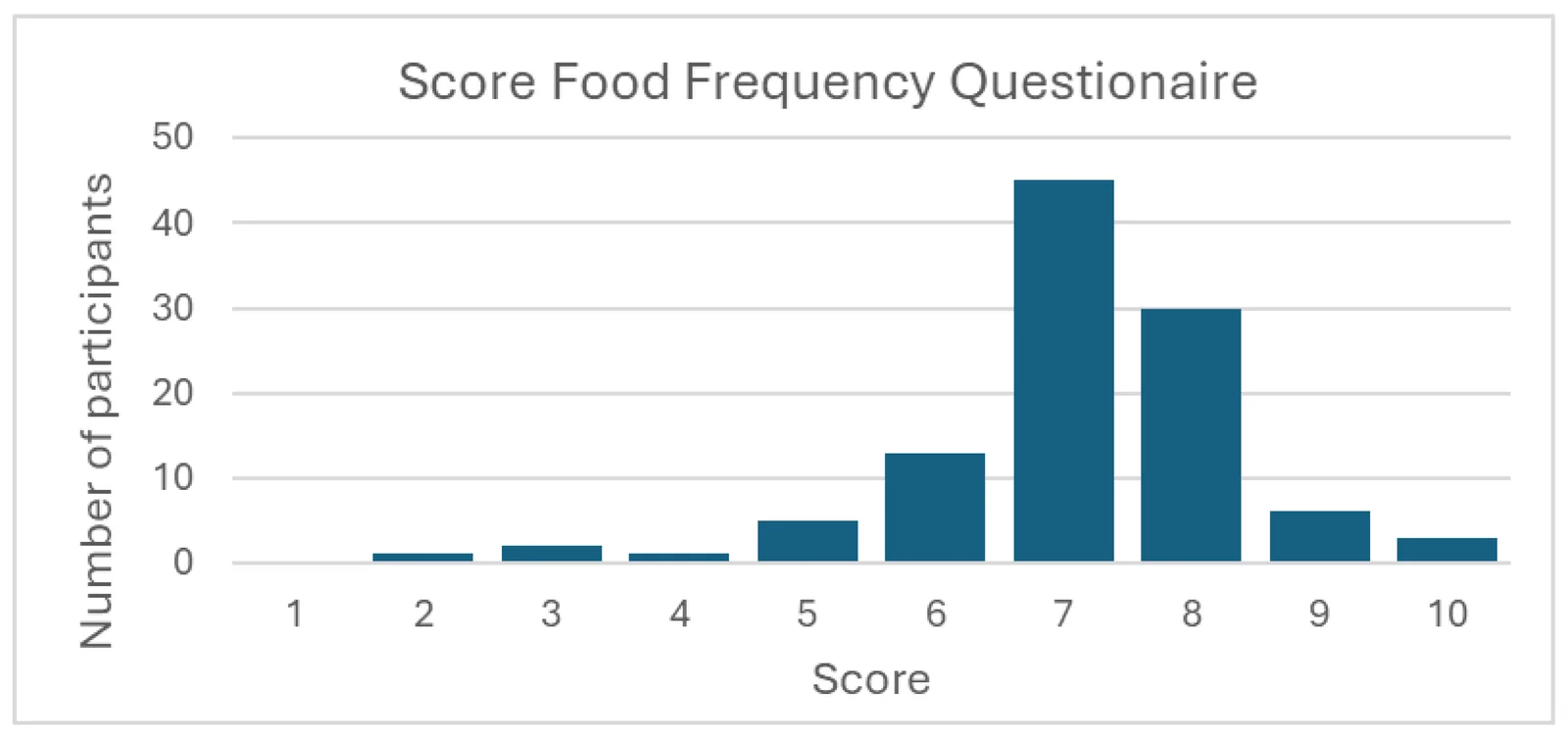
Score Feedback Food Frequency Questionnaire; 106 patients scored the GINQ-FFQ with a mean score of 7.11 ± 1.3.

Participants generally reported a positive evaluation of the GINQ-FFQ questionnaire. The majority rated the questionnaire as clear and easy to complete, although it was considered to contain (too) many questions. In total, 106 patients rated the GINQ-FFQ with a mean score of 7.11 (SD1.3) (Figure A1).

#### 3.3.2. Evaluation of the Knowledge Tool

**Figure A2:**
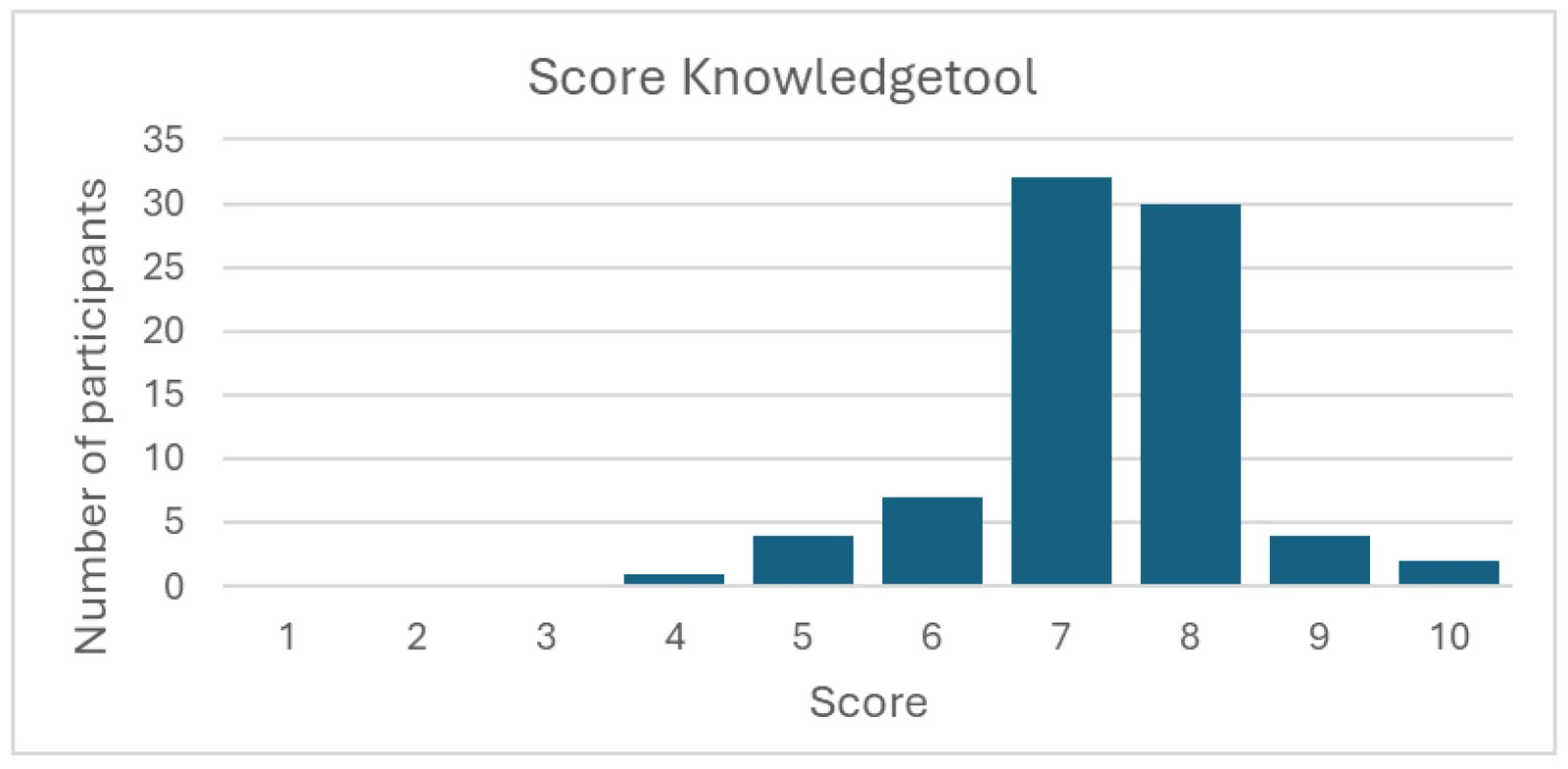
Score on the knowledge tool.

Eighty patients evaluated the knowledge tool, with a mean score of 7.2 (SD 1.3). Eighteen patients were unaware of the tool or did not recall receiving it, and 28 patients did not use it. (Figure A2).

Use of the IBD knowledge tool was assessed using a 5-point Likert scale. Of the respondents, none reported frequent or intensive use (score 1). Fifteen participants reported using the tool multiple times (score 2), 36 reported occasional use (score 3), 30 reported minimal use (score 4), and 20 reported not using the tool at all (score 5).

**Figure A3:**
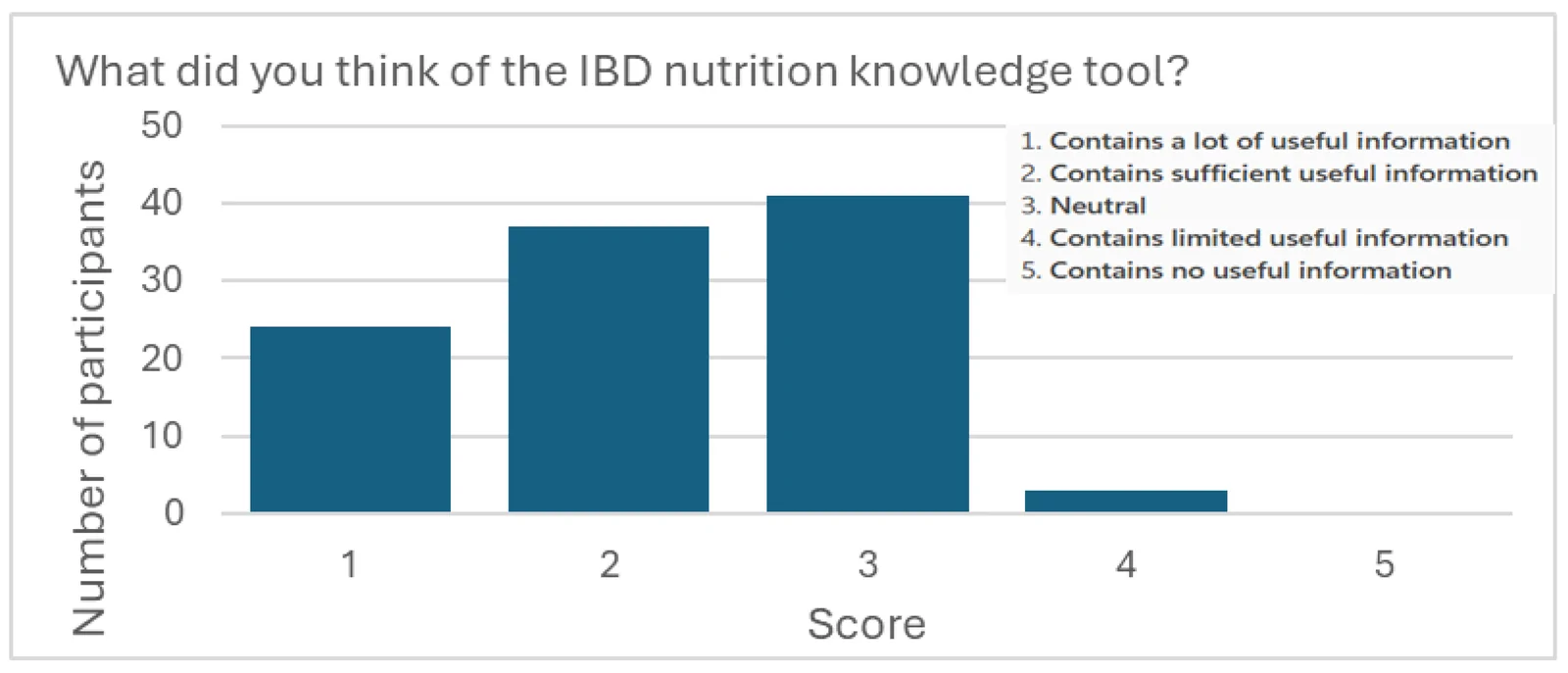
What did you think of the IBD nutrition knowledge tool?

Most users reported a neutral evaluation of the usefulness of the knowledge tool (*n* = 41, 39%). Thirty-seven participants (35%) indicated that the tool contained sufficient useful information, while 24 participants (23%) reported that it contained a lot of useful information. Only three participants reported limited usefulness, and none indicated that the tool contained no useful information. (Figure A3).

Among the 27 topics included in the knowledge tool, dietary fiber was accessed most frequently (48 views), followed by ensuring sufficient protein intake (40 views) and foods that may help prevent inflammation (37 views). An overview of usage across all topics is provided in Table 4.

**Figure A4:**
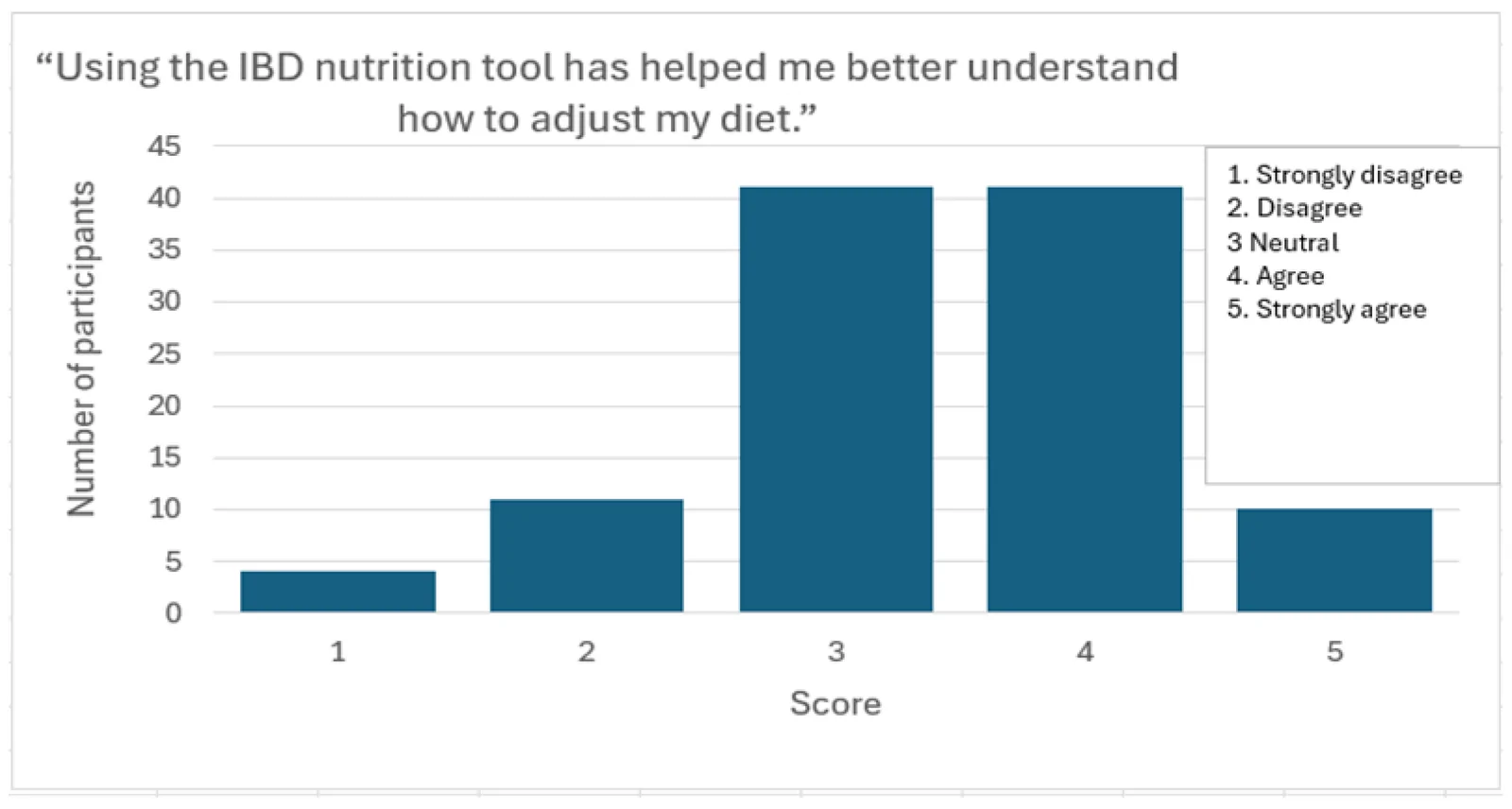
Using the IBD nutrition tool has helped me better understand how to adjust my diet.

Responses to the statement “Using the IBD nutrition tool has helped me better understand how to adjust my diet” are shown in Figure A4. Overall, participants reported predominantly neutral to positive experiences, suggesting that many users perceived the tool as helpful for increasing their dietary awareness, while only a few reported little added value.

#### 3.3.3. Overall Study Evaluation

**Figure A5:**
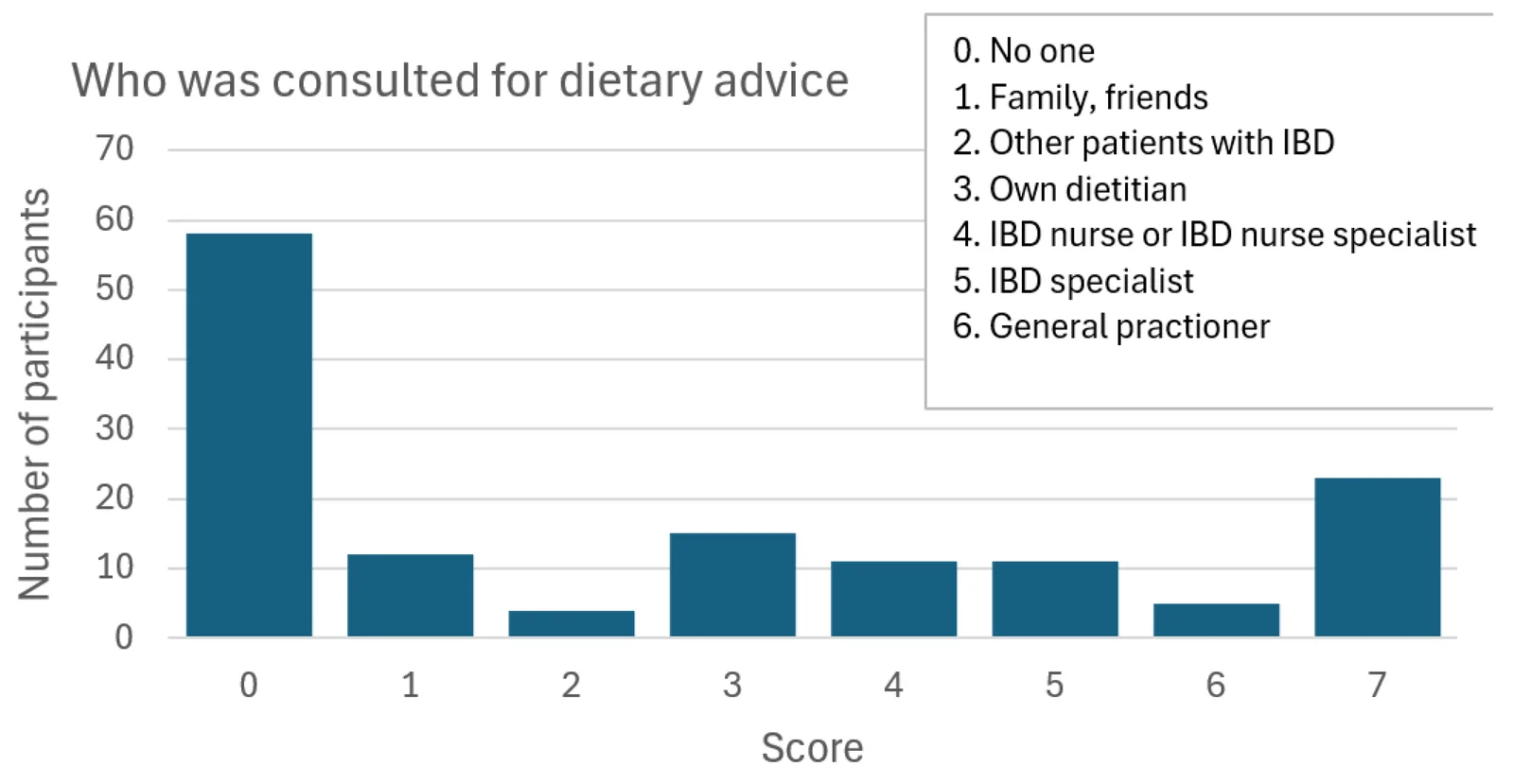
Who was consulted for dietary advice during the study?

For dietary advice, 22 participants (20.4%) consulted an IBD dietitian, 58 (53.7%) did not seek additional advice, and 23 (21.3%) consulted online sources. A complete overview is provided in Figure A5 in Appendix A.

Responses to the question, “Did this project contribute to better control of your diet in relation to IBD?”, were assessed using a 5-point Likert scale (1 = strongly disagree to 5 = strongly agree). In total, 104 participants completed this item. Most participants reported a neutral response (score 3; *n* = 51, 49%). Agreement with the statement was reported by 36 participants (35%; score 4) and strong agreement by 2 participants (2%; score 5). Disagreement was reported by 11 participants (11%; score 2), while 4 participants (4%; score 1) strongly disagreed. Participants were also invited to provide open-ended comments regarding their experiences with the project. Representative quotes illustrating the range of participant perspectives, including positive, neutral, and limited perceived impact, are presented in Table 5. These quotes are provided for illustrative purposes only and were not subjected to formal qualitative analysis.

## 4. Discussion

In this before-after intervention study, 108 patients with IBD completed all questionnaires. This implementation study resulted in several outputs, including the training of 150 IBD-specialized dietitians, the development of a digital IBD-specific food frequency questionnaire (GINQ-FFQ) providing personalized feedback on dietary intake, and the development of the “Food and IBD” knowledge tool. Both tools were positively evaluated by participants, with mean ratings of 7.1 (SD 1.3) and 7.2 (SD 1.3), respectively. Food-related quality of life scores were significantly higher at follow-up than at baseline, with Fr-QoL scores increasing from 91.5 (SD 22.6) to 99.7 (SD 22.5). However, no improvements were observed in dietary intake or diet quality. Instead, participants reported lower intakes of energy, fat, carbohydrates, and fiber. These changes were not accompanied by changes in BMI.

Educating patients about their IBD, including their nutritional status, is important for empowerment and disease management [2]. Dietary guidance is considered important by 80–89% of patients with IBD; however, only 8–16% report receiving sufficient information, while 60–68% believe it would be very helpful to receive dietary advice [10]. Although patient education in IBD has not been shown to influence patients’ knowledge and long-term psychosocial well-being [19], higher food-related quality of life scores were observed at follow-up than at baseline among participants who completed the study. However, because of the single-arm pre–post design, the absence of a control group, and the lack of information on disease activity and treatment changes during follow-up, these observed differences cannot be attributed to the intervention itself. Alternative explanations, including regression to the mean, temporal changes in clinical status, and increased awareness resulting from study participation, cannot be excluded. In addition, both tools were positively evaluated by participants. While the observed improvement in Fr-QoL was statistically significant and corresponded to a small-to-moderate effect size (Cohen’s d = 0.39), its clinical relevance remains uncertain because no validated minimally clinically important difference (MCID) has been established for this instrument.

Despite the provision of feedback on food intake, no improvements in diet quality were observed. High total fat intake was driven by elevated consumption of saturated fats. In addition, sugar intake was very high. These patterns are typical of a Western diet, which has been associated with the incidence of IBD [20]. Overall, the findings on dietary intake in the present study were comparable to those reported by Peters et al. [21]. Moreover, fiber intake was low at baseline (mean of 26.3 [SD 11.0]) and remained low after the intervention (23.3 [SD 12.0]). However, this is higher than the average fiber intake reported in IBD patients, which ranges from 9.9 ± 7.8 g/day to 21.0 ± 10.5 g/day, as described in a systematic review by Day et al. [22]. An additional consideration is that digital dietary feedback may have unintended effects. Patients with IBD frequently avoid foods perceived to trigger symptoms, and feedback on dietary intake without direct professional guidance may inadvertently reinforce existing dietary restrictions. Consequently, the observed reductions in reported energy, fiber, and several micronutrients should not automatically be interpreted as beneficial dietary changes. Several explanations may account for these findings. First, patients with IBD frequently restrict foods that they perceive to trigger symptoms, and personalized dietary feedback may have reinforced existing avoidance behaviors in some participants. Second, repeated completion of the FFQ may have increased awareness of dietary intake, resulting in more accurate reporting at follow-up rather than true changes in food consumption. Third, seasonal or other time-related factors may have influenced dietary habits during the study period. Finally, the intervention primarily provided information and feedback and did not include structured behavioral counseling or routine dietitian follow-up for most participants. This level of support may have been insufficient to translate increased awareness into sustained improvements in dietary quality or nutrient intake. As this study was designed as an online implementation study rather than a clinical care pathway, information on malnutrition risk, unintentional weight loss, supplement use, and adverse events was not collected. Future studies should investigate whether implementation of digital dietary tools in combination with dietitian-led support may help optimize dietary intake while minimizing the risk of unnecessary dietary restriction. A borderline positive association between the animal protein dietary pattern and Fr-QoL score was observed. This finding may warrant further investigation, as protein intake is often reported to be suboptimal in patients with IBD [21]. The observed reductions in energy intake and several macro- and micronutrients should be interpreted with caution. Although these findings may represent genuine dietary changes following receipt of personalized dietary feedback, dietary intake was assessed using a self-reported FFQ and is therefore susceptible to measurement error and reporting bias. Repeated completion of the FFQ may have increased participants’ awareness of their dietary habits, potentially influencing reporting behavior. In addition, seasonal variation and other time-related factors may have contributed to the observed differences. According to the Goldberg criteria, 26% of completers were classified as under-reporters. In addition, no differences were observed between completers and non-completers in the prevalence of under-reporting, baseline BMR, or EI:BMR ratios. These findings suggest that baseline dietary reporting characteristics were broadly comparable between completers and non-completers and therefore provide limited reassurance against differential attrition related to dietary misreporting. However, these analyses do not provide information on whether reporting behavior changed between baseline and follow-up among study completers. Consequently, increased under-reporting at follow-up cannot be excluded as a potential explanation for the observed reductions in reported energy and nutrient intakes. Notably, BMI remained stable during the study period despite the reported reduction in energy intake, indicating that the observed changes should be interpreted as reductions in reported dietary intake rather than definitive evidence of true behavioral change.

Data from the GINQ-FFQ were compared with EFSA dietary reference values and provided as feedback to the participants. To facilitate understanding of their dietary intake, this feedback was presented using a slider. As this instrument could potentially be used for health-related advice, it was necessary to assess whether it complied with the criteria of the Medical Device Regulations Act—initially for use in the study and subsequently for implementation in routine care. Patients can now access the questionnaire and receive automated feedback via their personal email, allowing them to decide independently how to act on this information (Supplementary File S2). During the study, not all participants received or were aware of the knowledge tool, as it was distributed as a PDF by email. Consequently, not all patients used the instrument. The tool is currently available through a dedicated website (Supplementary File S1). Furthermore, intervention exposure varied considerably across participants. Exploratory analyses indicated that consultation with an IBD dietitian was associated with higher follow-up Fr-QoL scores, whereas frequency of knowledge tool use and perceived usefulness of the tool were not significantly associated with follow-up Fr-QoL. These findings should be interpreted cautiously, as several exposure categories contained relatively small numbers of participants, limiting the feasibility of robust subgroup analyses. Data regarding actual use of the personalized feedback reports and external nutrition resources were not available and therefore could not be evaluated.

### Strengths and Limitations

A major strength of this study was the development of several deliverables for patients with IBD who wished to learn more about nutrition. First, the study included the training of specialized IBD dietitians, who were registered in a national directory that was accessible to both patients and healthcare providers. Second, a digital tool was developed to assess dietary intake and provide personalized feedback. Third, evidence-based nutritional information was made more accessible through the development of an IBD knowledge tool. The content of this tool can be updated as new evidence becomes available.

Despite these strengths, several limitations should be acknowledged. First, this implementation study was not powered for confirmatory hypothesis testing because no formal sample size calculation was performed. Therefore, the observed associations and changes over time should be interpreted as exploratory and hypothesis-generating rather than confirmatory. Second, the pre–post design without a control group limits the ability to attribute observed changes to the intervention itself. Alternative explanations for the observed improvement in Fr-QoL include regression to the mean, temporal changes in disease activity or treatment, and increased awareness resulting from study participation. Therefore, causal conclusions regarding the effectiveness of the intervention cannot be drawn. Third, we did not formally assess whether participants reviewed, understood, or implemented the personalized feedback provided. Therefore, the degree of participant engagement with the feedback and its influence on dietary behavior could not be determined. In addition, exposure to the intervention varied across participants, and several exposure groups were relatively small. Consequently, subgroup analyses comparing different levels of intervention exposure were underpowered and should be interpreted with caution. Fourth, although food frequency questionnaires generally provide a reliable estimate of dietary intake, respondents may not recognize all food items included in the questionnaire or may fail to report certain foods they consume. In addition, completion of the GINQ-FFQ required approximately 45 min, which may have been burdensome for some participants. Fifth, detailed clinical characteristics were not collected at baseline or follow-up. Consequently, we were unable to account for potential changes in disease activity, treatment, or other disease-related factors during the study period. Since such factors may influence both dietary intake and food-related quality of life, their contribution to the observed changes could not be assessed. Sixth, participants were recruited on a voluntary basis through patient organizations and healthcare professionals. As a result, the study population may have consisted of individuals with a greater interest in nutrition, digital health tools, and self-management than the broader IBD population. Therefore, the generalizability of the findings may be limited. Seventh, participant attrition may have introduced bias. Of the 147 participants who completed the baseline assessment, 108 completed all follow-up questionnaires. Participants who completed follow-up were older and reported significantly higher baseline Fr-QoL scores than non-completers. In addition, completers had higher baseline intakes of potassium, magnesium and non-haem iron. These findings indicate the presence of attrition bias and suggest that participants with poorer food-related quality of life were less likely to remain in the study. Consequently, the observed improvement in Fr-QoL may overestimate the effect that would have been observed in the full baseline cohort. Although sensitivity analyses using a linear mixed-effects model yielded results consistent with the complete-case analysis, bias related to selective dropout cannot be completely excluded. Eighth, multiple statistical comparisons were performed without formal adjustment for multiplicity. Consequently, some statistically significant findings may represent chance findings and should therefore be interpreted cautiously. Ninth, dietary patterns were identified using principal component analysis (PCA), an exploratory data-driven method. Although the data met commonly accepted criteria for PCA (KMO = 0.636; Bartlett’s test *p* < 0.001), the moderate sampling adequacy and relatively small sample size may have limited the stability and reproducibility of the identified dietary patterns. Therefore, these findings should be interpreted cautiously and validated in larger independent cohorts. Tenth, although statistically significant improvements in Fr-QoL were observed, the clinical relevance of these changes remains uncertain because no established minimal clinically important difference (MCID) is currently available for the Fr-QoL instrument used in this study.

## 5. Conclusions

In this single-arm pre–post study, food-related quality of life scores were higher at follow-up than at baseline among patients with IBD. However, no improvements in dietary intake or overall diet quality were observed. The observed reductions in macro- and micronutrient intake highlight the challenges associated with maintaining adequate dietary intake in this population. The findings support the feasibility and acceptability of the developed tools to support dietary assessment and improve access to nutrition-related information, although their effectiveness requires further evaluation in controlled studies. These findings suggest that additional strategies, including structured, dietitian-led support with digital resources, may be required to optimize nutritional outcomes in patients with IBD.

## Figures and Tables

**Table 1:** Baseline characteristics.

	Baseline Participants	Baseline Participants Completing Complete Study	Results After 6 Months	*p*-Value *
	*N* = 147	*N* = 108	*N* = 108	
Sex				
men	51 (34.7%)	36 (33%)		
women	96 (65.3%)	72 (67%)		
Age (years)	42.7 ± 15.4	44.5 ± 15.8		
IBD type				
CD	79 (53.7%)	58 (53.2%)		
UC	63 (42.9%)	46 (42.2%)		
IBDU	4 (2.7%)	4 (3.7%)		
Duration IBD	Mean 13.4 ± 12.3	Mean 13.1 ± 12.6		
CD	14.5	14.9		
UC	12.9	11.8		
IBDU	7.3	6.8		
BMI	25.1 ± 5.1	25.0 ± 4.8	25.3 ± 5.3	*p* = 0.635
Physical activity				*p* = 0.727 **
Level 1	26	19	20	
Level 2	73	56	49	
Level 3	37	28	35	
Level 4	11	5	4	
Fr-QoL	88.7 ± 21.8	91.5 ± 22.6	99.7 ± 22.5	***p* < 0.001**

* *p*-value paired *t*-test before-after participants completing complete study ** ANOVA between groups Abbreviations: BMI, Body Mass Index; Fr-QoL, Food-Related Quality of Life; mean ± standard deviation. Values in bold indicate statistically significant results (*p* < 0.05).

**Table 2:** Multiple linear regression analysis of diet-related factors associated with follow-up Fr-QoL (*N* = 108).

Variable	B	SE	β	*p*-Value
Diet use	−11.69	4.85	−0.238	0.018
KT weight	−3.50	4.22	−0.103	0.409
KT use	0.71	2.60	0.034	0.786

Model fit: R^2^ = 0.077; F(3,96) = 2.67; *p* = 0.052. Abbreviations: Fr-QoL, Food-Related Quality of Life; SE, standard error; β, standardized regression coefficient. Diet use: self-reported consultation with an IBD dietitian. KT weight: agreement with the statement “*Using the knowledge tool helps me better adapt my diet*.” KT use: self-reported frequency of knowledge tool use.

**Table 3:** Nutrient intake at baseline and after 6 months.

Nutrient		All Baseline Participants	Baseline Participants Completing Both FFQs	Results After 6 Months	*p*-Values *	*p*-Values **
		*N* = 147	*N* = 108	*N* = 108	All Baseline Participants vs. Participants After 6 Months	Pre–Post (6-Month) Difference in Participants Completing Both FFQs
		51 Men	36 Men	36 Men		
		96 Women	72 Women	72 Women		
Energy [kJ]	total	8568 ± 2831	8693 ± 2672	7671 ± 2473	0.022	**<0.001**
Energy [kcal]	total	2047 ± 676	2077 ± 637	1832 ± 829	0.021	**<0.001**
	men	2429 ± 687	2450 ± 729	2269 ± 996	0.298	0.059
	women	1844 ± 577	1897 ± 501	1670 ± 685	0.056	**0.003**
Energy [kcal/kg]	total	27.1 ± 9.23	27.5 ± 8.73	24.2 ± 10.5	0.015	**<0.001**
	men	29.7 ± 9.28	29.4 ± 9.44	25.4 ± 9.70	0.125	**0.025**
	women	25.7 ± 8.95	26.5 ± 8.27	23.6 ± 10.4	0.106	**0.004**
Total protein [g]		71.6 ± 28.5	72.1 ± 25.9	66.5 ± 31.2	0.170	**0.024**
Protein [g/kg]		0.947 ± 0.371	0.953 ± 0.349	0.875 ± 0.394	0.129	**0.014**
	men	1.01 ± 0.42	1.06 ± 0.42	0.95 ± 0.41	0.262	0.052
	women	0.88 ± 0.34	0.90 ± 0.30	0.85 ± 0.38	0.345	0.122
Protein plant [g]		31.5 ± 14.7	32.4 ± 14.4	28.5 ± 15.8	0.113	**0.001**
Protein animal [g]		40.0 ± 22.8	39.7 ± 19.2	37.9 ± 20.6	0.443	0.265
Total fat [g]		96.6 ± 33.9	97.2 ± 31.3	84.9 ± 39.7	0.010	**<0.001**
Sat. fatty acids [g]		29.4 ± 12.5	29.5 ± 11.9	26.1 ± 14.5	0.050	**0.004**
Ω-3-fatty acids [g]		2.87 ± 1.42	2.90 ± 1.39	2.53 ± 1.47	0.062	**0.008**
Ω-6-fatty acids [g]		14.5 ± 6.08	14.6 ± 5.99	12.5 ± 6.65	0.014	**<0.001**
Carbohydrate [g]		203 ± 83.5	208 ± 83.4	183 ± 96.7	0.075	**0.001**
Sugar [g]		86.1 ± 39.1	90.4 ± 39.2	80.1 ± 45.7	0.250	**0.004**
Starch [g]		117 ± 55.8	118 ± 54.7	103 ± 60.3	0.055	**0.002**
Fiber [g]		25.2 ± 11.3	26.3 ± 11.0	23.3 ± 12.0	0.173	**<0.001**
Alcohol [g]		3.50 ± 5.99	3.07 ± 5.37	2.80 ± 5.49	0.334	0.411
Vitamin A_RAE [mcg]		826 ± 384	859 ± 386	770 ± 421	0.266	**0.018**
Vitamin B1 [mg]		0.87 ± 0.33	0.89 ± 0.305	0.79 ± 0.37	0.079	**0.004**
Vitamin B2 [mg]		1.13 ± 0.46	1.15 ± 0.446	1.08 ± 0.52	0.383	0.091
Vitamin B6 [mg]		1.52 ± 0.54	1.52 ± 0.54	1.36 ± 0.58	0.034	**0.003**
Folic acid [mcg]		255 ± 89.1	261 ± 80.3	230 ± 94.0	0.026	**<0.001**
Vitamin B12 [mcg]		3.61 ± 1.87	3.68 ± 1.74	3.56 ± 1.96	0.845	0.451
Vitamin C [mg]		92.5 ± 46.8	92.5 ± 46.9	79.3 ± 48.0	0.034	**0.002**
Vitamin D [mcg]		2.56 ± 1.42	2.59 ± 1.39	2.38 ± 1.46	0.303	0.087
Sodium [mg]		1715 ± 700	1715 ± 701	1538 ± 867	0.088	**0.012**
Potassium [mg]		2989 ± 947	2990 ± 947	2690 ± 1065	0.24	**0.001**
Calcium [mg]		847 ± 372	877 ± 380	832 ± 450	0.762	0.228
Magnesium [mg]		329 ± 126	339 ± 125	309 ± 138	0.221	**0.005**
Iron [mg]		10.2 ± 3.91	10.4 ± 3.8	9.28 ± 4.28	0.073	**<0.001**
Haem [mg]		0.665 ± 0.675	0.613 ± 0.478	0.581 ± 0.478	0.253	0.403
Non-haem [mg]		9.51 ± 3.71	9.82 ± 3.64	8.68 ± 4.06	0.087	**<0.001**
Zinc [mg]		9.59 ± 3.47	9.59 ± 3.48	8.81 ± 4.16	0.119	**0.020**
Iodine [mcg]		135 ± 62.2	137 ± 60.5	128 ± 66.2	0.372	0.109

* *t*-test between all participants at baseline and 108 after 6 months; ** paired *t*-test between all participants completing both questionnaires. Abbreviations: g, gram; mg, milligram; mcg, microgram; RAE, Retinol Activity Equivalents. Values in bold indicate statistically significant results (*p* < 0.05).

**Table 4:** Topics included in the “Nutrition and IBD” knowledge tool and their frequency of access.

Topic in Knowledge Tool “Nutrition and IBD”	Times Used
How can I ensure adequate intake of dietary fiber?	48
How can I ensure sufficient protein intake?	40
Which foods may help to prevent inflammation?	37
What is IBD?	33
Which foods can cause bloating and flatulence?	32
What can I do if dairy products are less well tolerated?	28
Are there benefits to using omega-3 supplements or fish oil?	24
Are there benefits to using probiotics in the treatment of IBD?	22
How can I ensure my diet contains enough iron?	21
Which types of fat are best to consume if I have IBD?	21
What is the microbiome and what is its function?	20
Should I take additional vitamin supplements?	20
Should I eliminate gluten from my diet?	19
How can I obtain sufficient nutrients if I follow a vegetarian diet?	15
What is the FODMAP diet and is it advisable to use it?	15
Advice for fear of eating certain foods	14
What can I do in case of diarrhea?	14
Where can I find a dietitian specialized in nutrition for IBD?	14
Is it advisable to use turmeric?	13
What can I do in case of constipation?	11
When should I consult a dietitian?	10
What is the CDED and is it suitable for me?	8
What should I pay attention to in my diet if I have a stoma?	7
What is bile acid diarrhea and what can I do about it?	5
What can I eat if I have an intestinal narrowing (stenosis)?	5
What should I consider in my diet when traveling on holiday?	5
Are there specific dietary considerations if I have a pouch?	3

**Table 5:** Representative participant quotes regarding the perceived impact of the “Nutrition and IBD” knowledge tool on dietary management.

	Remark
1	“My IBD was already well controlled.”
2	“I was unable to find the tool.”
3	“I am already aware of my dietary triggers.”
4	“Neutral impact, as my symptoms have been stable and I already know what to avoid.”
5	“There is still room for dietary improvement, but this is mainly personal.”
6	“The project made me more aware of what I should and should not eat.”
7	“I am more mindful of fat intake and protein consumption.”
8	“The nutritional feedback helped me better guide my food choices.”
9	“I now understand which foods to avoid, which increases my motivation.”
10	“The project helped me create a clearer plan with my dietitian.”

## Data Availability

The original contributions presented in this study are included in the article and Supplementary Materials. Further inquiries can be directed to the corresponding author.

## Supplementary Materials

The following supporting information can be downloaded at: https://www.mdpi.com/article/10.3390/nu18162626/s1, Supplementary File S1. Nutritional Knowledge Tool “Nutrition and IBD” (Dutch version). This file contains the Dutch-language educational tool used in the intervention. The tool provides information on nutrition and inflammatory bowel disease (IBD), including healthy dietary patterns, nutrition during disease flares, prevention of nutritional deficiencies, and practical dietary recommendations. The Dutch version is provided because it was the version used by study participants. Supplementary File S2. Example of a personalized GINQ-FFQ feedback report (Dutch version). This file contains an example of the individualized dietary feedback provided to participants based on responses to the Groningen IBD Nutritional Questionnaire–Food Frequency Questionnaire (GINQ-FFQ). The report includes information on dietary intake, nutrient intake, adherence to dietary recommendations, and personalized dietary advice. The Dutch version is provided because it was the version used by study participants.

## Abbreviations

The following abbreviations are used in this manuscript:

IBD	Inflammatory bowel disease
CD	Crohn’s disease
UC	Ulcerative colitis
IBDU	Inflammatory bowel disease unclassified
BMI	Body Mass Index
Fr-QoL	Food-related quality of life
IBS	Irritable bowel syndrome
FODMAP	Fermentable Oligosaccharides, Disaccharides, Monosaccharides, And Polyols
EEN	Exclusive Enteral Nutrition
GINQ-FFQ	Groningen IBD Nutritional Questionnaire–Food Frequency Questionnaire
FAQs	Frequently asked questions
EFSA	European Food Safety Authority
SD	Standard deviation
IQR	Interquartile range
DII	Dietary inflammatory index
PCA	Principal Component Analysis
aMED	Alternate Mediterranean diet score

## Appendix A

**Table A1:** Topics included in the “Nutrition and IBD” knowledge tool.

What is IBD?
2. Fear of eating certain foods
3. The gut microbiome
4. Dietary fiber intake
5. Protein intake
6. Iron intake
7. Dairy intolerance
8. Dietary fats
9. Vegetarian diets
10. Foods associated with bloating and flatulence
11. Gluten-free diets
12. Diet and intestinal inflammation
13. Diarrhea
14. Bile acid diarrhea
15. Constipation
16. Intestinal strictures
17. Nutrition and stoma care
18. Nutrition and ileal pouch management
19. Vitamin supplementation
20. Probiotics
21. Omega-3 supplements and fish oil
22. Turmeric
23. FODMAP diet
24. Crohn’s Disease Exclusion Diet (CDED)
25. Nutrition during travel
26. When to consult a dietitian
27. How to find an IBD-specialized dietitian

**Table A2:** Comparison of baseline characteristics between completers and non-completers (attrition analysis).

		Baseline Participants Completing Both FFQs	Baseline Participants Not Completing the Study	* *p*-Values
		*N* = 108	*N* = 39	Between Responders and Non-Responders
Sex	men	36 (33%)	15 (38%)	0.567
	women	72 (67%)	24 (62%)	
Age [years]		44.5 ± 15.8	38.8 ± 12.9	**0.049**
IBD type	CD	58 (53.2%)	23 (59%)	0.448
	UC	46 (42.2%)	16 (41%)	
	IBDU	4 (3.7%)	0 (0%)	
Duration IBD	Mean	13.1 ± 12.6	11.7 ± 9.8	0.306
	CD	14.9	10.8	
	UC	11.8	13.1	
	IBDU	6.8	0	
BMI		25.0 ± 4.8	24.6 ± 4.0	0.578
Physical activity	Level 1	19	7	0.387
	Level 2	56	18	
	Level 3	28	9	
	Level 4	5	5	
Fr-QoL		91.5 ± 22.6	79.9 ± 21.7	**0.005**
Energy [kJ]	total	8693 ± 2672	8469 ± 3193	0.802
Energy [kcal]	total	2077 ± 637	2024 ± 763	0.807
	men	2450 ± 729	2417 ± 586	0.936
	women	1897 ± 501	1779 ± 786	0.525
Energy [kcal/kg]	total	27.5 ± 8.73	26.7 ± 10.2	0.771
	men	29.4 ± 9.44	31.2 ± 9.00	0.483
	women	26.5 ± 8.27	24.0 ± 10.1	0.269
Basal metabolic rate		1520 ± 267	1554 ± 272	0.505
Energy intake/BMR		1.37 ± 0.40	1.30 ± 0.46	0.393
Total protein [g]		72.1 ± 25.9	70.2 ± 33.2	0.734
Protein [g/kg]	total	0.953 ± 0.349	0.926 ± 0.407	0.699
	men	1.06 ± 0.42	1.10 ± 0.43	0.768
	women	0.90 ± 0.30	0.82 ± 0.37	0.287
Protein plant [g]		32.4 ± 14.4	29.5 ± 14.4	0.306
Protein animal [g]		39.7 ± 19.2	40.7 ± 29.8	0.265
Total fat [g]		97.2 ± 31.3	97.5 ± 39.4	0.856
Sat. fatty acids [g]		29.5 ± 11.9	29.6 ± 13.4	0.883
Ω-3-fatty acids [g]		2.90 ± 1.39	2.84 ± 1.51	0.903
Ω-6-fatty acids [g]		14.6 ± 5.99	14.6 ± 6.19	0.897
Carbohydrate [g]		208 ± 83.4	197 ± 87.2	0.613
Sugar [g]		90.4 ± 39.2	80.4 ± 44.3	0.289
Starch [g]		118 ± 54.7	116 ± 59.4	0.989
Fiber [g]		26.3 ± 11.0	22.3 ± 11.0	0.057
Alcohol [g]		3.07 ± 5.37	4.26 ± 7.15	0.355
Vitamin A_RAE [mcg]		859 ± 386	740 ± 343	0.104
Vitamin B1 [mg]		0.89 ± 0.305	0.81 ± 0.37	0.207
Vitamin B2 [mg]		1.15 ± 0.446	1.14 ± 0.57	0.945
Vitamin B6 [mg]		1.52 ± 0.54	1.45 ± 0.76	0.624
Folic acid [mcg]		261 ± 80.3	239 ± 105	0.195
Vitamin B12 [mcg]		3.68 ± 1.74	3.41 ± 2.12	0.448
Vitamin C		92.5 ± 46.9	79.7 ± 48.5	0.143
Vitamin D [mcg]		2.59 ± 1.39	2.56 ± 1.64	0.984
Sodium [mg]		1715 ± 701	1715 ± 844	0.899
Potassium [mg]		2990 ± 947	2626 ± 985	**0.041**
Calcium [mg]		877 ± 380	783 ± 327	0.212
Magnesium [mg]		339 ± 125	294 ± 114	**0.044**
Iron [mg]		10.4 ± 3.8	9.43 ± 3.88	0.162
Haem [mg]		0.613 ± 0.478	0.777 ± 1.02	0.231
Non-haem [mg]		9.82 ± 3.64	8.64 ± 3.58	**0.044**
Zinc [mg]		9.59 ± 3.48	9.24 ± 4.03	0.584
Iodine [mcg]		137 ± 60.5	134 ± 64.4	0.874

* *p*-values represent comparisons between participants who completed follow-up (*n* = 108) and those who did not complete follow-up (*n* = 39), using independent-samples t-tests for continuous variables and chi-square tests for categorical variables, as appropriate. Abbreviations: g, gram; mg, milligram; mcg, microgram; BMR, basal metabolic rate; RAE, Retinol Activity Equivalents. Values in bold indicate statistically significant results (*p* < 0.05).

**Table A3:** Fulfillment of EFSA guidelines among participants at baseline (*n* = 108).

Participant nr	Energy [kcal]	Protein [g/kg]	Total fat [en%)	Ω 3 fatty acids [g]	Saturated fat [en%]	Carbohydrates [en%]	Sugar [en%]	Fibres [g]	Alcohol [g]	Water [g]	Vitamin A RAE [mcg]	Vitamin B1 [mg]	Vitamin B2 [mg]	Vitamin B6 [mg]	Vitamin B11 [mcg]	Vitamin B12 [mcg]	Vitamin C [mg]	Vitamin D [mcg]	Sodium [mg]	Potassium [mg]	Calcium [mg]	Magnesium [mg]	Iron [mg]	Zinc [mg]	Iodine [mcg]
1	2	1	5	3	5	1	3	1	3	3	3	2	1	3	2	3	3	2	2	2	2	1	2	3	1
2	2	2	5	3	5	3	5	1	3	2	2	1	2	1	1	2	3	2	2	2	2	1	1	1	2
3	4	5	5	3	5	2	5	3	3	3	3	3	2	3	3	3	3	2	3	3	3	3	3	3	3
4	2	1	5	3	5	2	5	1	3	2	3	3	1	1	1	2	3	3	3	2	1	1	2	2	1
5	2	3	5	3	5	2	5	3	3	3	2	3	2	3	2	2	3	2	2	2	2	3	3	3	1
6	3	3	5	3	5	2	5	3	3	3	3	3	1	3	2	2	2	3	2	2	1	3	2	3	2
7	2	2	5	3	5	2	5	1	3	1	2	1	1	1	1	2	2	2	3	1	1	1	1	2	2
8	2	1	5	3	5	3	5	2	3	2	2	3	1	3	1	2	3	2	2	2	1	2	2	3	2
9	2	2	5	3	5	3	5	1	3	3	3	2	1	3	2	2	3	2	3	2	1	2	2	2	2
10	4	5	5	3	5	3	5	3	3	3	3	3	3	3	3	3	3	3	5	3	3	5	3	3	3
11	3	3	5	3	5	3	5	3	3	2	3	3	2	3	3	3	3	3	3	3	1	3	3	3	3
12	2	2	5	3	5	2	5	2	3	2	3	3	2	3	2	3	3	2	3	2	2	2	2	3	2
13	2	2	5	3	5	2	5	2	3	3	3	3	2	3	2	3	2	3	5	2	2	2	2	3	2
14	2	2	5	3	5	3	5	1	3	2	2	3	1	3	1	2	3	2	2	2	1	1	2	2	1
15	4	5	5	3	5	2	5	3	3	3	3	3	3	3	3	3	3	2	5	3	3	5	5	3	3
16	2	1	5	3	5	2	5	1	3	3	2	1	2	1	1	2	1	2	2	2	2	1	1	1	1
17	2	2	5	3	5	2	5	1	3	2	2	2	2	3	1	3	3	2	2	2	2	1	2	3	1
18	2	3	5	3	5	2	5	3	5	3	2	3	2	3	2	3	3	2	2	3	1	3	3	3	2
19	2	3	5	3	5	3	5	3	3	3	2	3	2	3	3	1	3	2	3	3	2	3	3	3	3
20	3	2	5	3	5	1	5	1	5	3	3	2	2	3	2	3	3	2	3	2	2	2	2	3	2
21	2	2	5	3	5	2	5	1	3	3	2	2	1	1	1	3	1	3	2	1	1	1	2	2	2
22	4	5	5	3	5	2	5	1	3	3	3	3	2	3	2	3	3	2	3	2	2	3	3	3	2
23	2	3	5	3	5	2	5	1	3	3	3	3	1	3	1	2	3	2	3	2	1	2	2	3	2
24	4	3	5	3	5	2	5	2	3	3	3	3	2	3	2	3	3	3	3	2	1	3	3	3	1
25	4	3	5	3	5	2	5	3	3	3	3	3	3	3	3	3	3	3	5	3	3	3	3	3	3
26	4	3	5	3	5	2	5	1	5	3	3	3	2	3	3	3	3	3	3	3	2	3	3	3	2
27	2	3	5	3	5	2	5	1	3	3	2	3	1	3	2	2	3	2	2	2	1	1	2	2	1
28	4	2	5	3	5	3	5	3	3	3	2	2	1	3	2	1	3	2	3	2	1	3	3	3	3
29	2	5	5	3	5	3	5	3	5	3	3	3	3	3	3	3	3	3	5	3	3	3	3	3	3
30	3	2	5	3	5	3	5	2	3	3	3	3	2	3	3	3	3	2	2	2	2	2	2	3	2
31	2	1	5	3	5	3	5	1	3	3	3	1	1	1	1	1	2	2	3	1	1	1	2	1	1
32	2	3	5	3	5	3	5	3	3	3	3	3	1	3	3	1	2	2	2	3	1	5	3	3	1
33	2	2	5	3	5	2	5	2	3	2	3	3	2	1	1	2	3	3	2	2	2	3	2	3	2
34	3	2	5	3	5	3	5	3	3	3	2	3	2	3	3	3	3	2	2	3	1	3	3	3	1
35	2	1	5	3	5	2	5	1	3	3	3	2	1	3	1	2	3	2	2	2	1	1	2	2	1
36	2	5	5	3	5	3	5	3	3	4	3	3	3	3	3	3	3	2	5	3	3	5	3	3	3
37	2	1	5	3	5	2	5	1	3	2	2	2	2	1	1	3	3	2	2	2	2	1	1	2	2
38	2	2	5	3	5	2	5	1	5	3	2	3	2	3	1	3	3	3	3	2	2	2	2	3	2
39	2	2	5	3	5	1	5	1	3	3	2	2	2	3	2	3	3	2	2	2	1	1	2	3	1
40	2	3	5	3	5	2	5	1	3	2	3	3	1	3	1	2	2	2	3	2	1	2	2	3	2
41	2	1	5	3	5	2	5	1	3	2	2	2	1	3	2	1	3	2	1	2	1	1	2	1	1
42	2	3	5	3	5	3	5	1	3	2	3	3	2	3	2	3	3	2	3	2	2	2	2	3	3
43	4	3	5	3	5	2	5	2	5	3	3	3	2	3	2	3	3	3	3	3	2	3	2	3	2
44	2	1	5	3	5	3	5	2	3	3	2	3	1	3	2	2	3	2	3	2	1	3	2	3	2
45	4	5	5	3	5	2	5	2	3	3	3	3	2	3	2	3	3	2	5	2	3	3	3	3	3
46	2	2	5	3	5	3	5	1	3	3	3	3	2	3	1	3	3	3	3	2	1	2	2	3	2
47	2	2	5	3	5	2	5	1	3	3	3	2	2	3	2	3	3	2	2	2	3	3	2	3	3
48	2	3	5	3	5	2	5	1	3	3	2	3	1	3	2	3	3	2	3	2	1	3	2	3	3
49	2	5	5	3	5	3	5	3	3	2	3	3	2	3	3	3	3	2	5	3	1	3	3	3	3
50	2	1	5	3	5	2	5	1	3	2	3	2	2	1	1	3	2	2	3	2	2	1	1	3	1
51	2	3	5	3	5	3	5	1	3	2	2	3	1	3	2	3	3	2	3	2	1	1	2	3	2
52	2	3	5	3	5	3	5	1	3	3	3	2	1	3	1	1	3	3	2	2	1	1	2	1	2
53	3	2	5	3	5	3	5	3	3	3	3	3	2	3	2	3	3	2	3	3	3	3	3	3	2
54	4	5	5	3	5	2	5	3	3	3	3	3	3	3	3	3	3	3	5	3	3	3	3	3	3
55	4	1	5	3	5	3	5	1	3	3	3	3	2	3	2	2	3	2	3	2	2	3	2	3	3
56	2	1	5	3	5	3	5	1	3	3	2	2	1	1	1	1	3	2	2	2	1	1	2	1	2
57	2	1	5	3	5	1	5	1	3	3	3	2	1	1	1	2	3	2	2	2	1	1	2	1	1
58	2	2	5	3	5	2	5	2	3	3	2	3	1	3	2	2	3	2	2	2	1	3	2	3	1
59	2	2	5	3	5	1	5	1	3	2	3	3	2	3	2	3	3	3	3	2	1	1	2	3	1
60	2	2	5	3	5	3	5	1	3	2	3	1	1	1	1	3	2	2	2	1	1	1	1	1	1
61	2	2	5	3	5	2	5	1	3	3	3	3	2	3	2	3	3	2	3	2	3	2	2	3	2
62	4	5	5	3	5	3	5	3	3	3	3	3	3	3	3	3	3	3	5	3	3	3	3	3	3
63	4	3	5	3	5	3	5	2	3	4	3	3	3	3	2	3	3	3	5	2	3	3	2	3	2
64	2	3	5	3	5	2	5	1	3	2	2	2	1	3	2	1	3	3	3	2	1	1	2	2	1
65	4	3	5	3	5	2	5	3	3	3	3	3	2	3	3	3	3	2	5	3	3	3	2	3	2
66	2	2	5	3	5	1	5	1	5	2	3	2	1	3	2	3	3	2	2	2	1	1	2	3	1
67	2	5	5	3	5	1	5	1	3	3	3	3	3	3	2	3	3	3	5	3	3	3	3	3	2
68	2	3	5	3	5	2	5	1	3	2	3	3	2	3	1	3	2	3	3	2	3	1	2	3	1
69	2	2	5	3	5	2	5	1	3	3	3	2	1	3	1	3	2	3	3	2	1	2	2	3	2
70	2	3	5	3	5	1	5	1	3	3	3	3	2	3	2	3	3	3	3	2	2	3	2	3	3
71	2	2	5	3	5	2	5	1	3	2	2	2	1	1	1	3	2	2	3	2	3	1	2	3	1
72	3	5	5	3	5	2	5	1	3	3	3	3	3	3	3	3	3	3	3	3	3	3	3	3	3
73	2	3	5	3	5	3	5	2	3	3	3	3	2	1	3	3	3	2	2	2	3	3	2	3	2
74	2	5	5	3	5	1	5	1	3	3	3	3	2	3	2	3	3	3	3	2	2	3	2	3	2
75	2	5	5	3	5	2	5	2	3	2	2	3	2	3	3	3	3	2	5	2	2	2	3	3	3
76	2	5	5	3	5	3	5	3	3	3	2	3	1	3	3	1	3	2	3	2	1	3	3	3	2
77	4	5	5	3	5	3	3	2	3	2	3	2	2	3	2	3	2	3	5	2	3	3	3	3	3
78	2	3	5	3	5	3	5	1	3	3	2	3	1	3	2	3	3	3	3	2	2	3	2	3	2
79	4	5	5	3	5	1	5	3	3	3	3	3	2	3	3	3	3	2	3	3	3	5	3	3	2
80	2	2	5	3	5	3	5	3	5	2	2	3	1	3	2	2	3	2	2	2	1	3	3	3	1
81	4	5	5	3	5	3	5	3	3	3	3	3	3	3	3	3	3	2	5	3	3	3	3	3	3
82	2	1	5	3	5	3	5	1	3	3	2	3	1	3	2	1	3	2	3	2	2	1	2	3	1
83	4	5	5	3	5	3	5	2	5	3	3	3	3	3	3	3	3	3	3	3	3	3	3	3	3
84	2	5	5	3	5	3	5	3	5	3	3	3	2	3	2	3	3	3	3	3	3	3	3	3	3
85	2	2	5	3	5	2	5	2	3	3	3	2	1	1	2	1	3	2	2	2	2	2	2	3	2
86	4	2	5	3	5	1	5	3	3	3	3	3	2	3	2	3	3	3	3	2	2	3	3	3	2
87	2	1	5	3	5	2	3	1	3	3	3	3	2	3	2	3	3	2	3	3	1	2	2	3	1
88	3	1	5	3	5	3	5	2	3	3	3	3	1	3	1	2	3	2	3	2	1	3	2	3	2
89	2	3	5	3	5	3	5	2	3	3	3	3	2	3	2	3	3	3	5	2	2	3	3	3	3
90	3	5	5	3	5	1	3	2	3	2	3	3	1	3	2	3	3	2	2	2	2	3	2	3	1
91	2	1	5	3	5	3	5	1	3	2	2	2	1	3	2	1	3	2	2	2	1	2	2	1	1
92	2	2	5	3	5	2	5	1	3	2	3	3	1	3	2	3	3	3	3	2	1	2	2	2	1
93	2	5	5	3	5	3	5	3	3	3	3	3	1	3	3	3	3	2	3	2	1	3	3	3	3
94	2	1	5	3	5	3	5	1	3	3	2	2	1	1	2	3	3	2	2	2	1	2	2	2	2
95	4	3	5	3	5	2	5	2	3	3	3	3	2	3	2	2	3	2	3	2	3	3	2	3	2
96	2	3	5	3	5	2	5	2	3	2	3	3	2	3	2	2	3	2	2	2	2	3	2	3	2
97	3	3	5	3	5	3	5	3	3	2	3	3	1	3	2	3	3	2	3	2	1	3	3	3	3
98	2	5	5	3	5	2	5	1	3	3	2	2	1	3	2	3	3	2	2	2	1	2	2	2	1
99	2	5	5	3	5	3	5	3	3	3	2	3	2	3	3	3	3	2	3	2	2	3	3	3	3
100	2	3	5	3	5	2	5	3	3	3	3	3	2	3	3	3	3	3	3	3	3	3	3	3	2
101	3	5	5	3	5	2	5	1	3	2	3	3	2	3	2	3	3	2	5	2	2	1	2	3	2
102	2	1	5	3	5	2	5	1	3	3	3	2	1	1	2	2	3	3	3	2	2	1	2	2	1
103	2	1	5	3	5	3	5	1	3	3	2	2	1	3	1	1	3	2	2	2	1	2	2	1	2
104	4	5	5	3	5	3	5	3	3	3	3	3	2	3	3	2	3	3	5	3	2	3	3	3	3
105	4	3	5	3	5	3	5	2	3	3	3	3	2	3	2	3	3	2	3	2	2	2	2	3	2
106	4	5	5	3	5	2	5	3	3	3	3	3	2	3	3	3	3	2	3	3	1	3	3	3	1
107	2	5	5	3	5	1	5	2	5	3	3	3	2	3	3	3	3	3	3	3	1	3	3	3	3
108	2	5	5	3	5	3	5	3	5	3	3	3	2	3	2	3	3	3	3	3	3	3	3	3	3

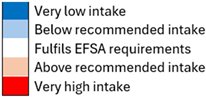

**Table A4:** Fulfillment of EFSA guidelines among participants after 6 months (*n* = 108).

Participant nr.	Energy [kcal]	Protein [g/kg]	Total fat [en%)	Ω 3 fatty acids [g]	Saturated fat [en%]	Carbohydrates [en%]	Sugar [en%]	Fibres [g]	Alcohol [g]	Water [g]	Vitamin A RAE [mcg]	Vitamin B1 [mg]	Vitamin B2 [mg]	Vitamin B6 [mg]	Vitamin B11 [mcg]	Vitamin B12 [mcg]	Vitamin C [mg]	Vitamin D [mcg]	Sodium [mg]	Potassium [mg]	Calcium [mg]	Magnesium [mg]	Iron [mg]	Zinc [mg]	Iodine [mcg]
1	3	2	5	3	5	1	5	2	3	3	3	3	1	3	2	3	3	2	2	2	2	3	2	3	1
2	2	2	5	3	5	2	5	1	3	2	2	1	2	1	1	2	2	2	2	2	2	1	1	2	2
3	4	5	5	3	5	3	5	3	3	3	3	3	2	3	3	3	3	2	3	3	3	3	3	3	3
4	4	3	5	3	5	2	5	1	3	2	3	3	2	3	1	3	3	3	5	2	1	2	2	3	2
5	2	3	5	3	5	2	5	2	3	3	2	3	2	3	2	3	2	2	2	2	2	3	2	3	2
6	2	1	5	3	5	3	5	1	3	2	2	2	1	1	1	3	2	3	2	2	1	2	2	3	2
7	2	3	5	3	5	2	5	1	3	2	3	2	2	1	1	3	2	2	3	2	3	1	1	3	3
8	2	1	5	3	5	3	5	3	3	3	2	3	2	3	1	2	3	2	3	3	2	3	3	3	2
9	2	2	5	3	5	2	5	1	3	3	2	3	1	1	2	1	3	2	3	2	1	2	2	2	2
10	4	5	5	3	5	3	5	3	5	3	3	3	3	3	3	3	3	3	5	3	3	3	3	3	3
11	3	3	5	3	5	3	5	3	3	2	3	3	2	3	2	3	3	3	3	3	2	3	3	3	3
12	2	2	5	3	5	3	5	1	3	2	2	3	2	3	1	3	2	2	3	2	2	2	2	3	2
13	2	2	5	3	5	1	3	1	5	2	3	3	2	3	1	3	2	3	5	2	1	2	2	3	2
14	2	1	5	3	5	3	5	1	3	2	2	1	1	1	1	1	2	3	2	2	1	1	1	1	1
15	2	5	5	3	5	3	5	3	3	3	2	3	2	3	2	3	3	2	5	3	2	3	3	3	3
16	2	1	5	3	5	2	5	1	3	3	2	1	1	1	1	2	3	2	2	2	1	1	1	1	2
17	4	3	5	3	5	2	5	1	3	2	2	3	2	3	2	3	3	2	3	2	2	3	2	3	3
18	2	2	5	3	5	2	5	2	5	3	2	3	1	3	2	3	3	2	2	2	1	3	2	3	1
19	2	2	5	3	5	3	5	3	3	3	3	3	1	3	2	1	3	2	2	3	1	3	3	3	2
20	4	5	5	3	5	2	3	2	5	3	3	3	3	3	2	3	3	3	5	3	3	3	3	3	3
21	2	1	5	3	5	2	5	1	3	2	2	1	1	1	1	3	2	2	2	2	1	1	2	2	1
22	2	3	5	3	5	3	5	1	3	3	3	3	1	3	2	2	3	2	3	2	1	1	3	3	1
23	2	2	5	3	5	2	5	1	3	3	3	3	1	3	1	2	3	2	2	2	1	2	2	3	1
24	2	3	5	3	5	2	5	1	3	3	3	2	2	3	2	3	3	3	3	2	2	1	2	3	2
25	4	3	5	3	5	2	5	3	3	3	3	3	3	3	2	3	3	3	5	3	3	3	3	3	3
26	4	2	5	3	5	3	5	1	3	3	3	3	2	3	2	3	3	3	3	3	2	3	2	3	2
27	2	3	5	3	5	2	5	1	3	3	3	2	2	3	1	3	3	3	2	2	2	2	2	3	2
28	2	1	5	3	5	3	5	1	3	3	2	1	1	3	1	1	3	2	2	2	1	1	2	1	1
29	2	2	5	3	5	2	5	1	3	2	3	1	1	1	1	3	2	2	2	2	1	1	2	2	1
30	2	2	5	3	5	3	5	1	3	3	2	2	2	1	2	3	3	2	2	2	2	2	2	3	2
31	2	2	5	3	5	3	5	1	3	3	3	3	1	3	2	2	3	2	3	2	1	2	2	2	1
32	2	3	5	3	5	3	5	3	3	3	2	3	1	3	2	1	3	2	2	3	1	5	3	3	1
33	3	3	5	3	5	2	5	1	3	2	3	2	2	1	2	3	3	3	3	2	3	3	2	3	3
34	2	3	5	3	5	3	5	3	3	3	2	3	1	3	2	1	3	2	2	3	1	3	2	3	2
35	2	1	5	3	5	2	5	1	3	3	2	2	1	1	1	3	2	2	2	2	1	1	2	2	1
36	2	5	5	3	5	3	5	3	3	3	3	3	3	3	3	3	3	2	3	3	3	3	3	3	3
37	2	1	5	3	5	3	5	1	3	2	2	3	1	1	1	2	3	2	2	2	2	2	2	2	2
38	2	3	5	3	5	2	5	1	3	3	3	3	2	3	2	3	3	3	3	2	3	2	2	3	3
39	1	1	5	2	5	3	5	1	3	1	1	1	1	1	1	1	1	2	1	1	1	1	1	1	1
40	2	2	5	3	5	3	5	1	3	2	3	2	1	1	1	2	2	2	3	2	1	1	2	2	2
41	2	1	5	3	5	2	5	1	3	3	3	3	3	3	2	3	3	3	2	2	2	3	2	3	2
42	2	3	5	3	5	3	5	1	3	3	3	3	3	3	2	3	3	2	3	3	3	3	2	3	3
43	2	3	5	3	5	2	5	1	3	3	3	3	1	3	2	3	3	3	3	2	2	2	2	3	2
44	2	1	5	3	5	2	5	1	3	3	2	3	2	3	2	3	3	2	2	2	2	3	2	3	2
45	4	5	5	3	5	2	5	1	5	3	3	3	2	3	2	3	3	2	3	2	3	3	3	3	3
46	2	2	5	3	5	3	5	1	3	2	3	2	2	3	2	3	3	3	3	2	1	2	2	3	3
47	2	2	5	3	5	3	5	1	3	2	2	2	2	3	1	3	3	2	2	2	2	2	2	3	2
48	2	3	5	3	5	2	5	1	3	3	2	2	2	3	1	3	3	2	2	2	1	1	2	3	2
49	2	5	5	3	5	3	5	3	3	1	3	3	2	3	3	3	3	3	5	3	1	3	3	3	3
50	2	1	5	3	5	3	5	1	3	3	2	2	1	1	1	2	2	2	2	2	2	1	1	2	1
51	2	2	5	3	5	3	5	1	3	2	2	2	1	3	1	2	3	3	2	2	1	1	2	2	1
52	2	3	5	3	5	3	5	1	3	3	2	3	1	3	2	1	3	3	2	2	1	2	2	2	2
53	2	1	5	3	5	3	5	1	3	3	2	2	2	3	2	2	3	2	2	2	2	2	2	2	2
54	4	3	5	3	5	2	5	1	5	3	3	3	3	3	2	3	3	3	3	2	3	3	2	3	3
55	2	1	5	3	5	3	5	1	3	3	2	2	1	3	1	1	3	2	2	2	2	2	2	3	2
56	2	1	5	3	5	3	5	1	3	3	2	1	1	1	1	1	3	2	2	2	1	1	1	1	1
57	2	3	5	3	5	1	5	1	3	3	3	2	1	3	2	3	3	2	2	2	1	2	2	3	1
58	2	2	5	3	5	2	5	2	3	3	2	3	1	3	2	1	3	2	2	2	1	3	2	3	1
59	3	3	5	3	5	2	5	1	3	3	3	3	3	3	2	3	3	3	5	2	3	3	2	3	3
60	2	3	5	3	5	2	5	1	3	2	3	1	1	3	1	2	3	3	3	2	1	1	2	2	1
61	2	3	5	3	5	2	5	2	3	3	3	3	2	3	3	3	3	2	3	2	3	3	2	3	2
62	4	5	5	3	5	2	5	3	3	3	3	3	3	3	3	3	3	2	5	3	3	3	3	3	3
63	4	5	5	3	5	3	5	3	3	4	3	3	3	3	3	3	3	3	5	3	3	3	3	3	3
64	2	3	5	3	5	2	5	1	3	3	2	2	1	3	1	2	3	2	3	2	1	1	2	3	2
65	4	3	5	3	5	3	5	3	3	3	3	3	2	3	3	3	3	2	3	3	2	3	2	3	3
66	2	2	5	3	5	1	5	1	5	2	3	2	2	3	2	3	2	3	2	2	1	2	2	3	2
67	2	5	5	3	5	1	5	1	3	3	3	3	2	3	3	3	3	3	3	3	3	3	3	3	2
68	3	3	5	3	5	2	5	1	3	2	3	3	2	3	1	3	2	3	3	2	3	1	2	3	2
69	2	2	5	3	5	2	5	1	3	3	3	3	2	3	2	3	3	3	3	2	2	2	2	3	2
70	2	2	5	3	5	1	5	1	3	2	3	2	2	3	1	3	3	3	2	2	2	1	1	3	2
71	2	3	5	3	5	2	5	1	3	3	3	3	2	1	2	3	3	2	5	2	3	3	2	3	3
72	2	2	5	3	5	1	5	1	3	2	3	2	1	3	2	3	3	2	2	2	1	2	2	3	1
73	2	3	5	3	5	3	5	2	3	3	3	3	1	3	2	2	3	2	2	2	1	3	2	3	2
74	3	5	5	3	5	2	5	3	3	3	3	3	2	3	3	3	3	2	3	3	2	3	3	3	2
75	2	5	5	3	5	3	5	2	3	3	3	3	2	3	2	3	3	3	3	2	2	2	3	3	3
76	4	5	5	3	5	3	5	3	3	3	2	3	2	3	3	3	3	2	3	3	2	5	3	3	3
77	3	5	5	3	5	2	5	2	3	2	3	3	2	3	2	3	2	3	5	2	3	2	3	3	3
78	2	3	5	3	5	3	5	1	3	3	3	3	2	3	2	3	3	2	3	2	2	3	2	3	2
79	4	5	5	3	5	2	5	3	3	3	3	3	3	3	3	3	3	2	5	3	5	5	3	5	3
80	2	2	5	3	5	3	5	3	3	2	2	3	1	3	2	1	3	2	3	2	1	3	3	3	2
81	2	5	5	3	5	3	5	3	3	3	3	3	2	3	3	3	3	2	3	3	1	3	3	3	3
82	2	1	5	3	5	2	5	1	3	2	3	3	1	3	1	3	3	3	3	2	1	1	2	3	1
83	2	5	5	3	5	2	5	3	3	3	3	3	2	3	3	3	3	2	3	3	3	3	3	3	3
84	3	3	5	3	5	2	5	1	3	3	3	3	3	3	2	3	3	2	3	2	3	3	2	3	3
85	2	3	5	3	5	3	5	3	3	2	3	3	1	1	2	1	3	2	3	2	2	3	2	3	2
86	2	1	5	3	5	1	3	1	3	1	2	2	1	1	1	3	2	2	2	1	1	1	1	1	1
87	2	2	5	3	5	2	5	2	3	3	3	2	1	3	2	3	3	2	3	2	2	2	2	3	1
88	4	2	5	3	5	2	5	2	3	3	3	3	2	3	2	3	2	3	5	2	1	3	3	3	2
89	2	3	5	3	5	3	5	2	3	2	3	3	2	3	2	3	3	2	3	2	2	3	3	3	3
90	3	5	5	3	5	2	5	3	3	3	3	3	1	3	3	3	3	2	3	2	2	3	3	3	2
91	2	1	5	3	5	2	5	1	3	2	2	2	1	1	2	1	2	2	2	2	1	2	2	2	1
92	2	1	5	3	5	1	5	1	3	2	3	3	1	3	2	3	3	3	2	2	1	1	2	1	1
93	2	5	5	3	5	3	5	2	3	3	3	3	1	3	2	3	2	2	2	2	1	2	2	3	2
94	2	1	5	3	5	3	5	1	3	3	2	1	1	1	1	3	3	2	2	2	1	1	2	1	1
95	2	2	5	3	5	2	5	1	3	2	2	3	1	1	1	1	3	2	2	2	2	2	2	2	1
96	2	3	5	3	5	1	5	1	3	2	3	3	1	1	2	2	3	2	2	2	2	3	2	3	2
97	4	3	5	3	5	3	5	3	3	3	3	3	2	3	3	3	3	2	3	3	2	3	3	3	3
98	2	3	5	3	5	2	3	1	3	3	2	1	1	3	2	3	2	2	2	2	1	1	2	1	1
99	3	5	5	3	5	3	5	3	3	3	3	3	2	3	2	3	3	2	3	2	2	3	3	3	3
100	3	5	5	3	5	2	5	3	3	3	3	3	2	3	3	3	3	3	5	3	3	3	3	3	3
101	2	3	5	3	5	1	3	1	3	2	2	2	1	3	1	3	3	2	3	2	2	1	2	3	1
102	2	1	5	3	5	3	5	1	3	3	2	2	1	3	2	3	3	2	3	2	1	2	2	3	2
103	2	1	5	3	5	3	5	1	3	3	2	2	2	3	1	1	3	2	2	2	2	3	2	2	2
104	4	5	5	3	5	3	5	3	3	3	3	3	2	3	3	3	3	3	5	3	2	3	3	3	3
105	4	3	5	3	5	3	5	2	3	3	3	3	2	3	3	3	3	2	3	2	2	3	2	3	2
106	2	5	5	3	5	2	5	3	3	3	3	3	2	3	3	3	3	3	3	3	1	3	3	3	2
107	2	5	5	3	5	2	5	3	5	3	3	3	3	3	3	3	3	3	3	3	3	3	3	3	3
108	3	3	5	3	5	2	5	1	3	3	3	3	3	3	2	3	3	2	3	2	3	3	2	3	3

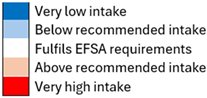

**Table A5:** Principal component analysis: variance explained by retained dietary patterns.

Component	Eigenvalue	Variance Explained (%)	Cumulative Variance (%)
1	3.190	15.2	15.2
2	2.386	11.4	26.6
3	1.992	9.5	36.0
4	1.442	6.9	42.9
5	1.321	6.3	49.2

Footnote: PCA was performed on food group intake variables. Five dietary patterns were retained based on scree plot inspection and component interpretability. The Kaiser–Meyer–Olkin (KMO) measure of sampling adequacy was 0.636, and Bartlett’s test of sphericity was significant (χ^2^ = 812.994, df = 231, *p* < 0.001). Extraction method: Principal Component Analysis.

**Table A6:** Rotated factor loadings for the five retained dietary patterns.

		Dietary	Pattern		
Food Group	Snack and Convenience	Plant-Based	Animal Protein	Traditional Staple	Health-Conscious
Savory snacks	0.786				
Sweets	0.709				
Bread	0.568				
Legumes		0.837			
Nuts and seeds		0.783			0.629
Grains		0.511			
Vegetables		0.471		0.459	0.453
Eggs			0.743		
Meat and poultry		−0.518	0.553		
Fish			0.485		
Fruit		0.416		0.384	0.385
Potatoes	0.317			0.729	
Cold cuts				0.367	
Cakes	0.334			−0.457	
Non-alcoholic beverages				−0.314	−0.439
Fats	0.360				0.648
Alcohol					−0.563
Cheese					−0.361
Savory sauces	0.449				
Ready meals	0.387				

Only factor loadings ≥ |0.30| are displayed. Dietary patterns were derived using principal component analysis (PCA) on food group intake variables. Five dietary patterns were retained based on inspection of the scree plot and component interpretability. Extraction method: Principal Component Analysis. Rotation method: Varimax with Kaiser normalization. Rotation converged in 32 iterations.

**Table A7:** Dietary inflammatory index (DII) at baseline and after 6 months.

Food Parameter	IES	Mean	SD	Intake T0	Intake T6	Z-Score T0	Z-Score T6	PR T0	PR T6	DII T0	DII T6
Alcohol total [g]	−0.278	14.0	3.72	3.13	3.00	−2.916	−2.951	0.002	0.002	0.277	0.277
Vitamin B12 [ug]	0.106	5.15	2.70	3.67	3.73	−0.546	−0.527	0.292	0.299	−0.044	−0.043
Vitamin B6 [mg]	−0.365	1.47	0.74	1.50	1.43	0.042	−0.053	0.517	0.479	−0.012	0.015
Beta-carotene [ug]	−0.584	3718	1720	3101	2915	−0.359	−0.467	0.360	0.320	0.164	0.210
Carbohydrates [g]	0.097	272	40.0	204	191	−1.712	−2.024	0.043	0.021	−0.089	−0.093
Cholesterol [mg]	0.110	279	51.2	227	223	−1.033	−1.107	0.151	0.134	−0.077	−0.080
Energy [kcal]	0.180	2056	338	2046	1924	−0.028	−0.391	0.489	0.348	−0.004	−0.055
Fat total [g]	0.298	71.4	19.4	96.0	89.2	1.267	0.917	0.897	0.820	0.237	0.191
Fiber total [g]	−0.663	18.8	4.90	26.2	24.7	1.506	1.202	0.934	0.885	−0.576	−0.511
Folate [ug]	−0.190	273	70.7	260	243	−0.181	−0.427	0.428	0.335	0.027	0.063
Iron total [mg]	0.032	13.4	3.71	10.4	9.83	−0.794	−0.948	0.214	0.171	−0.018	−0.021
Magnesium [mg]	−0.484	310	139	340	326	0.213	0.117	0.584	0.546	−0.082	−0.045
Fatty acids MUFA [g]	−0.009	27.0	6.10	41.2	38.0	2.331	1.807	0.990	0.965	−0.009	−0.008
Nicotinic Acid [mg]	−0.246	25.9	11.7	18.1	17.3	−0.664	−0.735	0.253	0.231	0.121	0.132
Fatty acids *n*-3 PUFA [g]	−0.436	1.06	1.06	2.85	2.67	1.687	1.515	0.954	0.935	−0.396	−0.379
Fatty acids *n*-6 PUFA [g]	−0.159	10.8	7.50	14.4	13.2	0.474	0.317	0.682	0.625	−0.058	−0.040
Protein total [g]	0.021	79.4	13.9	71.8	70.1	−0.550	−0.671	0.291	0.251	−0.009	−0.010
Fatty acids PUFA [g]	−0.337	13.9	3.76	18.3	16.8	1.163	0.788	0.878	0.785	−0.254	−0.192
Vitamin B2 [mg]	−0.068	1.70	0.79	1.13	1.12	−0.720	−0.736	0.236	0.231	0.036	0.037
Fatty acids saturated [g]	0.373	28.6	8.00	29.2	27.4	0.072	−0.155	0.529	0.438	0.021	−0.046
Selenium [ug]	−0.191	67.0	25.1	51.9	51.3	−0.603	−0.625	0.273	0.266	0.087	0.089
Vitamin B1 [mg]	−0.098	1.70	0.66	0.89	0.84	−1.228	−1.305	0.110	0.096	0.076	0.079
Fatty acids trans [g]	0.229	3.15	3.75	0.60	0.58	−0.679	−0.686	0.249	0.246	−0.115	−0.116
Retinol equivalents ([ug]	−0.401	984	519	854	811	−0.250	−0.334	0.401	0.369	0.079	0.105
Vitamin C [mg]	−0.424	118	43.5	93.0	84.2	−0.580	−0.782	0.281	0.217	0.186	0.240
Vitamin D total [ug]	−0.446	6.26	2.21	2.54	2.51	−1.682	−1.695	0.046	0.045	0.405	0.406
Vitamin E total [mg]	−0.419	8.73	1.49	14.5	13.4	3.885	3.147	1.000	0.999	−0.419	−0.418
Zinc [mg]	−0.313	9.84	2.19	9.56	9.29	−0.126	−0.251	0.450	0.401	0.031	0.062
								Mean DII		−0.414	−0.152

**Abbreviations:** IES, inflammatory eating score; SD, standard deviation; PR, percentile ratio; DII, Dietary inflammatory index; MUFA, mono-unsaturated fatty acids; PUFA, poly-unsaturated fatty acids. Mean represents the global mean intake in units/day; PR is calculated by multiplying the percentile scores and subtracting 1, which is all multiplied by IES: ([PS × 2] − 1) × IES. Then, DII per nutrient is calculated by 2 × PR × IES. (Shivappa et al. [16]).
