# Supplementary material for: Dietary Analyses and Personalized Advice in Patients with Inflammatory Bowel Disease: The DAPA Study
Source: Nutrients. 2026 Aug 11;18(16):2626. doi: 10.3390/nu18162626 (PMC13516606; doi:10.3390/nu18162626)
Supplement: Supplementary file 1 [file nutrients-18-02626-s001.zip › nutrients-4420788-supplement file/Supplementary File S1. Nutritional Knowledge Tool Nutrition and IBD (Dutch version)..pdf]

Voeding bij inflammatory bowel disease

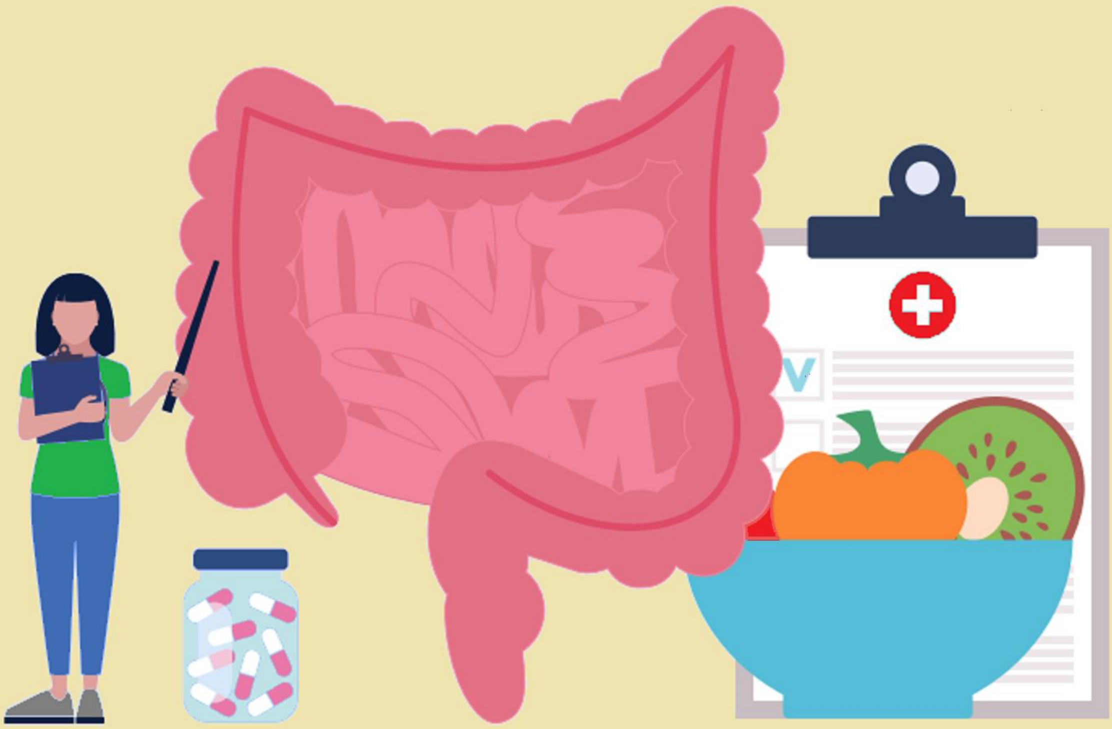

Kennisportaal

Inhoudsopgave

Inhoudsopgave 2

Wat is IBD? 3

Adviezen bij angst voor het eten van bepaalde producten 4

Wat is het microbioom en wat doet het? 5

Hoe kan ik voldoende voedingsvezels eten? 6

Hoe zorg ik dat ik voldoende eiwitten binnenkrijgt? 10

Hoe kan ik zorgen dat mijn voeding voldoende ijzer bevat? 15

Wat kan ik doen als melkproducten minder goed worden verdragen? 17

Welke soorten vet kan ik het beste eten als ik IBD heb? 18

Hoe krijg ik voldoende voedingsstoffen binnen als ik vegetarisch wil eten? 19

Welke voedingsmiddelen kunnen klachten van opgeblazen gevoel en winderigheid veroorzaken? 21

Moet ik gluten uit mijn voeding weglaten? 22

Welke voeding kan ik gebruiken om ontstekingen te voorkómen? 23

Wat kan ik doen bij diarree? 24

Wat is galzure diarree en wat kan ik eraan doen? 27

Wat kan ik doen bij verstopping? 28

Wat kan ik eten bij een vernauwing (stenose) van de darm? 29

Waarop moet ik bij mijn eten letten als ik een stoma heb? 30

- Algemene tips bij een stoma 30
- Voedingstips bij een colostoma (=stoma op de dikke darm) 31
- Voedingstips bij een ileostoma (=stoma op de dunne darm) 31

Zijn er speciale dingen waar ik bij mijn eten op moet letten als ik een pouch heb? 33

- Tabel. Producten die veel zout leveren. 34

Moet ik extra vitamine preparaten nemen? 37

Heeft het voordelen om probiotica te gebruiken bij de behandeling van IBD? 39

Heeft het voordelen om omega 3 supplementen of visolie te gebruiken? 40

Is het verstandig om kurkuma te gebruiken? 41

Wat is het FODMAP dieet en is het verstandig om dit te gebruiken? 42

Wat is het CDED dieet en is het geschikt voor mij? 43

Waar moet ik bij mijn voeding op letten als ik op vakantie ga? 44

Wanneer raadpleeg ik een diëtist? 45

Waar vind ik een diëtist die meer weet van voeding bij IBD? 45

Wat is IBD?

IBD staat voor "Inflammatoire Darmziekten." Dit zijn aandoeningen waarbij de darmen ontstoken raken. Er zijn twee veelvoorkomende vormen van IBD: de ziekte van Crohn en colitis ulcerosa. Mensen met IBD kunnen symptomen hebben zoals buikpijn, diarree, vermoeidheid en gewichtsverlies. De oorzaak van IBD is nog niet helemaal duidelijk, maar het heeft te maken met het immuunsysteem dat de darmen aanvalt. IBD is een chronische aandoening, wat betekent dat het langdurig is en soms opeens erger wordt, opvlamt. Behandelingen, zoals medicijnen en dieetveranderingen, kunnen helpen de symptomen onder controle te houden en de kwaliteit van leven te verbeteren.

De ziekte van Crohn en colitis ulcerosa zijn twee soorten inflammatoire darmziekten (IBD) met belangrijke verschillen:

Voorkomen en aard van ontsteking:
De ziekte van Crohn kan elk deel van het spijsverteringskanaal aantasten, van mond tot anus dus. Ook kan de ontsteking tot diep in de darmwand zitten, met afwisseling tussen gezonde en ontstoken delen. Doordat de ontsteking tot zo diep in de darmwand kan doordringen, kan deze zich ook uitbreiden naar omliggende weefsels. Dit kan littekenvorming en andere complicaties zoals fistels en abcessen veroorzaken.

Bij Colitis ulcerosa beperkt de ontsteking zich tot de dikke darm (colon) en het rectum, waarbij de ontsteking meestal begint in het rectum en zich geleidelijk naar andere delen van de dikke darm verspreidt. De ontsteking zelf, zit voornamelijk in de slijmvlieslaag, de bovenste laag, van de darmwand. Dit leidt tot oppervlakkige zweren en bloedingen in de dikke darm.

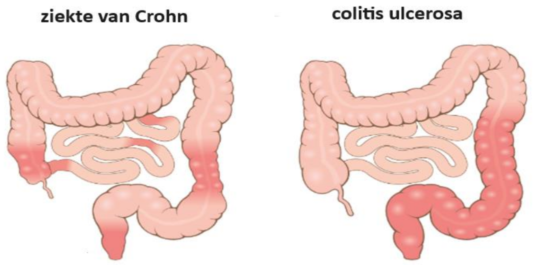

Symptomen:
Symptomen van de ziekte van Crohn variëren, maar zijn vaak buikpijn, diarree, gewichtsverlies, vermoeidheid en soms koorts. Colitis ulcerosa veroorzaakt meestal maag-darmproblemen zoals frequente diarree, buikpijn en bloederige ontlasting. Ook kunnen bij zowel de ziekte van Crohn als colitis ulcerosa andere lichaamsdelen zoals gewrichten en de huid betrokken raken bij de ontsteking. Dan spreken we van bijvoorbeeld artritis en erythema nodosum.

Complicaties:
Regelmatig voorkomende complicaties bij de ziekte van Crohn zijn onder andere fistels, abcessen, darmobstructie en littekenvorming. Bij colitis ulcerosa zien we vaker een ernstige vergroting van de dikke darm (megacolon) en systemische ontstekingsproblemen (= betrokkenheid van andere lichaamsdelen zoals gewrichten en huid). Bij beide ziektebeelden bestaat er een vergroot risico op het ontwikkelen van darmkanker.

Behandeling
Beide aandoeningen zijn chronisch en kunnen perioden van opvlammingen en remissie hebben. De behandeling is afhankelijk van de diagnose en kan bestaan uit medicijnen, dieetveranderingen, levensstijlaanpassingen en soms chirurgie. Het is belangrijk om samen te werken met een maag, darm, lever-arts voor het krijgen van een nauwkeurige diagnose en het opstellen van een geschikt behandelplan.

Adviezen bij angst voor het eten van bepaalde producten

Veel mensen met IBD willen graag weten wat ze kunnen eten om te voorkómen dat de ziekte opvlamt. Voeding heeft namelijk direct contact met je darmen. Daarom stellen veel patiënten aan hun dokter of diëtist de vraag "Wat kan ik eten?". Op dit moment is er nog geen duidelijk antwoord op deze vraag, omdat er nog geen definitieve conclusies zijn getrokken uit voedingsonderzoek. Omdat dit antwoord ontbreekt, gaan veel mensen met IBD zelf experimenteren met hun voeding op basis van hun klachten. Het weglaten van bepaalde producten uit hun dieet, kan echter snel leiden tot tekorten aan voedingsstoffen.

Ondanks dat er nog geen definitieve conclusies zijn uit eerder voedingsonderzoek, betekent dat niet dat we helemaal niets weten over wat goed is om te eten en wat niet. Daarom hebben we een aantal voedingstips voor je verzameld. Voedingsonderzoek laat zien dat mensen met IBD vaak melk en melkproducten weglaten, wat leidt tot een lage inname van calcium. Dat is een probleem, want zij hebben vaak juist een verhoogd risico op botontkalking en calcium is belangrijk in het onderhoud van je botkwaliteit. Verderop in dit kennisportaal geven we je tips over hoe je hiermee om kunt gaan. (zie: *Wat kan ik doen als melkproducten minder goed worden verdragen?*)

Bovendien zijn veel mensen met IBD vaak bezorgd om bepaalde soorten groenten en fruit te eten. Dit kan soms leiden tot een opgeblazen gevoel, winderigheid of buikpijn. Toch toont onderzoek aan dat het eten van voldoende plantaardige vezels, die vooral in groenten en fruit voorkomen maar ook in aardappelen en volkoren graanproducten, juist goed is voor de darmen. Dit komt doordat vezels een voedingsbodem zijn voor gezonde darmbacteriën. Nadat in de maag en dunne darm de meeste voedingsstoffen uit het voedsel zijn opgenomen, bereiken de voedingsvezels de dikke darm. Door het verhogen van de inname van voedingsvezels verbeter je dus de samenstelling van de darmbacteriën, wat resulteert in een gezonder microbioom (zie: *Wat is het microbioom en wat doet het?*) Onderzoek laat ook zien dat mensen die vooral vegetarisch eten, wat vaak gepaard gaat met een verhoogde consumptie van meer voedingsvezels, een gezonder microbioom hebben (Chiba, 2010). Raadpleeg voor meer informatie: *Hoe kan ik voldoende voedingsvezels eten?*

In dit kennisportaal vind je ook adviezen voor klachten als winderigheid, opgeblazen gevoel, (zie: *Welke voedingsmiddelen kunnen klachten van opgeblazen gevoel en winderigheid veroorzaken?*) verstopping (zie: *Wat kan ik doen bij verstopping?*) of diarree (zie: *Wat kan ik doen bij diarree?*). Voor veel van deze klachten kan het proberen van een FODMAP-beperkt dieet nuttig zijn, maar hiervoor is begeleiding van een diëtist noodzakelijk. Als je bepaalde voedingsmiddelen weglaat uit angst om deze te eten, kan het ook verstandig zijn om een diëtist te raadplegen.

Referentie

Chiba M, Abe T, Tsuda H, Sugawara T, Tsuda S, Tozawa H, Fujiwara K, Imai H. Lifestyle-related disease in Crohn's disease: relapse prevention by a semi-vegetarian diet. World J Gastroenterol. 2010 May 28;16(20):2484-95. doi: 10.3748/wjg.v16.i20.2484. PMID: 20503448; PMCID: PMC2877178.

Wat is het microbioom en wat doet het?

In onze darmen zitten veel kleine organismen, zoals bacteriën, gisten en virussen. Samen noemen we ze het darmmicrobioom. Het microbioom doet belangrijke dingen om ons te beschermen tegen ziektes. Daarnaast maakt het ook enkele vitamines aan, helpt met het verwerken van voedingsvezels en zorgt het voor de gezondheid van onze darmwand.

De samenstelling van je microbioom kan veranderen als je genoeg beweegt, goed slaapt en genoeg ontspant. Maar eten is de grootste factor die je microbioom beïnvloedt. De darmbacteriën leven van wat er overblijft in je dikke darm na het eten. Ze houden vooral van vezels, die zitten in groenten, fruit, peulvruchten en volkoren producten. Als je hiervan veel en gevarieerd eet, kunnen de goede darmbacteriën groeien en krijg je veel verschillende soorten bacteriën in je darmen, die samen proberen je darmen gezond te houden. Als je veel zoete, vette en bewerkte producten zoals koekjes, chips, koekjes en sauzen eet, geef je juist de "slechte" darmbacteriën te eten. Deze “slechte” darmbacteriën kunnen juist ontsteking in de darm in de hand werken.

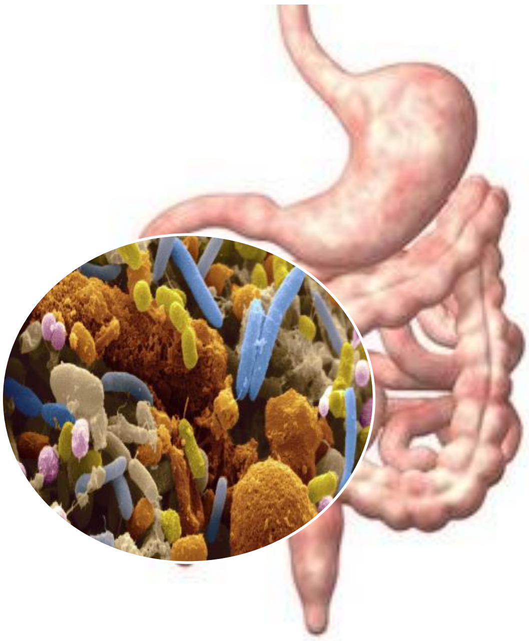

Hoe kan ik voldoende voedingsvezels eten?

Voor een gezond darmmicrobioom is het belangrijk om voldoende voedingsvezels te eten. Voedingsvezels zitten alleen in plantaardige voedingsmiddelen. In veel bewerkte voedingsmiddelen zoals witbrood, beschuit en macaroni zijn deze vezels verwijderd. Voorbeelden van voedingsmiddelen met veel vezels zijn volkorenbrood, fruit, noten en peulvruchten (zoals bonen, linzen, kikkererwten).

Er zijn twee soorten vezels:

- **Oplosbare voedingsvezels:** deze stimuleren samen met bacteriën de beweging van de darm en zorgen ervoor dat de ontlasting soepel en niet hard blijft. Je vindt deze vezels in groente, fruit en peulvruchten.
- **Onoplosbare voedingsvezels**: deze houden de ontlasting soepel en zacht doordat ze water opnemen en vasthouden. Ze helpen bij verstopping en darmkrampen. Je kunt ze vinden in volkorenproducten (zoals volkorenbrood, havermout en zilvervliesrijst), noten, pinda's en zaden.

Tip: Als je niet gewend bent om veel vezels te gebruiken, kan dit in het begin leiden tot een opgeblazen gevoel en meer winderigheid. In dat geval is het belangrijk om de hoeveelheid voedingsvezels langzaam op te bouwen, deze goed over de dag te verdelen en genoeg te drinken. Je kunt bijvoorbeeld elke dag één vezelrijk product aan je dieet toevoegen. Na verloop van tijd zullen deze klachten verminderen en raakt je lichaam gewend aan een gezondere hoeveelheid vezels.

Mensen met IBD eten gemiddeld tussen de 9,9 en 21,0 gram vezels per dag[1]. Volwassen vrouwen wordt geadviseerd om dagelijks 30 gram vezels te eten terwijl volwassen mannen 40 gram per dag zouden moeten binnenkrijgen.

Je kunt de hoeveelheid vezels in je voeding verhogen door :

- Wit- of bruinbrood te vervangen door volkorenbrood
- Witte crackers en beschuit te vervangen door volkoren crackers en beschuit
- Witte pasta te vervangen door volkorenpasta
- Witte rijst te vervangen door zilvervliesrijst
- Couscous te vervangen door volkoren couscous
- Krokante muesli of ontbijtgranen of te vervangen door muesli, havermout of lijnzaad
- Extra noten of zaden aan een recept toe te voegen
- Extra peulvruchten door een salade te doen
- Vaker groente of fruit bij een maaltijd of tussendoor te eten

Hier is de tabel die laat zien welke voedingsmiddelen veel en in welke weinig voedingsvezels bevatten:

Tabel met voedingsvezels

**Voedingsmiddel**	**Eenheid**	**gram**	**Vezel gram**
**Brood en graanproducten**			
Beschuit	1 stuks	10	0
Beschuit, volkoren	1 stuks	10	1
Broodje, volkoren	1 stuks	50	3,4
Broodje, wit	1 stuks	50	1
Bruinbrood	1 snee	35	1,8
Cracker	1 stuks	10	0
Cracker, volkoren	1 stuks	10	1
Croissant	1 stuks	40	1
Evergreen	1 stuks	20	1
Knäckebröd	1 snee	10	1
Knäckebröd, vezelrijk	1 snee	10	2
Krentenbol	1 stuks	50	2
Krentenbrood	1 snee	35	1
Krentenbrood, volkoren	1 snee	35	2
Ontbijtkoek	1 plak	20	1
Ontbijtkoek, volkoren	1 plak	20	1
Rijstwafel	1 stuks	7	0
Roggebrood, donker	1 snee	50	3
Roggebrood, licht	1 snee	25	2
Volkorenbrood	1 snee	35	2,4
Wittebrood	1 snee	35	1
**Ontbijtproducten**			
Brinta	4 eetlepels	25	2
Cornflakes	6 eetlepels	25	1
Havermout	5 eetlepels	25	2

Voedingsmiddel	Eenheid	gram	Vezel gram
Muesli	3 eetlepels	25	2
Krokante muesli	3 eetlepels	25	2
Rice krispies	5 eetlepels	25	1
Weetabix cereal	1 stuks	20	2
**Vezelproducten**			
All Bran	5 eetlepels	25	7
Chiazaad	2 eetlepels	10	6
Farmafiber	1 zakje	5	4
Fibrex	3 eetlepels	25	18
Lijnzaad*	1 eetlepel	5	1
Tarwekiemen	4 eetlepels	25	8
Vezelreep	1 stuks	25	4
Zemelen	5 eetlepels	25	11
**Aardappelen en vervangers**			
Aardappelen	1 stuks	50	2
Macaroni, spaghetti, gekookt	1 grote lepel	50	0
Macaroni, spaghetti, volkoren gekookt	1 grote lepel	50	2
Rijst, gekookt	1 grote lepel	50	0
Rijst, zilvervlies, gekookt	1 grote lepel	50	1
Rijst, meergranen, gekookt	1 grote lepel	50	1,5
Couscous, bereid	1 grote lepel	50	0,5
Volkoren couscous, bereid	1 grote lepel	50	1,4
Bulgur, bereid	1 grote lepel	50	2,5
**Peulvruchten**			
Bruine, witte bonen, kapucijners, kikkererwten, linzen, etc	1 grote lepel	50	6
Erwtensoep	1 bord	250	10
**Groente**			
Groente, gekookt	1 grote lepel	50	1
Groente, rauw	1 schaaltje	50	1

**Voedingsmiddel**	**Eenheid**	**gram**	**Vezel gram**
**Fruit**			
1 appel	1 stuks	120	3
Bessen	1 schaaltje	100	7
Fruit, gemiddeld, geen citrus	1 stuks	120	4
Fruit, citrus, gemiddeld (sinaasappel, grapefruit, mandarijn)	1 stuks	120	2
Gedroogde zuidvruchten, geweekt (abrikozen, pruimen, tuttifrutti)	1 schaaltje	125	8
Dadels gekonfijt	10 stuks	60	5
Krenten, rozijnen	1 eetlepel	10	1
Vijgen gedroogd	3 stuks	60	11
Vruchtensap	1 glas	100	0
Vruchtensap met vruchtvlees	1 glas	100	1
**Diversen**			
Hummus	1 eetlepel	20	2
Pindakaas	1 eetlepel	20	2
Kikkererwten, geroosterd	1 eetlepel	20	4
Mueslibar	1 stuks	25	1
Mueslikoek	1 grote	50	3
Noten, pinda’s	1 eetlepel	20	2
Popcorn	1 zakje	25	1
Sesamzaad	1 eetlepel	12	0
Studentenhaver	1 eetlepel	20	2
Volkoren biscuitje	1 stuks	10	1
Pompoenpitten	1 eetlepel	15	1

*Gebruik niet meer dan 2-3 eetlepels lijnzaad per dag

Referentie:

1.Day AS, Davis R, Costello SP, Yao CK, Andrews JM, Bryant RV. The Adequacy of Habitual Dietary Fiber Intake in Individuals With Inflammatory Bowel Disease: A Systematic Review. J Acad Nutr Diet. 2021 Apr;121(4):688-708.e3. doi: 10.1016/j.jand.2020.12.001. Epub 2021 Jan 20. PMID: 33485803.

Hoe zorg ik dat ik voldoende eiwitten binnenkrijgt?

Eiwitten zijn essentieel voor het functioneren van je lichaam en hebben verschillende belangrijke functies:

1. **Bouwstof:** Eiwitten zijn de bouwstenen van je lichaam. Ze spelen een heel belangrijke rol bij de groei, het onderhoud en de reparatie van weefsels, zoals spieren, huid, haar en organen.
2. **Spierbehoud en -groei:** Eiwitten zijn van vitaal belang voor het behoud en de groei van spiermassa. Ze voorzien de spieren van de nodige aminozuren om te functioneren en te groeien.
3. **Organen en enzymen:** Eiwitten zijn betrokken bij de werking van enzymen, die chemische reacties in je lichaam versnellen. Ze zijn ook belangrijk voor de structuur en functie van organen.
4. **Immuunsysteem:** Eiwitten spelen een rol bij het functioneren van het immuunsysteem, waardoor je lichaam zich kan verdedigen tegen ziekteverwekkers.
5. **Transport van stoffen:** Eiwitten in de bloedbaan transporteren voedingsstoffen, hormonen en andere moleculen naar de cellen. Zo transporteert bijvoorbeeld het eiwit hemoglobine zuurstof vanuit de longen naar alle weefsels en organen in je lichaam.

Het is van belang om voldoende eiwitten binnen te krijgen om al deze functies te ondersteunen, vooral als je een ontstekingsziekte hebt zoals IBD, waarbij het risico op eiwittekorten kan toenemen.

Om ervoor te zorgen dat je voldoende eiwitten binnenkrijgt, zijn hier enkele richtlijnen:

1. **Eet eiwitrijke voedingsmiddelen:** Zorg ervoor dat je regelmatig eiwitrijke voedingsmiddelen opneemt in je dieet. Deze kunnen zowel dierlijk als plantaardig zijn.
2. **Dierlijke eiwitten** zitten vooral in**:**
   - Vis;
   - Vlees;
   - Gevogelte, zoals kip;
   - Kaas en melk(producten);
   - Eieren.
3. **Plantaardige** eiwitten zitten vooral in:
   - Peulvruchten zoals kikkererwten, bruine bonen, kidneybonen, sojabonen en linzen;
   - Tofu en tempé, sojamelk;
   - Granenproducten zoals volkorenbrood, volkorenpasta en couscous;
   - Noten;
   - Quorn (een eiwitrijke gefermenteerde schimmel).

Eiwitten uit dierlijke producten worden vaak "complete eiwitten" genoemd omdat ze alle essentiële bouwstenen (ook wel aminozuren genoemd) in de juiste verhoudingen bevatten. Essentiële aminozuren zijn die aminozuren die het lichaam niet zelf kan produceren en dus via voeding moeten worden verkregen.

In eiwitten uit plantaardige producten zitten ook essentiële aminozuren, maar ze bevatten meestal niet alle aminozuren in de ideale verhoudingen. Dit betekent dat plantaardige eiwitten vaak als "onvolledige eiwitten" worden beschouwd. Om ervoor te zorgen dat je alle benodigde aminozuren binnenkrijgt, is het inderdaad nuttig om verschillende plantaardige voedingsmiddelen te combineren. Bijvoorbeeld, het combineren van granen (zoals tarwe of rijst) met peulvruchten (zoals bonen of linzen) in dezelfde maaltijd kan resulteren in een evenwichtige mix van aminozuren.

Soja is een uitzondering onder de plantaardige eiwitbronnen, omdat het van nature een compleet eiwit is, wat betekent dat het alle essentiële aminozuren bevat in de juiste verhoudingen. Daarom is soja een waardevolle plantaardige bron van eiwitten voor mensen die geen dierlijke producten eten of verlangen naar een plantaardig dieet.

Het is belangrijk om te onthouden dat voedingsbehoeften kunnen variëren, vooral tijdens periodes van actieve ontsteking of ziekte. Dan heeft het lichaam vaak meer eiwitten nodig voor herstel. Daarom is overleg met een medische professional die bekend is met je situatie cruciaal om ervoor te zorgen dat je de juiste hoeveelheid eiwitten binnenkrijgt om je gezondheid te ondersteunen.

Hier zijn enkele handige **tips om meer eiwitten binnen te krijgen**:

1. **Eiwitten verspreiden over de dag:** Zorg ervoor dat je eiwitten gelijkmatig verdeelt over de dag. Terwijl de avondmaaltijd vaak al veel eiwitten bevat door vlees of vleesvervangers, kunnen het ontbijt en de lunch extra aandacht nodig hebben. Je kunt bijvoorbeeld melkproducten, hartig beleg of eieren aan deze maaltijden toevoegen om je eiwitinname te verbeteren.

2. **Kwark, Skyr, Griekse yoghurt:** Deze zuivelproducten bevatten meer eiwitten dan gewone melk of yoghurt. Ze kunnen een geweldige aanvulling zijn op je dieet om je eiwitinname te verhogen.

3. **Eiwitten na inspanning en voor het slapen:** Het consumeren van eiwitten na lichamelijke inspanning en voor het slapengaan kan de spiergroei en het spierherstel bevorderen. Overweeg een bakje kwark, een eiwitrijke smoothie of een eiwitshake na het sporten of voor het slapen.

4. **Eiwitshakes of dieetpreparaten:** Als je moeite hebt om voldoende eiwitten uit voedselbronnen te halen, kunnen eiwitshakes of dieetpreparaten nuttig zijn. Een diëtist kan je begeleiden bij het kiezen van de juiste producten en de juiste hoeveelheden om aan je behoeften te voldoen.

Het is belangrijk om te onthouden dat sommige mensen met IBD moeite kunnen hebben met bepaalde voedingsmiddelen vanwege problemen zoals lactose-intolerantie, gasvorming of een gevoeligheid voor vezelrijk voedsel. Het is heel belangrijk om een evenwichtig dieet te behouden en niet te veel producten uit je voedingspatroon weg te laten, omdat dit kan leiden tot voedingstekorten. **Raadpleeg een diëtist** voor gepersonaliseerd advies dat rekening houdt met je specifieke behoeften en beperkingen.

Eiwitinname in de praktijk:
Uit onderzoek blijkt dat veel patiënten met IBD onvoldoende eiwitten binnenkrijgen, ongeacht of zij in een rustige of actieve fase van hun ziekte zitten. In de **rustige fase** van de ziekte hebben mensen met IBD 0,83 gram eiwit per kg lichaamsgewicht nodig. 39% van de mensen met IBD haalt dit niet. Tijdens de **actieve fase** van je ziekte heb je minimaal 1,2 gram eiwit per kg lichaamsgewicht nodig. 87% van de mensen met actieve IBD haalt dit niet (Peters V, 2021). Voor mensen die meer willen rekenen of hun eiwitinname voldoende is, hebben we de eiwitbehoefte aangegeven en een eiwittabel toegevoegd. Als je dit te ingewikkeld vindt, kun je ook een diëtist raadplegen.

Eiwittabel
Een gemiddelde volwassen persoon heeft 0,83 gram eiwit per kg lichaamsgewicht nodig. Dit is bij 70 kg dus 0,83 x 70 = 58,1 gram eiwit. Bij 80 kg is dit 66,4 gram eiwit. Bij actieve ziekte stijgt deze behoefte naar minimaal 1,2 gram per kg lichaamsgewicht. Dit betekent dat de persoon met actieve ziekte van 70 kg ten minste 84 gram eiwit nodig heeft; de persoon van 80 kg heeft 96 gram eiwit per dag nodig. Om je een indruk te geven hoeveel eiwit er in verschillende producten in zit, vind je hier een eiwittabel met veel voorkomende eiwitrijke producten:

**Product**	**Eenheid**	**gram**	**Eiwit (gram)**
**Vlees**			
Kip of kalkoen	1 stuk	100	27
Rundvlees (gem)	1 stuk	100	29
Varkensvlees (gem)	1 stuk	100	20
Gehaktbal half om half	1 bal	95	19
Kipnuggets	3 stuks	60	11
**Vis**			
Vette vis (gem)	1 stuk	100	18
Magere vis (gem)	1 stuk	100	19
Kibbeling	1 bak	145	33
Vissticks	3 stuks	3x30	12
Haring	1 stuk	75	13
**Vleesvervangers**			
Tofu/tahoe	1 stuk	100	12
Tempé	1 stuk	100	12
Quorn	1 stuk	100	14
Eieren	2 stuks	100	14
Groentenburger	1 stuks	75	12
Peulvruchten	1 opscheplepel	60	5

Product	Eenheid	gram	Eiwit (gram)
**Melkproducten**			
Skyr	1 schaaltje	150	17
Hangop	1 schaaltje	150	15
Magere kwark	1 schaaltje	150	13
Halfvolle kwark	1 schaaltje	150	11
Volle kwark	1 schaaltje	150	11
Griekse yoghurt	1 schaaltje	150	7
Magere yoghurt	1 schaaltje	150	6
Halfvolle yoghurt	1 schaaltje	150	6
Volle yoghurt	1 schaaltje	150	6
Vla	1 schaaltje	150	5
Griesmeelpap	1 schaaltje	150	5
Magere melk	1 beker	150	5
Halfvolle melk	1 beker	150	5
Volle melk	1 beker	150	5
Chocolademelk	1 beker	150	5
Drinkyoghurt	1 beker	150	5
Karnemelk	1 beker	150	5
Milkshake	1 beker	200	11
Sojakwark	1 schaaltje	150	9
**Broodbeleg**			
Magere vleeswaren	1 plak	15	3
Worstsoorten	1 plak	15	2
Kaas 30+	1 plak	30	9
Kaas, 48+	1 plak	30	7
Ei	1 stuks	50	7
Pindakaas, 100% pinda	Voor 1 boterham	20	5
Zalm, gerookt	Voor 1 boterham	40	9
Makreel, gerookt	Voor 1 boterham	40	9

**Product**	**Eenheid**	**gram**	**Eiwit (gram)**
**Diversen**			
Erwtensoep met spek en worst	soepkom	250	16
Tosti	1 stuks	95	12
Kroket	1 stuks	65	6
Kaasblokje	3 blokjes	20	5
Ham rolletje met roomkaas	3 stuks	20	5
Noten, gemiddeld	1 handje	25	5
Zaden	1 eetlepel	15	5

Referentie

Peters V, Tigchelaar-Feenstra EF, Imhann F, Dekens JAM, Swertz MA, Franke LH, Wijmenga C, Weersma RK, Alizadeh BZ, Dijkstra G, Campmans-Kuijpers MJE. Habitual dietary intake of IBD patients differs from population controls: a case-control study. Eur J Nutr. 2021 Feb;60(1):345-356. doi: 10.1007/s00394-020-02250-z. Epub 2020 Apr 24. PMID: 32333097; PMCID: PMC7867519.

Hoe kan ik zorgen dat mijn voeding voldoende ijzer bevat?

Bij 19% van de mensen met de ziekte van Crohn of colitis ulcerosa komt een tekort aan ijzer voor.[1] Dit tekort kan vermoeidheid veroorzaken, wat vaak al een symptoom is van de ziekte zelf. Het is belangrijk om te zorgen dat je genoeg ijzer hebt om je energieniveau op peil te houden.

IJzer is een belangrijk voor je lichaam omdat het deel uitmaakt van hemoglobine, dat weer een deel is van je rode bloedcellen. Rode bloedcellen vervoeren zuurstof door je lichaam. Als je te weinig ijzer hebt, kun je je dus extra moe voelen, omdat zuurstof minder goed naar je organen en weefsels vervoerd kan worden. Alle organen en weefsels hebben zuurstof nodig om goed te kunnen werken.

Er zijn twee soorten ijzer in ons eten: heemijzer en non-heemijzer. **Heemijzer,** dat alleen in dierlijke producten voorkomt, wordt beter opgenomen in het lichaam en zit in voedingsmiddelen zoals vlees en vis. **Non-heemijzer** is te vinden in zowel dierlijke als plantaardige producten, zoals brood, peulvruchten, donkergroene groente (zoals spinazie), eieren en vleesvervangers. Hoewel non-heemijzer minder efficiënt wordt opgenomen in de darmen, vormt het toch het grootste deel (90%) van onze dagelijkse ijzerinname.

IJzerrijke producten
Dierlijke producten die ijzer bevatten zijn vlees, kip en vis. In rund- of lamsvlees zit meer ijzer dan in varkensvlees of kip.

Voorbeelden van **plantaardige producten** die ijzer bevatten zijn[2]:

- Aardappelen;
- Brood zoals volkorenbrood en bruinbrood, Vikron brood, vezelrijke knäckebröd, knäckebröd volkoren of goudbruin, luchtige cracottes, cracker natural, en volkoren beschuit;
- Fruit zoals, gedroogde abrikozen, rozijnen, krenten en vijgen;
- Graanproducten zoals haver, havermout, brinta, mueslireep, volkorenpasta en zilvervliesrijst;
- Groente zoals zongedroogde tomaat, snijbiet, rucola, spinazie en doperwten;
- Noten en zaden zoals maanzaad, pompoenpitten, hennepzaad, sesamzaad, cashewnoten, lijnzaad, pijnboompitten, zonnebloempitten en pistachenoten;
- Peulvruchten zoals groene en bruine linzen, kidneybonen, bruine en witte bonen en kapucijners. Maar ook sojabonen en producten die hiervan gemaakt zijn, zoals tofu, tempé en sojabrokjes zijn goede bronnen van ijzer.

Vitamine C verbetert de opname van non-heemijzer

Vitamine C helpt bij de opname van non-heemijzer. Daardoor is het belangrijk om bij een maaltijd iets van vitamine C te eten. Fruit bij een maaltijd zorgt er bijvoorbeeld voor dat ijzer uit brood gemakkelijker wordt opgenomen. Vitamine C zit in:

- Fruit zoals citrusfruit, kiwi's, bessen en aardbeien;
- Groente , vooral koolsoorten;
- Aardappelen.

Stoffen in voeding die de opname van ijzer tegenwerken:

Er zijn echter ook stoffen in voedsel die de opname van ijzer verminderen, zoals calcium (vooral in zuivelproducten), fytaat in granen en peulvruchten, en fenolen in thee en koffie. Drink daarom liever koffie, thee en zuivel liever niet bij een maaltijd die ijzer bevat, maar liever als tussendoortje.

Bij vegetariërs komt een ijzertekort vaker voor dan bij mensen die vlees eten omdat vlees een goede bron is van makkelijk opneembaar ijzer is. Daarom is het belangrijk voor vegetariërs om vleesvervangers te kiezen die voldoende ijzer bevatten, zoals vis, ei, peulvruchten, tofu, en tempé.

Samengevat kun je een ijzertekort voorkomen door:

- Eet regelmatig vlees of kies vleesvervangers die ijzer bevatten zoals vis, ei, peulvruchten, tofu en tempé.
- Eet voedingsmiddelen die rijk zijn aan ijzer, zoals volkorenbrood, volkorenpasta en peulvruchten.
- Voeg bij elke maaltijd groenten of fruit toe die rijk zijn aan vitamine C om de ijzeropname te verbeteren.

Referenties:

1. Vagianos, et al 2016. *Journal of Parenteral and Enteral Nutrition* 40 (3): 405–11
2. Nederlands Voedingsstoffenbestand (NEVO) | RIVM. (n.d.). https://nevo-online.rivm.nl/

Wat kan ik doen als melkproducten minder goed worden verdragen?

Zuivelproducten bevatten lactose, ook wel melksuiker genoemd. Sommige mensen kunnen deze melksuiker niet goed verteren. Toch is het belangrijk om zuivelproducten niet volledig weg te laten uit je voeding, omdat zuivel belangrijke voedingsstoffen, zoals calcium, bevat. Calcium is essentieel om je botten sterk te houden.

Er zijn enkele opties voor mensen die moeite hebben met lactose:

1. **Hollandse kaas:** In Hollandse kazen is de melksuiker (lactose) volledig verdwenen tijdens het kaasmaken. Daarom worden kazen vaak goed verdragen en zijn ze een goede bron van calcium.
2. **Zure melkproducten:** Producten zoals kwark, yoghurt en kefir bevatten minder lactose omdat een deel ervan tijdens de productie is weggelekt met de wei of is gefermenteerd. Deze producten worden meestal beter verdragen dan gewone melk of vla, waar alle lactose nog in zit.
3. **Vermijd bewerkte melkproducten:** Kant-en-klare bewerkte melkproducten zoals chocolademelk, yoghurtdrank en vla bevatten naast lactose ook toegevoegde suikers en hulpstoffen die niet ideaal zijn voor je darmgezondheid. Het is een goed idee om deze producten te beperken.
4. **Alternatieve melkproducten:** Je kunt melk vervangen door alternatieve producten zoals sojamelk waar calcium aan is toegevoegd. Tofu is ook een goede bron van calcium; 100 gram tofu bevat een aanzienlijke hoeveelheid calcium (200 mg).

Om voldoende calcium binnen te krijgen, is het belangrijk om dagelijks voldoende zuivel of alternatieven te consumeren. Je kunt bijvoorbeeld elke dag 4 porties zuivel eten, zoals 2 plakken kaas, 1 schaaltje kwark of yoghurt en 1 glas melk of karnemelk. Het is aan te raden om te experimenteren en te ontdekken welke zuivelproducten je het beste verdraagt. Zo kun je zorgen dat je voldoende calcium binnenkrijgt zonder dat je last krijgt.

Welke soorten vet kan ik het beste eten als ik IBD heb?

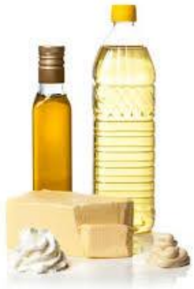

Onderzoek naar de samenstelling van vetten in de voeding tonen aan dat sommige vetten in verband kunnen worden gebracht met het risico op het ontwikkelen van ontstekingsziekten in de darm[1]. Goede vetten zoals omega-3 vetzuren, die vooral in vette vis zitten, worden dan weer in verband gebracht met een lager risico op colitis ulcerosa. Voor de ziekte van Crohn is dit verband nog niet gelegd.

Het eten van veel verzadigd vet wordt in verband gebracht met een verhoogd risico op colitis ulcerosa. Verzadigd vet zit in vet vlees, volle melkproducten, koek en snacks. Ook een hogere consumptie van omega-6 vetzuren zoals linolzuur leidt tot een verhoogd risico op colitis ulcerosa[2]. Linolzuur zit in plantaardige oliën zoals zonnebloemolie, maïsolie en sojaolie, maar ook in margarine, halvarine en bak- en braadvetten. Je kunt ze dus wel gebruiken, maar gebruik liever méér omega-3 vetzuren.

Gebruik dus vooral een voeding rijk aan omega-3-vetzuren en laag in omega-6 vetzuren.

Zie voor meer informatie over een goede samenstelling van vetten in je voeding: [Vetten | Voedingscentrum]

Referenties:

1. Ananthakrishnan AN, , *et al* Long-term intake of dietary fat and risk of ulcerative colitis and Crohn's disease. *Gut* 2014;**63:**776-784.
2. Tjonneland, A., Olsen, A., Overvad, K., Bergmann, M. M., Boeing, H., Nagel, G., . & Danielsson, A. (2009). IBD in EPIC Study Investigators: Linoleic acid, a dietary n-6 polyunsaturated fatty acid, and the aetiology of ulcerative colitis: a nested case-control study within a European prospective cohort study. Gut, 58, 1606-1611.

Hoe krijg ik voldoende voedingsstoffen binnen als ik vegetarisch wil eten?

Veel mensen kiezen ervoor om minder of helemaal geen vlees te eten om verschillende redenen zoals dierenwelzijn, milieu en/of hun gezondheid. Vegetariërs eten geen vlees, maar gebruiken meestal wel eieren en zuivel, wat lacto-ovo-vegetarisme wordt genoemd. Sommige vegetariërs eten vis en worden pescotariërs genoemd. Flexitariërs eten op sommige dagen geen vlees en op andere dagen weer wel. Veganisten vermijden alle dierlijke producten, inclusief melk en eieren.

Aangezien vlees een belangrijke bron is van eiwitten, ijzer, vitamine B1 en B12, is het mogelijk om vlees te vermijden, maar moet je er wel voor zorgen dat je voldoende eiwitten, ijzer en deze vitamines binnenkrijgt.

Hier zijn enkele tips om ervoor te zorgen dat je voldoende voedingsstoffen binnenkrijgt als vegetariër:

1. **Vitamine B12:** Vitamine B12 komt alleen voor in dierlijke producten. Als je iets van dierlijke producten zoals melk of ei eet krijg je voldoende vitamine B12 binnen. Maar als je helemaal geen vlees, vis, melk of eieren eet, wordt aanbevolen om een vitamine B12-supplement te gebruiken of voedingsmiddelen te consumeren die zijn verrijkt met vitamine B12 om ervoor te zorgen dat je voldoende binnenkrijgt.
2. **IJzer:** IJzer is belangrijk voor de vorming van rode bloedcellen en een tekort kan leiden tot bloedarmoede. IJzer komt in twee vormen voor in voedsel: heemijzer (goed opgenomen) in dierlijke producten en non-heemijzer (minder goed opgenomen) in zowel dierlijke als plantaardige producten. Non-heemijzer is te vinden in volkorenbrood, volkoren graanproducten, peulvruchten, sojaproducten (zoals tahoe, tempé en sojabrokjes), groene bladgroenten (zoals spinazie, postelein, paksoi), radijs, eieren en noten. Om de opname van non-heemijzer in de darmen te verbeteren, is het belangrijk om bij elke maaltijd groenten of fruit te eten, omdat deze vitamine C bevatten, wat de opname van dit type ijzer bevordert. (zie: *Hoe kan ik zorgen dat mijn voeding voldoende ijzer bevat?*)
3. **Vitamine B1:** Vitamine B1-tekort komt zelden voor bij vegetariërs, omdat vitamine B1 ook in brood, graanproducten, aardappelen, groenten en zuivel voorkomt.
4. **Eiwitten:** De meeste mensen krijgen voldoende eiwitten binnen, zelfs als ze minder of geen vlees eten. Normaal heb je genoeg aan ongeveer 0,8 gram eiwit per kilogram lichaamsgewicht. Vegetariërs hebben ongeveer 1 gram eiwit per kilogram lichaamsgewicht nodig, terwijl veganisten 1,2 gram per kilogram nodig hebben. Het is belangrijk om peulvruchten zoals bonen altijd te combineren met volkoren graanproducten, omdat bonen wat minder essentiële aminozuren bevatten. Goede combinaties zijn bijvoorbeeld bonen en zilvervliesrijst, volkorenbrood met hummus of erwtensoep met roggebrood. (zie: *Hoe zorg ik dat ik voldoende eiwitten binnenkrijgt?*)

5. **Alternatieven voor vlees:** Als vervanging voor 100 gram vlees kun je overwegen om 2 eieren te eten, 100 gram vis, 100 gram Quorn, 135 gram gekookte peulvruchten (zoals bruine bonen, kikkererwten, kidneybonen of linzen), 150 gram tofu, 150 gram tempé, 30 gram sojabrokjes of een handvol noten (25 gram).
6. **Kant-en-klare vleesvervangers:** Er zijn tegenwoordig veel vleesvervangers op de markt. Een goede vleesvervanger bevat minstens 14 gram eiwit, meer dan 0,8 mg ijzer per 100 gram en voldoende vitamine B1 (meer dan 0,06 mg per 100 gram). Let op dat vleesvervangers niet te veel verzadigd vet, zout en toegevoegde suikers bevatten.

Het is belangrijk om een gevarieerd vegetarisch dieet te volgen en de voedingsmiddelen te kiezen die het beste bij je smaak en behoeften passen om ervoor te zorgen dat je alle benodigde voedingsstoffen binnenkrijgt.

Welke voedingsmiddelen kunnen klachten van opgeblazen gevoel en winderigheid veroorzaken?

Veel mensen ervaren een opgeblazen gevoel of winderigheid als hinderlijk. Dit wordt veroorzaakt door gas in de darmen. De hoeveelheid gas die ontstaat bij de spijsvertering wordt individueel bepaald door onder andere darmbacteriën. Maar gasvorming of winderigheid wordt voor het grootste gedeelte veroorzaakt door ingeslikte lucht. Vooral wanneer je tegelijkertijd praat en eet, kun je veel lucht inslikken. Maar ook kauwgom kauwen, door een rietje drinken, roken, nervositeit en een slecht passend gebit kunnen er voor zorgen dat je veel lucht inslikt.

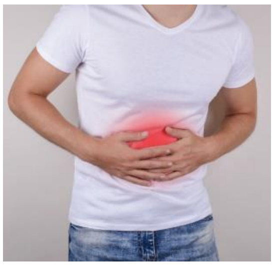

Daarnaast zijn er bepaalde voedingsmiddelen die gasvormend werken.

Voedingsmiddelen waar je extra gasvorming van kunt krijgen zijn:

1. **Koolzuurhoudende dranken:** Frisdranken, bier en bruisend water bevatten koolzuurgas, wat kan leiden tot gasophoping in de maag en darmen.
2. **Koolsoorten:** Koolsoorten zoals spruiten, broccoli, bloemkool, koolraap en boerenkool bevatten vezels en suikers die moeilijk verteerbaar zijn en gas kunnen produceren.
3. **Uien, prei en paprika:** In deze groenten zitten bepaalde suikers en vezels waar je winderig van kunt worden, vooral als ze rauw worden gegeten.
4. **Knoflook:** Knoflook bevat zwavelhoudende verbindingen die de darmbacteriën kunnen beïnvloeden en waarvan je gasvorming kunt krijgen.
5. **Peulvruchten:** Doperwten en bruine bonen zijn bekend om hun gasvormende eigenschappen vanwege de vezels en suikers die ze bevatten.

Het is belangrijk om te weten dat de gevoeligheid voor deze voedingsmiddelen verschilt van persoon tot persoon. Wat bij de ene persoon winderigheid veroorzaakt, kan bij een ander geen problemen veroorzaken. Als je merkt dat bepaalde voedingsmiddelen bij jou winderigheid veroorzaken, hoef je ze niet volledig weg te laten. Je kunt er rekening mee houden en ze bijvoorbeeld beperken tijdens speciale gelegenheden. Ook hoeft het niet zo te zijn dat je deze klachten altijd zal hebben. Probeer een voedingsmiddel over enkele weken nog eens uit.
Bovendien kan een plotselinge toename van voedingsvezels in je dieet leiden tot tijdelijke gasvorming. Voedingsvezels zijn goed voor je darmbacteriën, maar je darmen hebben misschien wat tijd nodig om zich aan te passen. Vooral in het begin kan dit tot winderigheid leiden. Als je last hebt van spijsverteringsproblemen, is het verstandig om voedingsvezels beetje bij beetje aan je dieet toe te voegen.

Tijdens een actieve opvlamming kan het verstandig zijn om **voor een korte periode** minder vezelrijke producten te gebruiken. Na de opvlamming, wanneer de ziekte weer rustig is, is het belangrijk om weer meer vezels te gebruiken om de gezondheid van je darmbacteriën te ondersteunen. (zie *Wat is het microbioom en wat doet het?)* In sommige gevallen kan het nuttig zijn om advies te vragen bij een diëtist om een uitgebalanceerd dieet samen te stellen dat geschikt is voor jouw specifieke behoeften en gevoeligheden.

Moet ik gluten uit mijn voeding weglaten?

Bij Inflammatory Bowel Disease (IBD), zoals de ziekte van Crohn en colitis ulcerosa, hebben sommige mensen last van spijsverteringsproblemen door bepaalde voedingsmiddelen, maar dit kan van persoon tot persoon verschillen. Gluten is een eiwit dat voorkomt in tarwe, gerst, rogge en sommige haverproducten. Bij mensen met coeliakie, een auto-immuunziekte , kan het gluteneiwit ernstige problemen veroorzaken. Mensen met coeliakie moeten daarom gluten weglaten uit hun voeding.

Gluten zelf veroorzaakt geen IBD, maar sommige mensen met IBD kunnen wel intolerant zijn voor gluten. Dit kan leiden tot symptomen zoals een opgeblazen gevoel, winderigheid, buikpijn, en diarree. Gluten kan bij mensen met IBD dus wel vervelende klachten geven, maar gluten beschadigt de darm niet.

Als je denkt dat gluten of bepaalde voedingsmiddelen problemen veroorzaken bij IBD, is het slim om met een diëtist of specialist in maag- en darmziekten te praten. Zij kunnen helpen om eventuele boosdoeners in je dieet op te sporen en samen met jou een voedingsplan op te stellen dat aangepast is aan jouw specifieke behoeften en gevoeligheden. Het is belangrijk om te onthouden dat voedingsproblemen, en dus ook wat wel of niet getolereerd wordt, sterk kunnen variëren van persoon tot persoon. Wat dus wat voor de ene persoon geldt, is misschien niet van toepassing op iemand anders met IBD.

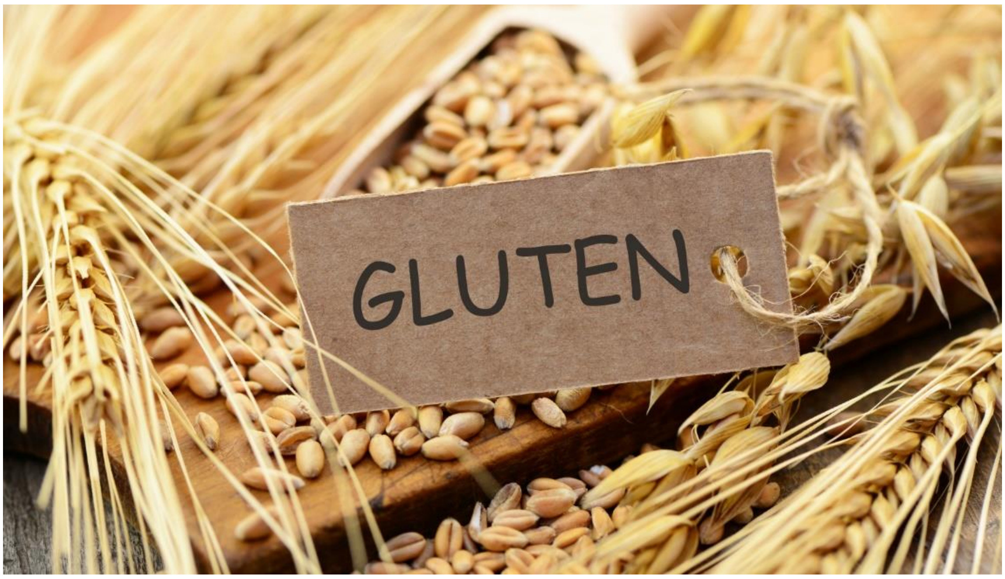

Welke voeding kan ik gebruiken om ontstekingen te voorkómen?

Het is op dit moment nog niet volledig bekend welke voeding specifiek ontstekingen in de darmen kan voorkomen. Er wordt momenteel veel onderzoek gedaan naar dit onderwerp. We verwachten de resultaten van ons lopende onderzoek in het Universitair Medisch Centrum Groningen (UMCG) eind 2025. (Campmans-Kuijpers 2023).

Wel kunnen we je vast een aantal tips geven om op te letten:

1. **Eet voldoende voedingsvezels:** Voedingsvezels zijn te vinden in groenten, fruit, volkoren graanproducten, aardappelen en noten. (zie: *Hoe kan ik voldoende voedingsvezels eten?*) Deze vezels voeden de goede bacteriën in je dikke darm en dragen bij aan een gezonder darmmicrobioom. Het eten van voedingsvezels kan helpen bij het behouden van een evenwichtige darmomgeving.
2. **Beperk rood en bewerkt vlees:** Probeer de consumptie van rood vlees en bewerkte vleeswaren zoals worstjes en vleeswaren te beperken. In plaats daarvan kun je kip, kalkoen en wild vlees eten, evenals vis, dat gunstige visvetzuren bevat. Overweeg vlees af en toe te vervangen door vegetarische alternatieven zoals peulvruchten, tempé of tofu. (Chiba 2010)(Peters, 2022). Let er dan wel steeds op dat deze producten niet erg bewerkt zijn in het bereidingsproces. Dit zie je vaak aan de hoeveelheid ingrediënten achterop de verpakking. Hoe meer ingrediënten, hoe meer bewerkt.
3. **Eet zo weinig mogelijk sterk bewerkt voedsel:** Het eten van sterk bewerkte voedingsmiddelen, zoals kant-en-klare producten met toegevoegde geur-, kleur- en smaakstoffen, kan bij sommige mensen leiden tot opvlammingen van darmziekten (Vissers 2022). Probeer het gebruik van deze kant-en-klare producten, zoals sauzen, te beperken.

Wist je dat…

… gekookte en afgekoelde zetmeelrijke producten gunstig zijn voor je darmen? Wanneer je gekookte aardappelen, rijst of pasta laat afkoelen ontstaat er resistent zetmeel. Dit type zetmeel wordt minder goed afgebroken in de darmen en dient juist als voedsel voor de gunstige bacteriën in je dikke darm. Je kunt deze koude zetmeelproducten prima in een salade verwerken of een restje aardappelen opbakken.

Referenties

Campmans-Kuijpers MJE, Dijkstra G. Food and Food Groups in Inflammatory Bowel Disease (IBD): The Design of the Groningen Anti-Inflammatory Diet (GrAID). Nutrients. 2021 Mar 25;13(4):1067. doi: 10.3390/nu13041067. PMID: 33806061; PMCID: PMC8064481

Chiba M, Abe T, Tsuda H, Sugawara T, Tsuda S, Tozawa H, Fujiwara K, Imai H. Lifestyle-related disease in Crohn's disease: relapse prevention by a semi-vegetarian diet. World J Gastroenterol. 2010 May 28;16(20):2484-95. doi: 10.3748/wjg.v16.i20.2484. PMID: 20503448; PMCID: PMC2877178.

Peters V, Bolte L, Schuttert EM, Andreu-Sánchez S, Dijkstra G, Weersma RK, Campmans-Kuijpers MJE. Western and Carnivorous Dietary Patterns are Associated with Greater Likelihood of IBD Development in a Large Prospective Population-based Cohort. J Crohns Colitis. 2022 Jul 14;16(6):931-939. doi: 10.1093/ecco-jcc/jjab219. PMID: 34864946; PMCID: PMC9282880.

Vissers E, Wellens J, Sabino J. Ultra-processed foods as a possible culprit for the rising prevalence of inflammatory bowel diseases. Front Med (Lausanne). 2022 Nov 7;9:1058373. doi: 10.3389/fmed.2022.1058373. PMID: 36419796; PMCID: PMC9676654.

Wat kan ik doen bij diarree?

Wat is diarree?

Je hebt diarree als je meerdere keren per dag (water)dunne ontlasting hebt. Diarree kan acuut zijn als deze plotseling ontstaat. Deze gaat meestal na een paar dagen vanzelf weer over. Als de diarree langer dan een paar dagen aanhoudt, raden we je aan naar de huisarts te gaan.

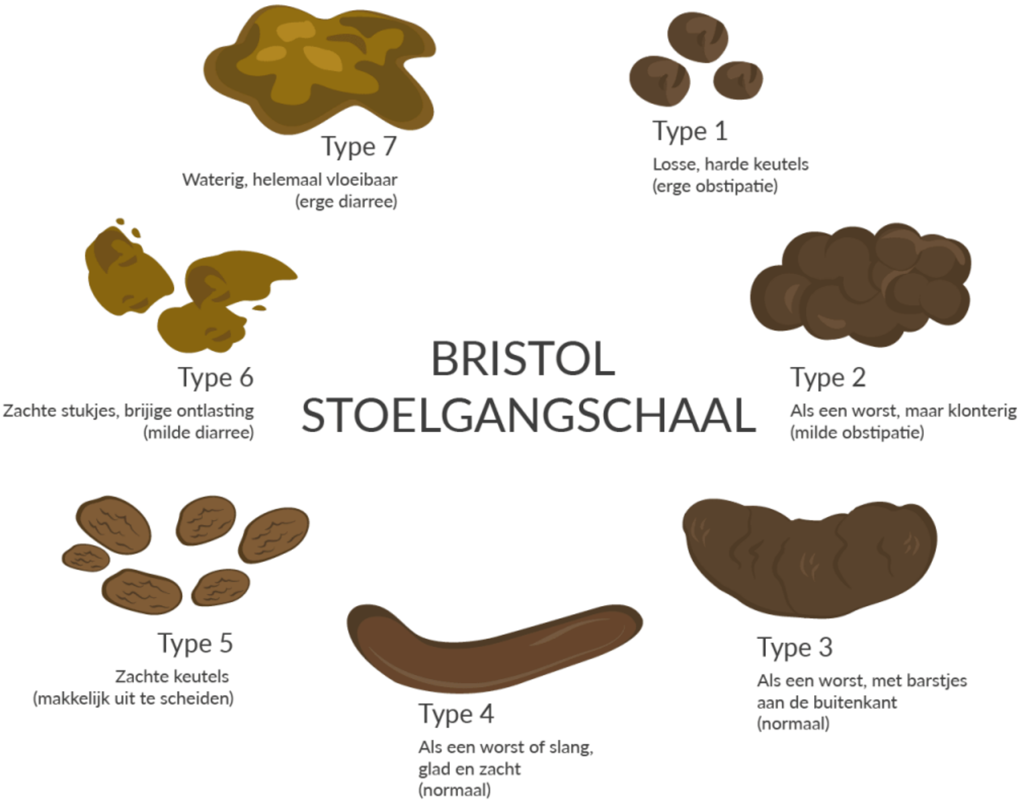

Hoe ontstaat diarree?

Onze dikke darm haalt water uit de ontlasting, waardoor de ontlasting dikker wordt. Bij diarree blijft er te veel water achter in de ontlasting. Je krijgt dunne poep. Dit kan komen doordat het lichaam extra water afscheidt naar de dikke darm, of doordat water vanuit de dikke darm minder goed wordt opgenomen.

Veel voorkomende oorzaken acute diarree:

- Een buikgriep; deze wordt meestal veroorzaakt door een virus dat tussen mensen wordt overgedragen;
- Een voedselinfectie;
- Nervositeit of psychische spanningen. Hierdoor passeert de ontlasting sneller de dikke darm, waardoor er minder tijd is om voldoende water uit de ontlasting te halen.

Andere oorzaken van diarree, vaak als diarree langer duurt:

- Aandoening van het maag-darmkanaal, zoals chronische ontstekingen aan de darm (IBD) of het prikkelbare darmsyndroom (IBS) of coeliakie;
- Een voedselallergie of -intolerantie, zoals koemelkallergie, lactose intolerantie;
- Het gebruik van bepaalde medicijnen, zoals antibiotica of laxeermiddelen;
- Soms lijkt het alsof mensen diarree hebben, terwijl ze eigenlijk last van verstopping hebben. Dunne ontlasting loopt dan langs de harde ontlasting die verstopping veroorzaakt. Dit noemen we overloopdiarree.

Dunne ontlasting door verkeerd voedingspatroon.

Wanneer de ontlasting een keer wat dunner of slapper is, hoeft dat niet meteen diarree te zijn. Dit kan vaak komen door een verkeerd voedingspatroon. Het eten van te weinig vezels komt vaak voor. Vezels worden namelijk niet verteerd en komen uiteindelijk onverteerd in de dikke darm terecht. Hier zorgen ze voor volume in de dikke darm, door water aan zicht te binden. Dit zorgt ervoor ook voor dat de ontlasting steviger wordt en dat de darmbewegingen gestimuleerd worden. Als je veel fructose (vruchtensuiker) uit vruchtensap of frisdrank gebruikt kan dit dunnere ontlasting veroorzaken. Grote hoeveelheden fructose worden namelijk niet goed opgenomen in de darm. Sommige mensen zijn hier gevoeliger voor dan andere. Ook het gebruik van bepaalde zoetstoffen die eindigen op -ol, zoals sorbitol of xylitol kan zorgen voor dunnere ontlasting.

Voedingsadvies om diarree te voorkomen

Diarree kan ook ontstaan doordat het eten of drinken besmet is met bacteriën of virussen. Het is daarom van belang om bij de bereiding van het eten goed op de hygiëne te letten:

1. Zet bederfelijke boodschappen snel in de koelkast;
2. Was je handen goed bij het bereiden van het eten en voor het eten. Let vooral op met rauw vlees en was handen na toiletbezoek;
3. Zorg dat rauw voedsel niet in aanraking komt met voedsel dat al gekookt is;
4. Zorg ervoor dat je producten voldoende verhit om bacteriën en virussen te doden. Zorg bij het opwarmen ook dat alle delen van het voedsel voldoende hoog verhit zijn (tot minstens 70°C);
5. Als je boodschappen en restjes zo snel mogelijk in de koelkast zet, bederven ze minder snel. Bacteriën kunnen zich namelijk bij een lagere temperatuur minder snel vermeerderen.

Je kunt ook diarree krijgen doordat je direct of indirect in contact komt met ontlasting van iemand die diarree heeft. Het is daarom belangrijk om altijd goed je handen te wassen na een toiletbezoek.

Voedingsadvies bij diarree

Meestal leidt het volgen van een paar voedingstips al snel tot verbetering. Hierdoor gaat de diarree vaak vanzelf weer over en hoef je niet naar de huisarts.

Hier volgen een paar algemene adviezen bij diarree:

1. **Eet waar je zin in hebt**. Blijf zo gezond mogelijk eten. Eet eventueel kleinere porties. Er zijn geen speciale voedingsmiddelen nodig. Het is een fabeltje dat witbrood of beschuit stoppend werken.
2. **Drink veel om uitdroging te voorkomen**. Omdat je met diarree veel water verliest is het belangrijk om steeds kleine hoeveelheden te drinken; in totaal uiteindelijk 2 tot 3 liter per dag om uitdroging te voorkomen. Water, thee, bouillon of verdund vruchtensap zijn geschikt. Heldere vruchtensappen zoals appelsap, druivensap of perensap zijn minder geschikt omdat hier veel fructose in zit. Dit kan bij sommige mensen juist laxerend werken. Als je overgeeft, verlies je nog eens extra water dus is voldoende drinken nog belangrijker. Ook bij warm weer, of als je zwanger of ouder dan 70 jaar bent, is het belangrijk om voldoende te drinken. Als het drinken moeilijk gaat kun je eventueel ook een ORS gebruiken. Dit is een speciale oplossing die ook de zouten weer aanvult en gemakkelijker opgenomen wordt. Indien je denkt dit nodig te hebben, kun je beter ook contact opnemen met je huisarts om je klachten te bespreken.
3. **Eet genoeg vezels**. Vezels zorgen er in de darm voor dat vocht vastgehouden wordt. Hierdoor krijgt je ontlasting een vastere structuur en meer volume. Vezels zitten alleen in plantaardige producten. Veel vezel zit in volkorenproducten zoals volkorenbrood, volkoren pasta en zilvervliesrijst, groente, fruit, noten en peulvruchten zoals bruine bonen, linzen en kikkererwten (zie: *Hoe kan ik voldoende voedingsvezels eten?*).

Medicijnen zijn bij diarree meestal niet nodig. Soms is het om praktische redenen wenselijk om diarree snel te stoppen, zoals bijvoorbeeld bij een lange reis. Overleg steeds met de huisarts of het gebruik van diarree-remmers verstandig en nodig is.

Heb je na 4 weken nog steeds last van je maag of darmen? Ga dan niet zelf experimenteren. Het zomaar weglaten van voedingsmiddelen kan leiden tot tekorten aan bepaalde voedingsstoffen in je voeding. Ga dan naar de huisarts om uit te zoeken wat er aan de hand is. Deze kan je eventueel ook doorverwijzen naar de diëtist of een Maag-, Darm- en Lever-arts**.**

Wat is galzure diarree en wat kan ik eraan doen?

Bij inflammatoire darmziekten (IBD), zoals de ziekte van Crohn en colitis ulcerosa, kunnen er problemen ontstaan met een stof genaamd "galzure zouten". Galzure zouten zijn stoffen die in de lever worden gemaakt en vervolgens in de galblaas worden opgeslagen. Uiteindelijk worden deze via de galblaas in de darmvrijgegeven om te helpen bij de vertering van vetten in onze voeding. Een groot deel van de galzure zouten wordt vervolgens weer opnieuw opgenomen in de darm om hergebruikt te kunnen worden.

Bij mensen met IBD kunnen ontstekingen in de darmen ervoor zorgen dat de heropname van galzure zouten niet goed gaat. Dit kan leiden tot de volgende problemen:

1. **Galzure diarree**: Sommige mensen met IBD kunnen een aandoening ontwikkelen genaamd "galzure diarree", waarbij er te veel galzure zouten in de dikke darm terechtkomen. Dit kan zorgen voor diarree door prikkeling van de dikke darmwand.
2. **Vette diarree**: Een verminderde opname van galzure zouten kan leiden tot problemen met de vertering van vetten. Dit kan een vettige, dunne ontlasting veroorzaken.
3. **Vitamineproblemen**: Omdat galzure zouten helpen bij het opnemen van de vetoplosbare vitamines A, D, E en K, kan een probleem met deze zouten leiden tot tekorten aan deze vitamines.

De behandeling voor deze problemen kan variëren. Zoals medicijnen om de opname van galzure zouten te verbeteren, of het aanpakken van voedingskwesties en het aanvullen van eventuele tekorten aan vitamines. Mensen met IBD die deze problemen ervaren, moeten met hun arts praten voor hulp en advies.

Wat kan ik doen bij verstopping?

Is de ontlasting hard en keutelig, de hoeveelheid gering en legen de darmen zich minder dan drie keer per week, dan spreken we van verstopping. Dit wordt ook wel obstipatie of vertraagde stoelgang genoemd. Verstopping kan worden afgewisseld met periodes met dunne ontlasting. Hierbij kunnen ook klachten als slechte eetlust, vol gevoel, winderigheid, misselijkheid, zuurbranden en hoofdpijn optreden.

Verstopping wordt vaak veroorzaakt doordat de ontlasting te lang in de darmen blijft. Dit kan zijn omdat deze te hard is doordat je te weinig drinkt. Ook kan het zijn dat je te weinig voedingsvezels gebruikt. Deze houden in de dikke darm vocht vast en zorgen zo dat de ontlasting niet te hard kan worden en sneller de darm passeert.

Maatregelen om te zorgen voor een goede darmpassage:

- Zorg dat je voeding voldoende **voedingsvezels** bevat (zie: *Hoe kan ik voldoende voedingsvezels eten?*)
- Drink voldoende **vocht**. Ten minste 2 liter per dag. Dit zijn ca 16 kopjes.
- Zorg voor een **regelmatig voedingspatroon**. Eet ten minste drie hoofdmaaltijden per dag.
- Sla het **ontbijt** niet over. Dit zorgt voor een goede peristaltische beweging in de darmen die ontlasting stimuleert.
- Zorg voor voldoende **lichaamsbeweging**. Ga bijvoorbeeld een eindje wandelen of fietsen. Dit stimuleert de peristaltiek en zorgt voor ontspanning. Stress en spanning kunnen namelijk ook een negatieve invloed op je darmen hebben.
- **Geef gehoor aan "aandrang"** om naar het toilet te gaan. De dikke darm maakt een "knijpbeweging" of peristaltische beweging als hij de voedselresten kwijt wil raken. Dit voel je als een "lichte aandrang". Als je dit voelt ga dan direct naar het toilet. Gun jezelf de tijd. Als je deze aandrang vaak negeert, kan deze ontlasting reflex op den duur verdwijnen. Zorg ook dat je toilet niet te hoog is. Bij een enigszins gehurkte houding kan de ontlasting gemakkelijker komen.

  Overleg met een arts: Als je regelmatig problemen hebt met de darmpassage, obstipatie of andere spijsverteringsproblemen, raadpleeg dan een arts voor advies en behandelingsopties.

Wat kan ik eten bij een vernauwing (stenose) van de darm?

Bij een vernauwing (stenose) in de darm is het belangrijk dat er geen grote, grove voedselresten in de ontlasting zitten zodat de ontlasting de darm kan passeren. De kans op verstopping bestaat vooral bij te snel eten, onvoldoende kauwen, niet goed schillen of niet goed koken van eten.

Als een arts jou verteld heeft dat je darm vernauwd is, dan is het belangrijk om op een aantal zaken te letten:

- Eet rustig en langzaam;
- Kauw goed (minstens 15x per hap);
- Drink voldoende (minimaal 1,5-2 liter per dag);
- Je kunt er ook voor kiezen je maaltijd goed fijn te snijden, de volledige maaltijd te pureren of fijn te malen.

Bepaalde voedingsmiddelen kunnen een afsluiting van de vernauwde darm veroorzaken. Vermijd daarom:

- Grove vezelige producten, die je met kauwen niet fijn krijgt, zoals asperges, bleekselderij, champignons en taai en draderig vlees;
- Voeding met velletjes en vezelige producten zoals citrusvruchten;
- Voeding met harde stukjes, zoals noten en pinda’s;
- Voeding met pitjes, zoals brood met zaden en pitten. Fijn volkoren is prima.

Hieronder vind je een completere lijst met voedingsmiddelen die verstopping kunnen veroorzaken wanneer er sprake is van een vernauwing in de darm:

- Asperges, artisjok;
- Bleekselderij, rabarber, aubergine, venkel;
- Champignons, bamboescheuten;
- Zuurkool, schorseneren;
- Grove rauwkost, zoals knolselderij en wortel;
- Sperziebonen en snijbonen;
- Taugé;
- Peulvruchten zoals witte bonen, bruine bonen, linzen, (split-)erwten;
- Maïs, popcorn;
- Taai en draderig vlees;
- Kokosproducten;
- Noten, zaden en pitten, pinda’s, amandelspijs;
- Citrusfruit (sap mag wel);
- Verse ananas;
- Druiven;
- Gedroogde vruchten zoals dadels, vijgen, pruimen, rozijnen, tutti frutti;
- Neem bij voorkeur geschild en ontpit fruit zonder harde, vezelige stukken en pitten.

Als je problemen hebt om op gewicht te blijven kan je arts of diëtist je (tijdelijk) drinkvoeding voorschrijven. Deze kan de darmen goed passeren en levert wat extra energie en eiwit.

Waarop moet ik bij mijn eten letten als ik een stoma heb?

Er zijn twee verschillende soorten stoma's: een stoma op de dikke darm en een stoma op de dunne darm. Met een stoma op de dikke darm (colostoma) kun je in principe alles eten en drinken, je hoeft geen speciaal dieet te volgen. Het is wel belangrijk om er op te letten dat je voldoende plast en dat de ontlasting niet te hard is. Bij een stoma op de dunne darm (ileostoma) is het belangrijk om er op te letten dat je niet te veel vocht en zout verliest[1,2] . Bij een ileostoma zijn wel wat meer voedingsadviezen. Hieronder volgen eerst algemene adviezen en vervolgens adviezen specifiek voor een colostoma en voor een ileostoma.

Algemene tips bij een stoma

1. Luister goed naar je lichaam
Het verschilt hoe je reageert op bepaalde voedingsmiddelen. Wat door de één goed wordt verdragen, kan bij een ander bijvoorbeeld verstopping veroorzaken. Bovendien kan het even duren voordat je lichaam reageert op voeding. Daarom is het belangrijk dat je alert bent op verstopping. Neem bij twijfel over bepaalde voeding contact op met je diëtist en als je problemen hebt met je stoma je stomaverpleegkundige.

2. Gasvorming
Winderigheid wordt niet alleen veroorzaakt door voedsel. Er komt ook veel lucht in het darmkanaal door: snel praten, snel eten, praten tijdens het eten, het kauwen van kauwgom, roken, drinken door een rietje, snurken met open mond, verkoudheid, zenuwachtigheid en een slecht passend gebit (Zie *Welke voedingsmiddelen kunnen klachten van opgeblazen gevoel en winderigheid veroorzaken?*). Als er te veel gas in de darmen zit, kan het zakje van de stoma bol gaan staan. Dit kan ertoe leiden dat het filter van de stoma door te veel luchtophoping sneller verstopt raakt en minder goed werkt[3].

Als je winderigheid wit voorkomen, kun je het beste de volgende producten niet of in minder mate nemen[2,4,5,6,7]:

- Bier, koolzuurhoudende dranken;
- Peulvruchten, koolsoorten, spruiten;
- Knoflook, uien, prei, paprika .

3. Sterk ruikende ontlasting
Sommige producten geven een sterke geur aan je ontlasting. Heb je liever geen sterk ruikende ontlasting, dan kun je het beste de volgende producten minder of in minder mate nemen[2,4,5,6,7]:

- Eieren
- Prei, koolsoorten, peulvruchten
- Knoflook, uien
- Vitamine B-tabletten
- Vis

Voor mensen met een colostoma of ileostoma geldt: als je het stomamateriaal op tijd vervangt en het zakje goed past, dan komen er geen geuren vrij. Het koolstoffilter in het stomazakje neemt de geuren op[2].

4. Verkleurde ontlasting
Er zijn voedingsmiddelen die een verkleuring van de ontlasting laten zien. Dit is normaal. Spinazie en ijzerpreparaten zorgen ervoor dat de ontlasting een zwarte kleur krijgt. Bieten zorgen ervoor dat de ontlasting een rode kleur krijgt[1,2,5,6,7].

Voedingstips bij een colostoma (=stoma op de dikke darm)

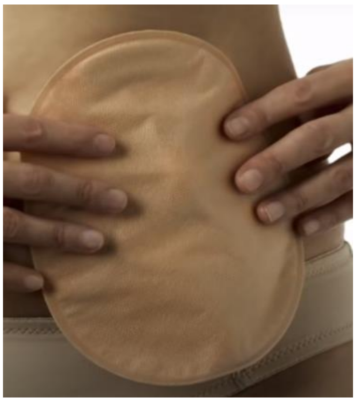

Je hoeft geen dieet te volgen, het advies is wel om de richtlijn van gezonde voeding te volgen. Het is belangrijk dat je geniet van je voeding, regelmatig eet en het eten zorgvuldig te kauwen en/of fijn te snijden. Hierbij is het belangrijk dat je voldoende vocht en vezels gebruikt[9].

1: Zorg dat je voldoende drinkt
Het is belangrijk om 1,5 tot 2 liter per dag te drinken. Dit komt overeen met 12 tot 16 kopjes per dag. Alle vloeibare producten tellen mee, ook soep, yoghurt en vla[2,5,7].

2: Eet voldoende vezels
Vezels zorgen ervoor dat de ontlasting een goede vastigheid krijgt en om het dun en soepel te houden. Vezels komen voor in plantaardige producten zoals volkorenbrood, roggebrood, fruit, groente, rauwkost, aardappelen, peulvruchten en zilvervliesrijst. Mensen met een colostoma kunnen een verstopping krijgen. Als de ontlasting regelmatig hard is en moeizaam in het zakje gaat, is het verstandig om meer vezels aan je voeding toe te voegen en meer te drinken[2]. Het advies is om 30-40 gram vezels per dag te eten[5,7,8] **(**voor uitgebreider advies over vezels zie: *Hoe kan ik voldoende voedingsvezels eten?*)

Voedingstips bij een ileostoma (=stoma op de dunne darm)

Je hoeft geen dieet te volgen. Het is belangrijk dat je geniet van je voeding, regelmatig eet en het eten zorgvuldig kauwt en/of fijn snijdt. Hierbij is het belangrijk dat je voldoende vocht, zout en vezels eet[9].

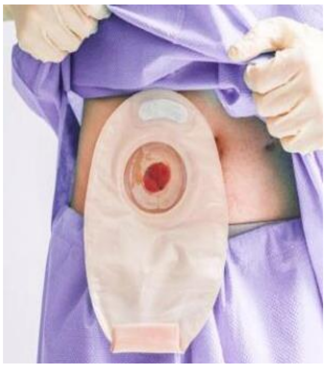

Tip 1: Zorg dat je voldoende drinkt
Het is belangrijk om 2 tot 2,5 liter per dag te drinken. Dit komt overeen met 16 tot 20 kopjes per dag. Dit is iets meer dan de normale aanbevolen hoeveelheid. Dat komt omdat je bij een stoma op de dunne darm automatisch wat meer vocht verliest doordat er geen dikke darm meer is om water terug op te nemen. Alle vloeibare producten tellen mee, ook soep, yoghurt en vla[2,8].

Tip 2: Let op vocht- en zoutverlies
Doordat de dikke darm geen water en zouten terug opneemt, zul je extra veel vocht en zout verliezen. Dit gebeurt onder andere bij diarree, braken, hevige transpiratie, koorts of warm weer. Maar ook bij het sporten verlies je meer vocht en zout dan normaal. Het aanvullen van vocht en zout kan dan nodig zijn. Overleg hierover steeds met de diëtist of stomaverpleegkundige.

Tip 3: Zorg voor extra zoutinname
Mensen met een ileostoma krijgen het advies extra zout te gebruiken. Doordat de dikke darm ontbreekt, verliest je veel zout en vocht met de ontlasting. Een tekort aan zout kan leiden tot verschillende klachten[2,8]. Producten met zout zijn bijvoorbeeld soep, bouillonblokje, soep, sojasaus en chips. Maar ook in brood, pizza's en kant-en-klaar maaltijden zit veel zout. (zie ook: Tabel. Producten die veel zout leveren.

Tip 4: Kauw je eten goed
Eet rustig, kauw het eten goed en geniet van je eten. Sommige voedingsmiddelen zijn draderig of verteren niet zo gemakkelijk. Zij kunnen tot verstopping van de stoma leiden als ze niet goed fijngesneden en goed gekauwd zijn. Dit zijn bijvoorbeeld[2,5,7,8]:

- Grove tarwezemelen, popcorn, zaden, noten en pitten.
- Taai en draderig vlees, worstvellen of vis met graten.
- Groente met harde nerven, rauwkost, vezelige draderige groenten zoals: asperges, bleekselderij, schorseneren, zuurkool, taugé, draderige sperziebonen en snijbonen, peulvruchten, doperwten, maïs, rabarber, paddenstoelen en champignons.
- Fruit met pit en schil, partjes citrusfruit, ananas, bessen, druiven, kiwi, kokosproducten, gedroogde vijgen, rozijnen, dadels en pruimen.

Tip 5: Eet regelmatig kleine maaltijden
Als je het eten en drinken goed verspreidt over de dag, zal de ontlasting beter indikken. Het eten van 4 of 5 kleine maaltijden (met drinken) is beter dan 1 grote en een paar kleine maaltijden. Eet niet te veel voordat je gaat slapen. Dan kun je 's nachts last krijgen van de producerende stoma[2,5].

Tip 6: Let op te dunne ontlasting
De ontlasting is meestal brijig tot dun als je een ileostoma hebt. Om de ontlasting niet te dun te laten worden, kan het helpen om bij het drinken steeds extra zetmeelrijke producten te eten, zoals beschuit, biscuit, cracker, aardappels, pasta of rijst[2]. De ontlasting bij een ileostoma zal echter altijd wat dunner blijven dan gebruikelijk.

Tip 7: Hygiëne op vakantie
Wanneer je in (sub)tropische landen verblijft, heb je te maken met hogere temperaturen en andere hygiënische omstandigheden. Hierdoor loop je meer risico op het krijgen van waterdunne ontlasting en een vocht- en/of zouttekort. Neem geen onnodige risico's. Vermijd het gebruik van ongekookt leidingwater, ijs en ijsblokjes, ongeschild fruit, rauwe groenten, rauw vlees en rauwe vis. Zorg er ook voor dat je altijd O.R.S., bouillontabletten en/of zouttabletten bij je hebt.

Referentielijst

1. Voeding - Leven met een stoma - Stomavereniging. (2021, August 11). Stomavereniging. https://www.stomavereniging.nl/leven-met-een-stoma/voeding/
2. Van Batenburg, C., & Davidson, B. (2019, December). Voedingstips bij stoma of pouch. Stoma Vereniging. https://www.stomavereniging.nl/wp-content/uploads/2021/03/Brochure-Voeding-2020_LR8.pdf
3. Stomaproblemen: behandelingen en adviezen | St. Antonius Ziekenhuis. (n.d.). https://www.antoniusziekenhuis.nl/stomaproblemen-behandelingen-en-adviezen
4. Voedingsadvies bij een colostoma. (2023). Bernhoven. https://www.bernhoven.nl/folders/voedingsadvies-bij-een-colostoma/
5. Voedingsadvies bij een stoma Diëtetiek. (n.d.). Ikazia Ziekenhuis. https://www.ikazia.nl/assets/folders/800536-NL.pdf
6. Voedingsadviezen voor mensen met een ileostoma | St. Antonius Ziekenhuis. (n.d.). https://www.antoniusziekenhuis.nl/voedingsadviezen-voor-mensen-met-een-ileostoma
7. TeamVKInfo_Merel. (2022). Wat kan ik eten als ik een darmstoma heb? Voeding En Kanker Info. https://www.voedingenkankerinfo.nl/wat-kan-ik-eten-met-een-darmstoma/
8. Maag Lever Darm Stichting. (2022, August 3). Stoma - Maag Lever Darm Stichting. https://www.mlds.nl/ziekten/operaties/stoma/
9. Richtlijn Stomazorg Nederland. (2016, November 6). V&VN Stomaverpleegkundigen. https://www.venvn.nl/media/rekjq2s4/richtlijn-stomazorg-nederland.pdf?fileticket=r_6xShh5UsU%3d&portalid=1

Zijn er speciale dingen waar ik bij mijn eten op moet letten als ik een pouch heb?

Bij een pouch is de dikke darm verwijderd en is er een verbinding gemaakt tussen de dunne darm en de anus. Van het laatste deel van de dunne darm is een reservoir gemaakt, de pouch.

Normaal gesproken wordt in de dikke darm ontlasting ingedikt door water en zouten opnieuw in het lichaam op te nemen. Ook wordt in de dikke darm de ontlasting verzameld. Deze reservoirfunctie wordt na verloop van tijd overgenomen door het nieuwe reservoir, de pouch. Mensen met een goed werkende pouch hebben gemiddeld 6 tot 8 keer ontlasting per dag.

Na het creëren van een pouch hoef je geen speciaal dieet te volgen. Je mag alles eten. Het is echter handig om op een aantal zaken te letten bij je voeding:

Behoefte aan vocht

Als je de pouch hebt, kun je vooral in het begin veel vocht verliezen met de ontlasting. Daarom is het belangrijk om verdeeld over de dag 2 tot 2,5 liter te drinken. Dit zijn ongeveer 16 tot 20 kopjes. Je kunt alle vloeibare producten meetellen zoals water, koffie, thee, soep, vla en yoghurt.

Je drinkt voldoende als je urine licht van kleur is. Als je weinig plast of je urine heeft een donkere kleur, kan dit een teken zijn dat je te weinig vocht hebt gedronken.

Bij een vochttekort kun je de volgende klachten ervaren:

- Dorst
- Droge mond/ slijmvliezen
- Droge huid
- Lusteloosheid/ sufheid
- Verminderde eetlust
- Rusteloosheid
- Verwardheid
- Snel gewichtsverlies

Behoefte aan zout

Bij waterdunne ontlasting verlies je niet alleen water maar ook zout met de ontlasting. Het is daarom belangrijk om dit verlies aan zout aan te vullen door extra zout te gebruiken. Je kunt extra zout over het eten strooien, of voedingsmiddelen eten die veel zout bevatten zoals chips en soep.

Een aantal producten met zout bevat echter ook veel calorieën. Als je te zwaar dreigt te worden, kun je beter producten gebruiken die wel zout bevatten en weinig calorieën leveren.

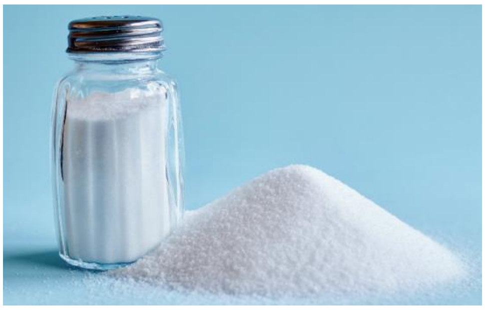

Tabel. Producten die veel zout leveren.

Producten die veel zout leveren	
Met weinig calorieën	Met veel calorieën
Bouillon<br>Curry<br>Groentesoep/tomatensoep<br>Heldere soep<br>20+ en 30+ (smeer)kaas<br>Ketjap, zoet en zuur<br>Rauwe ham<br>Rookvlees<br>Smaakversterker<br>Soepversterker<br>Tomatenketchup	Bacon<br>Cervelaatworst<br>Chips en zoutjes<br>Haring<br>48+ (smeer)kaas<br>Ontbijtspek<br>Rookworst<br>Salami

Drop bevat geen keukenzout, maar salmiakzout,. Dit vult niet het zouttekort aan.

Wanneer je te weinig zout binnenkrijgt, kan dit tot problemen leiden. Ga met een pouch nooit over op een zout (natrium) beperkt dieet zonder overleg met je arts of diëtist.

Bij een zouttekort kun je de volgende klachten ervaren:

- Braken
- Ernstige hoofdpijn
- Misselijkheid
- Opgeblazen gevoel
- Verwardheid

Voorzichtig zijn bij extra zout- en vochtverlies: Bij waterdunne ontlasting, braken, overmatig zweten, koorts of warm weer verlies je extra zout en vocht. Het kan ook leiden tot verminderde eetlust, waardoor je minder zout en vocht binnenkrijgt. Het is dan noodzakelijk om extra zout en vocht in te nemen om tekorten te voorkomen. O.R.S. (Oral Rehydration Salts) opgelost in water kan nuttig zijn. Deze oplossing bevat water, suiker en zout, en is verkrijgbaar bij de apotheek en drogist. Voor vragen over het gebruik van O.R.S. kun je het beste contact opnemen met je arts, apotheker of diëtist.

Wanneer je minder vaak ontlasting hebt en de ontlasting steviger wordt, neemt ook het verlies van zout en vocht af. Dan hoef je minder aandacht te besteden aan het bovenstaande advies over zout en vocht.

Stevigheid van de ontlasting. Om de ontlasting dikker te maken, is het het beste om je voedsel en drinkvocht goed te verspreiden over de dag. Het wordt ook aanbevolen om een gezonde voeding met voldoende voedingsvezels te gebruiken. Voedingsvezels zitten in plantaardige producten zoals volkorenbrood, roggebrood, fruit, groente, rauwkost, aardappelen, zilvervliesrijst volkorenpasta, en peulvruchten. Voedingsvezels nemen water op en houden het vast. Hierdoor kan de ontlasting dikker worden. (zie: *Hoe kan ik voldoende voedingsvezels eten?*) Als de ontlasting te dun is, kun je jouw drinken combineren met een zetmeel- en vezelrijk product, zoals een volkoren boterham of volkoren cracker.

Gasvorming kan ervoor zorgen dat ontlasting ongewenst wordt verloren. De hoeveelheid gas die tijdens de spijsvertering ontstaat, is grotendeels afhankelijk van factoren zoals darmbacteriën en varieert van persoon tot persoon. Winderigheid wordt voornamelijk veroorzaakt door lucht inslikken, vooral wanneer je praat en eet tegelijk. Maar ook kauwgom kauwen, drinken met een rietje, roken, nervositeit en een slecht passend gebit kunnen leiden tot het inslikken van veel lucht.

Bovendien zijn er bepaalde voedingsmiddelen die gasvorming kunnen veroorzaken. Het is meestal niet nodig om deze voedingsmiddelen volledig te vermijden. Je kunt er rekening mee houden, vooral bij speciale gelegenheden zoals feestjes of een avondje uit. Als je last krijgt van bepaalde voedingsmiddelen, betekent dit niet noodzakelijk dat dit altijd het geval zal zijn. Het kan nuttig zijn om het product na enkele weken opnieuw te proberen.

Producten die extra gasvorming kunnen veroorzaken zijn koolzuurhoudende dranken, bier, koolsoorten, spruiten, uien, prei, paprika, knoflook en peulvruchten (zoals doperwten en bruine bonen) (zie: *Welke voedingsmiddelen kunnen klachten van opgeblazen gevoel en winderigheid veroorzaken?*).

Anale irritatie Als de ontlasting dun is en meerdere keren per dag komt, kan de huid rond de anus geïrriteerd raken en dus pijnlijk worden. Dit kan vooral kort na de operatie gebeuren. Het is daarom belangrijk om de huid rond de anus goed te verzorgen om irritatie te voorkomen. Je kunt hierover informatie krijgen van je specialist en/of stomaverpleegkundige. Voeding veroorzaakt geen anale irritatie. Als de huid beschadigd is, kunnen sterk gekruide gerechten mogelijk meer irritatieklachten veroorzaken na het eten.

Pouchitis en probiotica Pouchitis is een ontsteking van de pouch. De oorzaak van pouchitis is nog niet bekend, maar wel dat er darmbacteriën bij betrokken zijn. Momenteel wordt er veel onderzoek gedaan naar de rol van probiotica bij het voorkomen en behandelen van pouchitis. De resultaten van sommige onderzoeken naar bepaalde probiotica zijn veelbelovend. Probiotica zijn speciaal geselecteerde bacterieculturen die, wanneer ze worden ingenomen, de groei van schadelijke bacteriën en virussen in de darmen kunnen remmen. Niet alle probiotica hebben hetzelfde effect, dus gebruik niet zomaar willekeurige probiotica. Tot nu toe is er alleen voldoende bewijs voor het gebruik van een specifieke bacterie cultuur genaamd 'VSL#3' bij mensen met een pouch. Onderzoek laat zien dat deze bacteriecultuur effectief ontsteking van de pouch kan voorkomen. Een nadeel van probiotica is dat ze vaak dagelijks en langdurig moeten worden ingenomen om pouchitis te voorkomen, en ze kunnen kostbaar zijn. Bij een ernstige pouchontsteking blijven antibiotica echter nog steeds de eerste keuze voor behandeling[1]. Overleg hierover altijd eerst met je arts en/of diëtist.

Met andere woorden: er is alleen bewijs dat probiotica (VSL#3) effectief kunnen zijn bij mensen met een pouch. Voor alle andere situaties is er geen bewijs dat probiotica effectief zijn bij ontsteking.

Vakantie Wanneer je in (sub)tropische landen verblijft, heb je te maken met hogere temperaturen en andere hygiënische omstandigheden. Hierdoor loop je meer risico op het krijgen van waterdunne ontlasting en een vocht- en/of zouttekort. Neem geen onnodige risico's. Vermijd het gebruik van ongekookt leidingwater, ijs en ijsblokjes, ongeschild fruit, rauwe groenten, rauw vlees en rauwe vis. Zorg er ook voor dat je altijd O.R.S., bouillontabletten en/of zouttabletten bij je hebt. (zie ook: *Waar moet ik bij mijn voeding op letten als ik op ga?*)

Referenties

1. Gionchetti P, Calafiore A, Riso D, Liguori G, Calabrese C, Vitali G, Laureti S, Poggioli G, Campieri M, Rizzello F. The role of antibiotics and probiotics in pouchitis. Ann Gastroenterol. 2012;25(2):100-105. PMID: 24714229; PMCID: PMC3959382

Moet ik extra vitamine preparaten nemen?

Een gezonde en gevarieerde voeding is volwaardig en bevat alle vitaminen en mineralen die je nodig hebt. Het innemen van supplementen is alleen nodig als er bij jou tekorten worden gevonden. Hierop zijn 4 uitzonderingen[1]:

- **Calcium in combinatie met vitamine D:** Dit kan nodig zijn bij het gebruik van corticosteroïden, omdat deze medicijnen de opname van calcium kunnen verminderen. Vitamine D helpt het lichaam calcium beter op te nemen.
- **Foliumzuur (vitamine B11):** Dit kan nodig zijn bij het gebruik van Methotrexaat, aangezien foliumzuur de bijwerkingen van dit geneesmiddel kan verminderen. Hiervoor moet u foliumzuur 1 keer in de week nemen, minstens 24 uur nadat u methotrexaat heeft gebruikt.
- **Vitamine B12:** Dit kan nodig zijn als meer dan 20 cm van het laatste deel van de dunne darm is verwijderd, omdat de opname van vitamine B12 in dat deel van het darmstelsel plaatsvindt.
- **IJzer:** IJzersupplementen kunnen nodig zijn bij bloedarmoede of als er bloedverlies is bij de ontlasting, omdat ijzer helpt bij het aanvullen van rode bloedcellen en het verhogen van het ijzergehalte in het bloed.

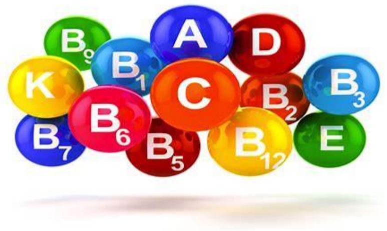

Vitamine D

Vitamine D is een belangrijke voedingsstof die invloed heeft op het immuunsysteem in de darmen[2]. Bij mensen met een te lage vitamine D-waarde in het bloed zien we vaker meer ziekteactiviteit, ontsteking van de darmslijmvliezen, meer opvlammingen van de ziekte en een slechtere kwaliteit van leven[3]. Daarnaast is vitamine D ook erg belangrijk voor het onderhoud van onze botten. Daarom is het goed voor mensen met IBD om voldoende vitamine D binnen te krijgen. Belangrijke bronnen van vitamine D zijn vooral vette vis (zoals zalm, forel en makreel), en in mindere mate vlees en eieren. Vitamine D wordt toegevoegd aan halvarine, margarine en bak- en braadproducten. Ook wordt vitamine D in ons lichaam aangemaakt onder invloed van de zon. Vitamine D kan ook in de vorm van supplementen worden ingenomen. De aanbevolen dagelijkse hoeveelheid vitamine D voor iedereen is 10 microgram.

Bij mensen met IBD die actieve ziekte hebben en bij mensen die met steroïden worden behandeld, moet het calcium- en vitamine D-gehalte worden gecontroleerd en indien nodig als supplement worden gegeven om botontkalking voorkomen[1]. Als je hier vragen over hebt, is het het beste om dit te bespreken met je maag-, darm-, leverarts of huisarts.

Vitamine B

Een tekort aan foliumzuur (vitamine B11) en vitamine B12 komt regelmatig voor bij mensen met IBD, vooral bij mensen met de ziekte van Crohn. Zo'n tekort kan leiden tot bloedarmoede en moet regelmatig gecontroleerd worden, vooral bij mensen met de ziekte van Crohn bij wie meer dan 20 cm van het laatste deel van de dunne darm is verwijderd. Dit komt doordat vitamine B12 normaal gesproken in dit deel van de darm wordt opgenomen[4]. Als er meer dan 20 cm van het laatste deel van de dunne darm is verwijderd en er een tekort is aan vitamine B12, zal de arts vitamine B12-injecties voorschrijven na overleg.

Patiënten die methotrexaat of sulfasalazine gebruiken lopen een groter risico op een tekort aan foliumzuur door de werking van de medicijnen. Daarom krijgen zij extra foliumzuur voorgeschreven[4].

Vitamine C

Vitamine C is een antioxidant. Antioxidanten vangen schadelijke stoffen in het lichaam op. Ook helpt vitamine C bij de opname van ijzer. Mensen met IBD die weinig groente en fruit eten, kunnen sneller een tekort aan vitamine C hebben. Als je voldoende groente, fruit en/of aardappelen eet, hoef je geen extra vitamine C in te nemen.

Referenties:

1. Bischoff SC, Bager P, Escher J, Forbes A, Hébuterne X, Hvas CL, Joly F, Klek S, Krznaric Z, Ockenga J, Schneider S, Shamir R, Stardelova K, Bender DV, Wierdsma N, Weimann A. ESPEN guideline on Clinical Nutrition in inflammatory bowel disease. Clin Nutr. 2023 Mar;42(3):352-379. Doi: 10.1016/j.clnu.2022.12.004. Epub 2023 Jan 13. PMID: 36739756

2. Bakke D, Sun J. Ancient nuclear receptor vdr with new functions: Microbiome and inflammation. *Inflamm Bowel Dis* (2018) 24(6):1149–54. Doi: 10.1093/ibd/izy092).

3. Gubatan J, Chou ND, Nielsen OH, Moss AC. Systematic review with meta-analysis: Association of vitamin d status with clinical outcomes in adult patients with inflammatory bowel disease. *Aliment Pharmacol Ther* (2019) 50(11-12):1146–58. Doi: 10.1111/apt.15506

4. Bischoff SC, Escher J, Hébuterne X, Kłęk S, Krznaric Z, Schneider S, et al. Espen practical guideline: Clinical nutrition in inflammatory bowel disease. *Clin Nutr* (2020) 39(3):632–53. Doi: 10.1016/j.clnu.2019.11.002

Heeft het voordelen om probiotica te gebruiken bij de behandeling van IBD?

Het gebruik van probiotica bij de behandeling van IBD (Inflammatory Bowel Disease) is een onderwerp van discussie en onderzoek.

Probiotica worden vaak gebruikt bij de behandeling van IBD, maar er is momenteel beperkt wetenschappelijk bewijs dat aantoont dat ze effectief zijn. Probiotica zijn levende micro-organismen die, wanneer in voldoende hoeveelheden geconsumeerd, mogelijk gunstige effecten kunnen hebben op de darmgezondheid.

Op basis van het beschikbare bewijs is er nog geen aanbeveling om probiotica te gebruiken voor mensen met de ziekte van Crohn. Er is echter wel voldoende bewijs voor het gebruik van een specifieke bacteriecultuur genaamd 'VSL#3' bij mensen met een pouch. Onderzoek heeft aangetoond dat deze bacteriecultuur effectief kan zijn bij het voorkomen van ontsteking van de pouch[1]. Bij een ernstige ontsteking van de pouch blijven antibiotica echter de eerste behandeloptie.

Er zijn enkele onderzoeken gedaan bij mensen met colitis ulcerosa, die aantoonden dat bepaalde bacterieculturen (*Escherichia coli Nissle* 1917) de samenstelling van het darmmicrobioom kunnen veranderen[2]. In de toekomst zou probiotica mogelijk kunnen worden gebruikt bij mensen met colitis ulcerosa om de ziekte tot rust te brengen[3]. Het huidige bewijs is nog onvoldoende. Probiotica worden niet aanbevolen voor de behandeling van IBD bij mensen. De uitzondering hierop is het gebruik van VSL#3 bij pouchitis. (zie: *Zijn er speciale dingen waar ik bij mijn eten op moet letten als ik een pouch heb?)*

Het advies is om terughoudend te zijn met het gebruik van dure probiotica en in plaats daarvan te streven naar een volwaardige, gezonde voeding met voldoende voedingsvezels. De keuze om probiotica te gebruiken moet altijd in overleg met een arts of diëtist worden genomen.

Referenties:

1. Gionchetti P, Calafiore A, Riso D, Liguori G, Calabrese C, Vitali G, Laureti S, Poggioli G, Campieri M, Rizzello F. The role of antibiotics and probiotics in pouchitis. Ann Gastroenterol. 2012;25(2):100-105. PMID: 24714229; PMCID: PMC3959382

2. Zhao Z, Xu S, Zhang W, Wu D, Yang G. Probiotic Escherichia coli NISSLE 1917 for inflammatory bowel disease applications. Food Funct. 2022 Jun 6;13(11):5914-5924. doi: 10.1039/d2fo00226d. PMID: 35583304.

3. Lorentz A, Müller L. Probiotics in the Treatment of Inflammatory Bowel Disease in Adulthood: A Systematic Review. J Gastrointestin Liver Dis. 2022 Mar 19;31(1):74-84. doi: 10.15403/jgld-3936. Erratum in: J Gastrointestin Liver Dis. 2022 Sep 16;31(3):360. PMID: 35306546

Heeft het voordelen om omega 3 supplementen of visolie te gebruiken?

Omega 3-vetzuren zijn speciale soorten vetzuren, waaronder eicosapentaeenzuur (EPA) en docosahexaeenzuur (DHA), die vaak "visvetzuren" worden genoemd. Een plantaardige omega 3-vetzuur, alfalinoleenzuur (ALA), vind je in lijnzaadolie, raapzaadolie, sojaolie, walnoten en sommige margarines. Men denkt dat deze vetzuren ontstekingsremmende eigenschappen hebben[1].

Als je minstens één keer per week vis eet, krijg je meestal genoeg van deze visvetzuren binnen. Het is het beste om vette vis, zoals haring of zalm, te kiezen[1]. (zie ook: *Welke soorten vet kan ik het beste eten als ik IBD heb?)*

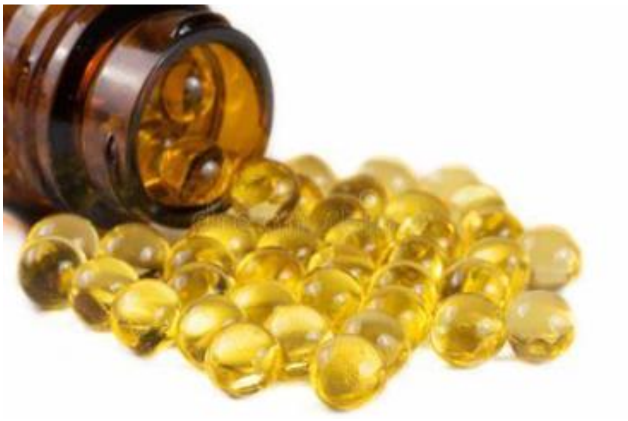

Omega 3-supplementen bij IBD

Onderzoeken bij verschillende aandoeningen tonen mogelijk enkele ontstekingsremmende effecten van omega-3 vetzuren. Echter, uit onderzoeken uitgevoerd bij patiënten met zowel de ziekte van Crohn als colitis ulcerosa komt er onvoldoende bewijs dat aantoont dat het gebruik omega-3 supplementen effect heeft op het voorkomen of behandelen van deze aandoeningen[2,3,4] . Om deze reden heeft suppletie van deze vetzuren vooralsnog geen plaats in de behandeling van IBD. Probeer wel zoveel mogelijk omega-3 vetzuren rechtstreeks uit vette vis te halen of ALA uit de genoemde plantaardige bronnen. Het is altijd verstandig om met een arts of diëtist te overleggen voordat je besluit om toch supplementen te gebruiken.

Referenties

1. Omega 3 | Voedingscentrum
2. Richman, E. and Rhodes, J.M. (2013), Review article: evidence-based dietary advice for patients with inflammatory bowel disease. Aliment Pharmacol Ther, 38: 1156-1171. *https://doi.org/10.1111/apt.12500*
3. Ajabnoor, S.M., Thorpe, G., Abdelhamid, A. *et al.* Long-term effects of increasing omega-3, omega-6 and total polyunsaturated fats on inflammatory bowel disease and markers of inflammation: a systematic review and meta-analysis of randomized controlled trials. *Eur J Nutr* **60**, 2293–2316 (2021). https://doi.org/10.1007/s00394-020-02413-y
4. Bischoff SC, Bager P, Escher J, Forbes A, Hébuterne X, Hvas CL, Joly F, Klek S, Krznaric Z, Ockenga J, Schneider S, Shamir R, Stardelova K, Bender DV, Wierdsma N, Weimann A. ESPEN guideline on Clinical Nutrition in inflammatory bowel disease. Clin Nutr. 2023 Mar;42(3):352-379. doi: 10.1016/j.clnu.2022.12.004. Epub 2023 Jan 13. PMID: 36739756.

Is het verstandig om kurkuma te gebruiken?

Op dit moment is er onvoldoende bewijs om het gebruik van kurkuma aan te raden aan patiënten met IBD.

Er is één studie bij 89 patiënten met colitis ulcerosa die na 6 maanden gebruik enige verbetering opmerkt in ontstekingswaarden. Echter na 12 maanden gebruik was er geen voordeel meer te zien. Er zijn momenteel geen onderzoeken bij patiënten met de ziekte van Crohn bekend [1, 2].

Bovendien kan het gebruik van kurkuma in grote hoeveelheden leiden tot milde bijwerkingen, zoals verstoring van de maag en darmen, een opgeblazen gevoel, hoofdpijn, misselijkheid, zuurbranden en huidallergie[3,4,5,6].

Daarom is er momenteel onvoldoende bewijs om het gebruik van kurkuma aan te bevelen voor het bereiken of behouden van remissie bij IBD.

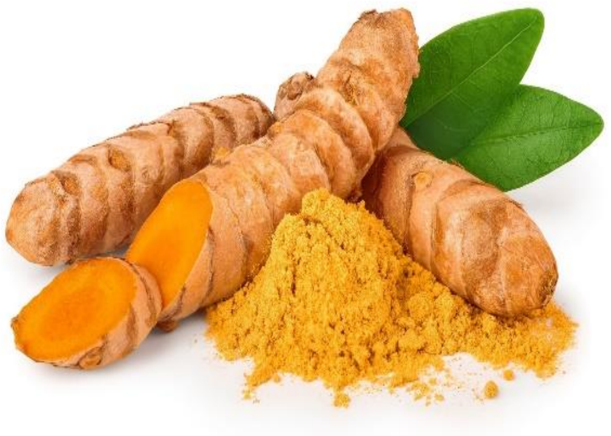

Literatuur

1. Coelho, M.R., Romi, M.D., Masterson Tavares Pereira Ferreira, D., Zaltman, C., Soares-Mota, M. (2020) The use of curcumin as a complementary therapy in ulcerative colitis: a systematic review of randomized controlled clinical trials. Nutrients. Doi10.3390/nu12082296
2. Hanai, H., Kanauchi, O., Mitsuyama, K., Andoh, A., Takeuchi, K., Takayuki, I., . . . Bamba, T. (2004). Germinated barley foodstuff prolongs remission in patients with ulcerative colitis. Int J Mol Med, 13(5), 643-647.
3. Goulart de Alvares, R. , Barbalho, S., Rubira, C.J., Araújo, A.C., Lima, V.M., Leoni, B.R., Guiguer E.L. (2020) Curcumin therapy for ulcerative colitis remission:systematic review and meta-analysis. Expert review of gastroenterology&hepatitis.
4. Lang, A., Salomon, N., Wu, J. C., Kopylov, U., Lahat, A., Har-Noy, O., . . . Ben-Horin, S. (2015). Curcumin in Combination With Mesalamine Induces Remission in Patients With Mild-to-Moderate Ulcerative Colitis in a Randomized Controlled Trial. Clin Gastroenterol Hepatol, 13(8), 1444-1449 e1441. doi:10.1016/j.cgh.2015.02.019
5. Kedia, S., Bhatia, V., Thareja, S., Garg, S., Mouli, V. P., Bopanna, S., . . . Ahuja, V. (2017). Low dose oral curcumin is not effective in induction of remission in mild to moderate ulcerative colitis: Results from a randomized double blind placebo controlled trial. World J Gastrointest Pharmacol Ther, 8(2), 147-154. doi:10.4292/wjgpt.v8.i2.147
6. Sadeghi, N., Mansoori, A., Shayesteh, A. (2019) The effect of curcumin supplementation on clinical outcomes and inflammatory markers in patients with ulcerative colitis. Phytotherapy research, 34, 1123-1133

Wat is het FODMAP dieet en is het verstandig om dit te gebruiken?

Wat is het FODMAP dieet?

Het FODMAP-dieet is bedoeld voor mensen met gevoelige darmen, omdat het zich richt op suikers in voedsel die darmklachten kunnen veroorzaken. Dit dieet wordt vaak aanbevolen voor mensen met het prikkelbare darmsyndroom (PDS). PDS-patiënten hebben last van buikklachten, maar er zijn geen ontstekingen in hun darmen vastgesteld. FODMAP staat voor **F**ermenteerbare **O**ligosachariden, **D**isachariden, **M**onosachariden **A**nd **P**olyolen. Deze voedingsmiddelen worden moeilijk opgenomen in de dunne darm en kunnen extra gas in de dikke darm veroorzaken. Ze zijn te vinden in bepaalde groenten, fruitsoorten, tarwe en dierlijke zuivelproducten. Bij het FODMAP-beperkt dieet leert een diëtist je welke voedingsmiddelen klachten veroorzaken. Het dieet begint met een testfase van 6 weken waarin je bepaalde voedingsmiddelen vermijdt om te zien of je klachten verminderen. Het is echter belangrijk om na deze fase de weggelaten producten weer één voor één te introduceren, onder begeleiding, om te voorkomen dat je een onevenwichtige voeding krijgt.

Kunnen mensen met IBD het FODMAP dieet volgen?

De behandeling bij mensen met IBD richt zich vooral op het verminderen en voorkomen van ontstekingen in de darm. Veel mensen met IBD ervaren echter ook symptomen van diarree, buikpijn, vermoeidheid, een opgeblazen gevoel en winderigheid ook als de ontsteking rustig is (zie: *Welke voedingsmiddelen kunnen klachten van opgeblazen gevoel en winderigheid veroorzaken?*). Ze kunnen naast IBD ook last hebben van het prikkelbare darmsyndroom (PDS), wat voorkomt bij 30-45% van de mensen met IBD[1]. Onderzoek heeft aangetoond dat het FODMAP-beperkte dieet ook bij mensen met IBD in de rustige fase deze symptomen kan verminderen. Het is echter van groot belangrijk om dit onder begeleiding van een diëtist te doen, omdat in het begin veel voedingsmiddelen worden weggelaten en later weer worden geïntroduceerd om een evenwichtige voeding te waarborgen. Als dit niet goed wordt uitgevoerd, kunnen er tekorten in de voeding ontstaan. Hier zijn mensen met IBD vaak ook extra gevoelig voor.

Een recent onderzoek heeft aangetoond dat het FODMAP-beperkte dieet kan helpen bij het verminderen van symptomen zoals een opgeblazen gevoel, winderigheid en pijn. Maar het geeft geen verbetering van de samenstelling van de ontlasting of ontsteking van de darmwand[1]. Belangrijk is dus om te onthouden dat het FODMAP dieet mogelijk kan bijdragen aan verminderen van klachten, maar dus niet de ziekte zelf zal oplossen.

Referenties

1. Peng Z, Yi J, Liu X. A Low-FODMAP Diet Provides Benefits for Functional Gastrointestinal Symptoms but Not for Improving Stool Consistency and Mucosal Inflammation in IBD: A Systematic Review and Meta-Analysis. Nutrients. 2022 May 15;14(10):2072. doi: 10.3390/nu14102072. PMID: 35631213; PMCID: PMC9146862

2. Kan het FODMAP-dieet u helpen? (martiniziekenhuis.nl)

Wat is het CDED dieet en is het geschikt voor mij?

De laatste jaren is er steeds meer aandacht voor het Crohn's dieet exclusie dieet (CDED). Het CDED lijkt mogelijk te helpen bij het onder controle krijgen van de ziekte. Het dieet is vooral onderzocht bij kinderen met de ziekte van Crohn die een opvlamming van de ziekte hadden. In 2017 heeft Sigall Boneh het CDED dieet, in combinatie met gedeeltelijke drinkvoeding, bestudeerd bij slechts 10 kinderen en 11 volwassenen die niet meer reageerden op medicatie (zogenoemde biologicals). De helft van de volwassen patiënten kwam in remissie na 12 weken het CDED dieet te hebben gevolgd.

In 2022 is er ook een kleine studie uitgevoerd bij volwassenen met de ziekte van Crohn die een matige ziekte activiteit hadden en niet meer reageerden op biologicals. Deze studie heeft aangetoond dat het CDED dieet, al dan niet aangevuld met drinkvoeding, deze groep volwassenen in remissie kan brengen[2]. Momenteel werken verschillende landen samen om met een groter onderzoek, de Paradise Trial, deze eerdere resultaten van het positieve effect van het CDED dieet te bevestigen.

Wat is het CDED dieet?

Het CDED dieet bestaat uit 3 fasen. In fase 1 wordt 50% van de benodigde energie gehaald uit drinkvoeding, en mag je verder niets eten, behalve een aantal verplichte voedingsmiddelen. Je moet dagelijks 2 bananen, 1 appel, 2 eieren, een stuk kipfilet en 2 aardappelen eten. Deze fase duurt 6 weken. In fase 2 gebruik je nog 25% van je eten als drinkvoeding en mag je eten van een iets uitgebreidere lijst met producten. Ook deze fase duurt 6 weken. Vervolgens mag je in fase 3 (de onderhoudsfase) weer meer producten eten en gebruik je daarnaast nog steeds 25% van je energie als drinkvoeding.

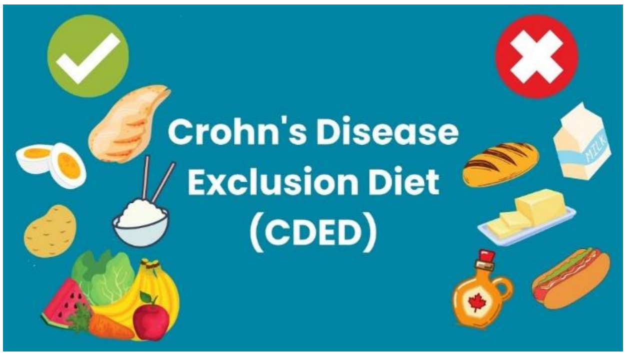

Omdat dit een dieet veel voedingsmiddelen vermijdt en strikt gevolgd moet worden, wordt dit alleen uitgevoerd onder begeleiding van een diëtist. Wil je weten of dit onderdeel van jouw behandeling kan zijn, kun je dit bespreken met je behandelend arts. Op dit moment wordt het nog niet als standaard behandeling ingezet omdat het nog in de laatste onderzoeksfase is.

Referenties

1. Sigall Boneh, R., Sarbagili Shabat, C., Yanai, H., Chermesh, I., Ben Avraham, S., Boaz, M., & Levine, A. (2017). Dietary therapy with the Crohn's disease exclusion diet is a successful strategy for induction of remission in children and adults failing biological therapy. Journal of Crohn's and Colitis, 11(10), 1205-1212.

2. Yanai H, Levine A, Hirsch A, Boneh RS, Kopylov U, Eran HB, Cohen NA, Ron Y, Goren I, Leibovitzh H, Wardi J, Zittan E, Ziv-Baran T, Abramas L, Fliss-Isakov N, Raykhel B, Gik TP, Dotan I, Maharshak N. The Crohn's disease exclusion diet for induction and maintenance of remission in adults with mild-to-moderate Crohn's disease (CDED-AD): an open-label, pilot, randomised trial. Lancet Gastroenterol Hepatol. 2022 Jan;7(1):49-59. doi: 10.1016/S2468-1253(21)00299-5. Epub 2021 Nov 2. PMID: 34739863.

Waar moet ik bij mijn voeding op letten als ik op ga?

Ziek worden is voor niemand fijn. Zeker niet als je op vakantie bent. Toch krijgen veel Nederlanders een voedselinfectie of voedselvergiftiging tijdens hun vakantie. Dit leidt soms tot overgeven, diarree en soms zelf uitdroging. Daarom hier een paar tips om veilig te eten en drinken op vakantie.

1. Is het kraanwater drinkbaar? **In veel landen kun je het kraanwater beter niet drinken**. Controleer dit voor vertrek. Als het kraanwater niet veilig is om te drinken, gebruik dan flessenwater om te drinken en je tanden mee te poetsen. Je kunt dit ook gebruiken om groente en fruit te wassen of je keukengerei mee schoon te spoelen. Vermijd drankjes met ijsblokjes, omdat deze vaak van kraanwater worden gemaakt.

2. **Wees voorzichtig met bederfelijke producten**. Bij warm weer kunnen bacteriën zich snel vermenigvuldigen. Daarom is het belangrijk om vlees, vis en gesneden groenten zo koel mogelijk te bewaren. Zet je koelkast op 4 graden Celsius.

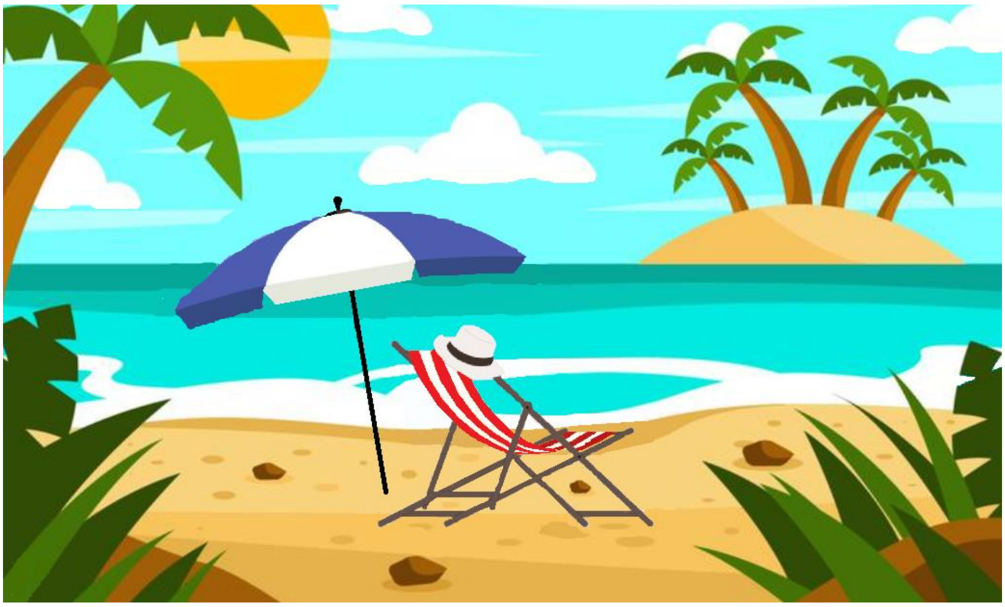

3. **Let ook op de hygiëne in het buitenland**. Worden bederfelijke producten zoals vlees, vis, salades en rauwkost goed gekoeld bewaard? Controleer of verhitte producten echt goed gaar zijn en niet aan de buitenkant verbrand, maar van binnen nog rauw. Het is het beste om voedsel te eten waarvan je de bereiding hebt gezien. Restaurants waar veel lokale bewoners met kinderen eten, hebben doorgaans een lagere kans dat voedsel lang blijft liggen. Vermijd risicovolle rauwe producten. Probeer zo min mogelijk rauwe producten te eten, zoals (half) rauw vlees (tartaar), rauwe vis (sushi), rauwe schaal- of schelpdieren, rauwe melk of rauwe kaas, en rauwe kiemgroenten (zoals taugé).

4. **Veilig koken op vakantie.** Zorg er ook op vakantie voor dat je keukengerei schoon is en dat je hygiënisch te werk gaat. Was altijd je handen met zeep voordat je voedsel gaat bereiden, vooral na het aanraken van rauw vlees en rauwe groenten, en zeker na een toiletbezoek. Was fruit en groenten grondig voordat je ze eet. Gebruik hiervoor flessenwater (of gekookt kraanwater) als het kraanwater niet veilig is om te drinken. Schil fruit indien mogelijk. Reinig keukengerei dat in contact is geweest met rauwe producten in heet water met afwasmiddel. Gebruik een schone theedoek of laat je keukengerei aan de lucht drogen.

Wanneer raadpleeg ik een diëtist?

Voor mensen met IBD kan het goed zijn om een diëtist te raadplegen. Dit is verstandig als je **veel dingen niet durft te eten**, ook na de adviezen die we je gegeven hebben. Een diëtist kijkt dan samen met je welke dingen je kunt eten, zodat je toch voldoende voedingsstoffen binnen krijgt.

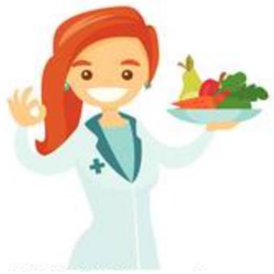

Een diëtist kan maatwerk leveren in adviezen voor je voeding. Dit is extra belangrijk als je een **verminderde eetlust** hebt. Dit kan optreden wanneer de ziekte weer actief is, maar ook als de ziekte rustiger is.

Een diëtist kan je ook adviezen geven voor het eten **na een periode met sondevoeding**.

Ook wanneer je een **te laag gewicht** voor je lengte hebt of ondervoed dreigt te raken is het verstandig om een diëtist in te schakelen.

Een diëtist kan ook samen met je kijken wat je kunt eten om toch voldoende binnen te krijgen en in een zo goed mogelijke conditie te komen of blijven.

Waar vind ik een diëtist die meer weet van voeding bij IBD?

In 2023 hebben we in Nederland zoveel mogelijk diëtisten kennisnetwerken opgezet met diëtisten die extra interesse hebben in IBD. Deze hebben ook allemaal een extra ‘Voeding bij IBD’ scholing gevolgd. Je vindt deze diëtisten op de lijst via de link hieronder.

Totale lijst diëtisten geschoold in IBD juli 2024 voor patiënten
