# Supplementary material for: Dietary Analyses and Personalized Advice in Patients with Inflammatory Bowel Disease: The DAPA Study
Source: Nutrients. 2026 Aug 11;18(16):2626. doi: 10.3390/nu18162626 (PMC13516606; doi:10.3390/nu18162626)
Supplement: Supplementary file 1 [file nutrients-18-02626-s001.zip › nutrients-4420788-supplement file/Supplementary File S2. Example of a personalized GINQ-FFQ feedback report (Dutch version)..pdf]

Energie (calorieën)
Je lichaam heeft energie nodig om te kunnen functioneren. Dit wordt uitgedrukt in kilocalorieën of kilojoules. 1 kilocalorie = 4,2 kilojoules. Meestal wordt gesproken over calorieën.

Hoeveel calorieën het lichaam nodig heeft, verschilt voor mannen en vrouwen, per leeftijd en of er sprake is van een actieve of inactieve leefstijl.
Het aantal grammen vetten, koolhydraten, eiwitten en alcohol dat een voedingsmiddel bevat, bepaalt hoeveel energie een voedingsmiddel bevat.

- 1 gram vet levert 9 kcal
- 1 gram eiwit levert 4 kcal
- 1 gram koolhydraten levert 4 kcal
- 1 gram alcohol levert 7 kcal

U gebruikt volgens de vragenlijst gemiddeld 2948 kilocalorieën.

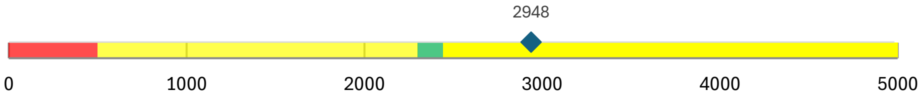

Je voeding bevat een te ruime hoeveelheid energie voor je geslacht, leeftijd en activiteiten. Houd je gewicht in de gaten en probeer vooral de ongezondere producten te vermijden.

Eiwit
Eiwit ofwel proteïne is een voedingsstof en een bouwstof. Eiwit levert calorieën (energie) en aminozuren. Deze aminozuren zijn de bouwstenen voor de eiwitten in ons lichaam. Dierlijke eiwitten zitten vooral in vlees, vis, melk, kaas en eieren. Plantaardige eiwitten zitten vooral in brood, graanproducten, peulvruchten en noten. De eiwitten van dierlijke oorsprong lijken meer op die van de mens en daar kunnen we meer spieren uit maken. Bij de eiwitten van plantaardige oorsprong is de verhouding van de aminozuren vaak wat anders waardoor we er minder lichaamseiwit uit kunnen maken. Door bijvoorbeeld peulvruchten, zoals erwten en linzen te combineren met graanproducten kan er toch weer een goede verhouding ontstaan waardoor ze samen toch weer goede eiwitten leveren. Het eiwit van de sojaboon (ook in tofu en tempeh) is wel van een goede kwaliteit.
Volwassenen hebben gemiddeld 0,83 gram eiwit per kg lichaamsgewicht nodig

Je eet gemiddeld 142,5 gram eiwit per dag, waarvan 59,3 gram plantaardig en 83,2 gram dierlijk.
Met jouw lichaamsgewicht van 66,7 kg is je gemiddelde eiwit-inname 2,14 gram per kilogram lichaamsgewicht

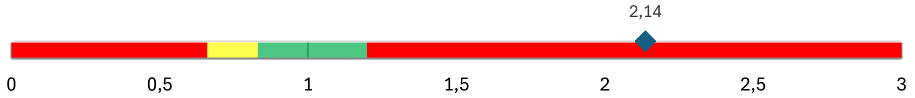

Je voeding bevat te veel eiwit. Dit is niet nodig. Probeer de hoeveelheid eiwitrijke producten te beperken

Vet
Vet is een bron van energie en bevat verzadigde en onverzadigde vetzuren. Daarnaast bevatten de vetten vitamine A, vitamine D, vitamine E en essentiële vetzuren. Belangrijke onverzadigde vetzuren zijn omega-3 vetzuren.

Je eet gemiddeld 121,9 gram vet per dag.
De bijdrage van vet aan jouw gemiddelde energie-inname is 37%.

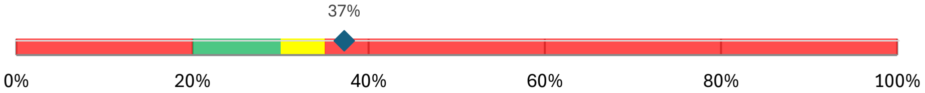

Je voeding bevat te veel vet. Hierdoor loop je de kans dat je zwaarder wordt en verhoog je je risico op hart- en vaatziekten, diabetes en darmkanker. Beperk de voedingsmiddelen die veel vetten bevatten. Kies vaker voor halfvolle of magere producten.

Omega 3 vetzuren
De omega 3-vetzuren DHA en EPA komen voor in vis en verlagen de kans op hart- en vaatziekten. Ze spelen een belangrijke rol bij de hersenontwikkeling en het gezichtsvermogen van ongeboren baby's.

Uw gemiddelde inname van omega 3-vetzuren is berekend op 5,49 gram

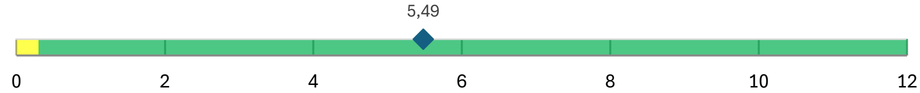

Je eet een prima hoeveelheid omega 3 vetzuren

Verzadigd vet

Verzadigd vet verhoogt het LDL-cholesterol van het bloed. Een te hoog LDL-cholesterol is niet goed voor hart- en bloedvaten. Door producten met veel verzadigd vet te vervangen door producten met veel onverzadigd vet, kun je de kans op hart- en vaatziekten verlagen.
De hoeveelheid verzadigd vet verschilt sterk per product. Verzadigd vet komt vooral veel voor in dierlijke producten, zoals vet vlees, volle melkproducten, roomboter en volvette kaas (48+). Ook in sommige plantaardige producten zoals kokosvet en palmolie zit veel verzadigd vet. Omdat deze vetten veelal in koek, gebak en snacks gebruikt worden, zit ook in die producten vaak veel verzadigd vet.
Meestal geldt dat hoe zachter of vloeibaarder het vet, hoe minder verzadigde vetten erin zitten. Denk aan halvarine in kuipjes, olie en vloeibaar bak- en braadvet in knijpflessen. Vetten die hard zijn bij kamertemperatuur, bijvoorbeeld roomboter of harde margarine in een wikkel, bevatten vooral verzadigd vet.

Je eet gemiddeld 32,5 gram verzadigd vet per dag.

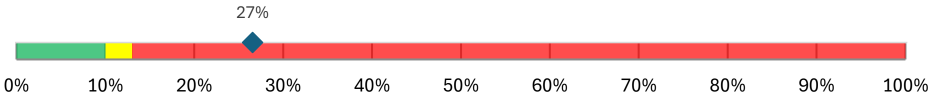

Je voeding bevat te veel verzadigd vet. Dit verhoogt de kans op hart- en vaatziekten. Probeer de hoeveelheid verzadigd vet te verminderen. Verzadigd vet zit veel in dierlijke producten, zoals vet vlees en volle melkproducten en volvette kaas. Ook zit er veel verzadigd vet in koek, gebak en snacks.

Koolhydraten

Suikers, zetmeel en vezels vormen samen de koolhydraten in onze voeding. Suikers en zetmeel leveren vooral energie voor het lichaam. Koolhydraten in de vorm van zetmeel, die ook veel vezels en vitaminen bevatten hebben met name gunstige effecten op onze gezondheid. Dit zijn vooral volkoren graanproducten zoals volkorenbrood, volkorenpasta, aardappelen, peulvruchten, groente en fruit. Suikers die geen vezels en vitamines bevatten hebben een negatieve invloed op onze gezondheid. Deze zitten vooral in frisdranken, koek en snoep.

Je eet gemiddeld 288 gram koolhydraten per dag. Dit is 39% van je energie-inname.
Hiervan eet je gemiddeld 102 gram suiker. Dit is 14% van je energie-inname
Aanbevolen wordt om maximaal 10% van je energie uit suikers te halen.
Voor u is 10% energie uit suikers niet meer dan 74 gram per dag.

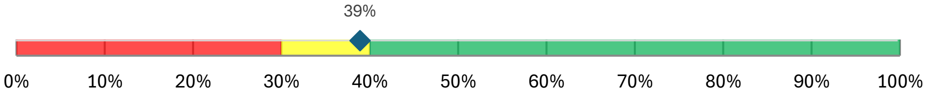

De hoeveelheid koolhydraten in je voeding is aan de krappe kant. Probeer meer producten te eten met gezonde koolhydraten zoals volkoren graanproducten zoals volkorenbrood, volkorenpasta, aardappelen, peulvruchten, groente en fruit. Het kan ook zijn dat u relatief te veel vet eet.

Vezels
Voedingsvezels ofwel vezels zijn belangrijk voor een goede spijsvertering, geven een verzadigd gevoel na de maaltijd en verminderen het risico of overgewicht, hart- en vaatziekten, diabetes en darmkanker.
Voedingsvezels zitten uitsluitend in plantaardige producten, zoals groente, fruit, aardappelen, volkorenbrood, ontbijtgranen, peulvruchten en noten.

Je eten bevat gemiddeld 49 gram vezels per dag.

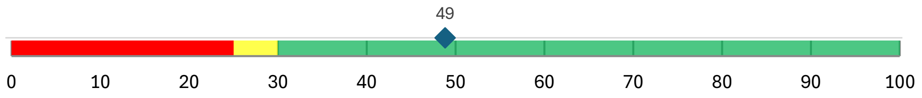

Je voeding bevat voldoende voedingsvezel. Dit is een hele gezonde basis van je voeding.

Alcohol
Het advies is om geen alcohol te drinken, of in ieder geval niet meer dan 1 glas per dag. Dit advies geldt zowel voor mannen als voor vrouwen.
Het drinken van meer dan 1 glas per dag heeft alleen maar negatieve gezondheidseffecten.
Het vergroot het risico op beroerte, borstkanker, darmkanker en longkanker.

Je drinken bevat gemiddeld 3 gram alcohol per dag.

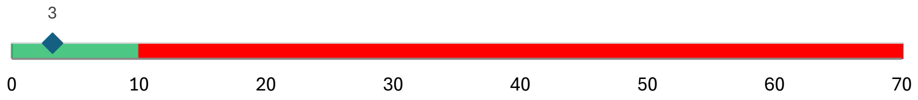

Je alcoholgebruik is prima. Meer dan 1 glas alcohol per dag heeft nadelige effecten voor je gezondheid.

Water
Water voorziet je lichaam van vocht. Omdat water geen calorieën bevat is water een goede dorstlesser .

Je gebruikt gemiddeld 2339 gram water per dag.

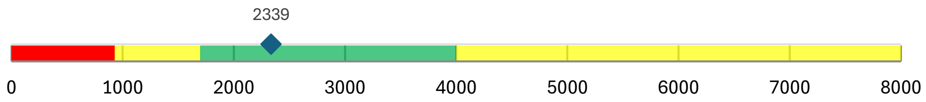

Je drinkt een prima hoeveelheid vocht!

Vitamine A
Vitamine A is een vetoplosbaar vitamine dat van belang is voor de normale groei, een gezonde huis, haar en nagels en zorgt voor een goede werking van de ogen en het afweersysteem. Vitamine A zit vooral in dierlijke producten. Het lichaam kan ook zelf vitamine A maken uit carotenoïden, die in plantaardige producten zitten.

Je gebruikt gemiddeld 1050 microgram vitamine A per dag.

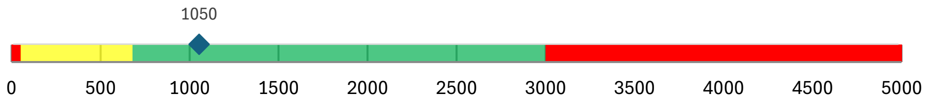

Je voeding bevat een prima hoeveelheid vitamine A.

Vitamine B1 (Thiamine)
Vitamine B1 ofwel thiamine speelt een belangrijke rol in de energievoorziening van ons lichaam en draagt bij aan een goede werking van de hartspier en het zenuwstelsel. Brood en graanproducten, aardappelen, groente, vlees en vleeswaren, melk en melkproducten zijn onze belangrijkste bronnen van vitamine B1.

Je gebruikt gemiddeld 1,6 milligram vitamine B1 per dag.

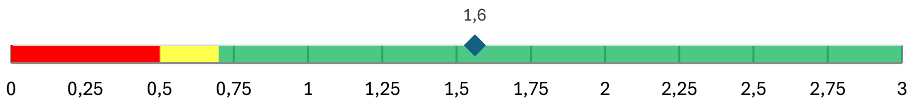

Prima, je voeding bevat voldoende vitamine B1

Vitamine B2 (Riboflavine)
Vitamine B2 ofwel riboflavine is vooral nodig voor de energievoorziening van het lichaam. Vitamine B2 zit vooral in melk en melkproducten, maar ook in vlees, vleeswaren, groente, fruit, brood en graanproducten.

Je gebruikt gemiddeld 1,8 milligram vitamine B2 per dag.

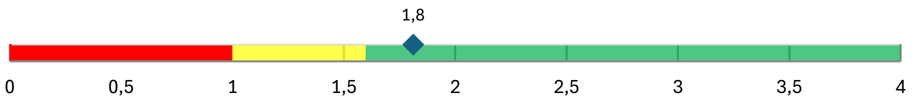

Prima, je voeding bevat voldoende vitamine B2

Vitamine B6
Vitamine B6 is belangrijk voor de afbraak en opbouw van aminozuren. Aminozuren zijn de bouwstenen van eiwitten. Vitamine B6 reguleert de werking van bepaalde hormonen en is nodig voor de groei, de bloedaanmaak en een goede werking van het immuunsysteem en het zenuwstelsel.

Je gebruikt gemiddeld 2,8 milligram vitamine B6 per dag.

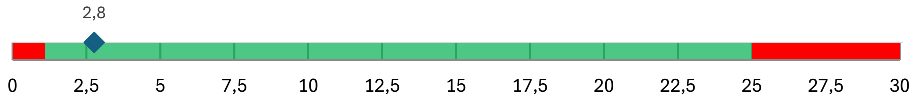

Je hoeveelheid vitamine B6 in je voeding is prima.

Foliumzuur (vitamine B11)
Foliumzuur ofwel vitamine B11 is een wateroplosbaar vitamine dat nodig is voor de aanmaak van witte en rode bloedcellen. Het is belangrijk voor de groei en voor de vroege ontwikkeling van het ongeboren kind. Bij een tekort aan foliumzuur ontstaat bloedarmoede. Bij het ongeboren kind verhoogt het de kans op een open ruggetjes, hazenlip en gespleten verhemelte. Om deze kans te verkleinen wordt vrouwen die zwanger willen worden of zwanger zijn aangeraden dagelijks 400 microgram foliumzuur te slikken totdat ze 10 weken zwanger zijn.
Foliumzuur komt voor in (groene) groenten, volkoren producten, brood, vlees en zuivel.

Je gebruikt gemiddeld 493 microgram foliumzuur (vitamine B11) per dag.

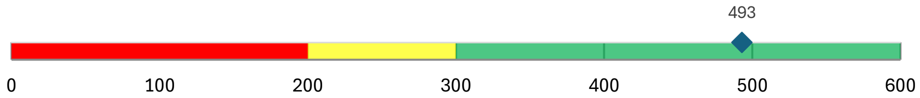

Prima! Je voeding bevat voldoende foliumzuur.

Vitamine B12 (cobalamine)
Vitamine B12 of cobalamine is een wateroplosbaar vitamine dat nodig is voor de aanmaak van rode bloedcellen. Deze rode bloedcellen zijn nodig voor het zuurstoftransport in je bloed. Daarnaast is vitamine B12 nodig voor een goede werking van het zenuwstelsel.
Vitamine B12 zit alleen in dierlijke producten, zoals melk, melkproducten, vlees, vleeswaren, vis en eieren. Daarom wordt veganisten aangeraden om een vitamine B12 supplement te gebruiken of producten met toegevoegd vitamine B12 te gebruiken.

Je gebruikt gemiddeld 7,8 microgram vitamine B12 per dag.

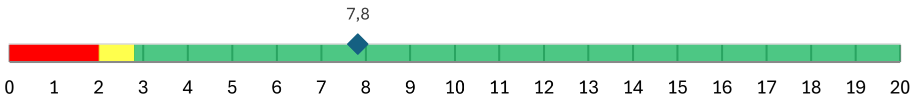

Prima. Je voeding bevat voldoende vitamine B12.

Vitamine C (ascorbinezuur)
Vitamine C ofwel ascorbinezuur is een bekend wateroplosbaar vitamine, dat werkt als een antioxidant, nodig is bij de vorming van bindweefsel, zorgt voor de opname van ijzer en zorgt voor een betere weerstand.
Vitamine C zit in fruit (vooral citrusfruit, kiwi, bessen en aardbeien), groente (vooral koolsoorten en paprika) en aardappelen.

Je gebruikt gemiddeld 223 miilligram vitamine C per dag.

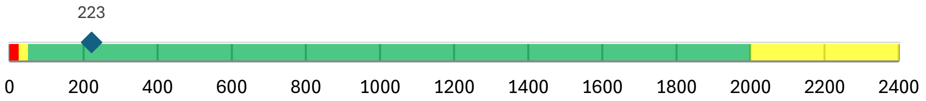

Prima. Je voeding bevat voldoende vitamine C.

Vitamine D (15 microgram /dag) [VITD]
Vitamine D zorgt ervoor dat calcium uit de voeding in het lichaam opgenomen wordt. Het is dus belangrijk voor de groei en opbouw van stevige botten en tanden. Ook speelt vitamine D een belangrijke rol bij de werking van onze spieren en het immuunsysteem.
Ons lichaam kan zelf vitamine D maken in onze huid onder invloed van zonlicht. Daarnaast zit vitamine D vooral in vette vis, zoals haring, paling, zalm en makreel. Verder zit er nog vitamine D in vlees en eieren en wordt het toegevoegd aan halvarine, margarine en bak- en braadproducten.

Je gebruikt gemiddeld 3,7 microgram vitamine D per dag.

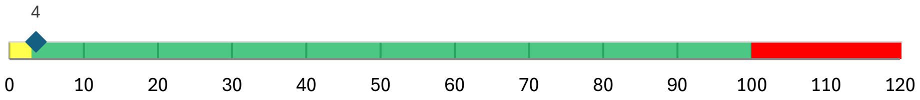

Prima! De hoeveelheid vitamine D in je voeding lijkt voldoende.

Vitamine E [VITE]
Er is onvoldoende onderbouwing voor goede aanbevolen hoeveelheden. In Nederland komen normaal gesproken geen tekorten aan vitamine E voor. Vitamine E zit vooral in olie, margarine, halvarine, volkoren en bruinbrood, noten, groente en fruit.
Bij alle waarden aangeven wat de inname is Er is onvoldoende onderbouwing voor goede aanbevolen hoeveelheden. In Nederland komen normaal gesproken geen tekorten aan vitamine E voor. Vitamine E zit vooral in olie, margarine, halvarine, volkoren en bruinbrood, noten, groente en fruit.

Je gebruikt gemiddeld 18,3 microgram vitamine E per dag.

Natrium
Zout is natriumchloride. Natrium is belangrijk voor het regelen van de vochtbalans en de bloeddruk. Te veel zout ofwel natrium kan leiden tot een hoge bloeddruk. Een hoge bloeddruk verhoogt de kans op hart- en vaatziekten. Het komt zelden voor dat mensen te weinig zout binnen krijgen

Je gebruikt gemiddeld 2953 milligram natrium per dag.

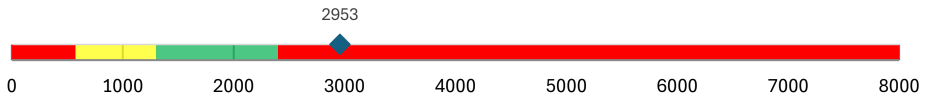

Je voeding bevat te veel zout. Dit kan leiden tot een hoge bloeddruk, dat weer een grotere kans geeft op hart- en vaatziekten. Probeer wat minder producten te eten die veel (keuken)zout bevatten, zoals kant- en klaar producten, soep, sauzen, snacks, bewerkt vlees, zuurkool, pizza, haring, koek en gebak.

Kalium
Kalium is een mineraal dat samen met chloride en natrium betrokken is bij het regelen van de vochtbalans en bloeddruk van je lichaam. Kalium werkt het bloeddrukverhogende effect van natrium tegen.

Je gebruikt gemiddeld 3600 milligram kalium per dag.

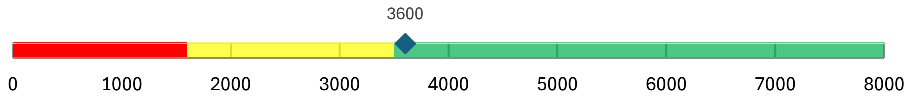

Prima, je voeding bevat voldoende kalium.

Calcium
Calcium is een mineraal dat je lichaam nodig heeft voor de opbouw en onderhoud van je botten en gebit. Voldoende calcium helpt botontkalking op latere leeftijd te voorkomen.

Je gebruikt gemiddeld 1160 milligram calcium per dag.

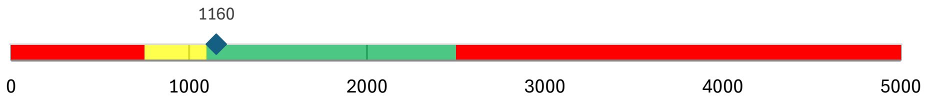

Prima! Je voeding bevat een goede hoeveelheid calcium

Magnesium
Magnesium is een mineraal dat je lichaam nodig heeft voor de vorming van bot en spieren. Het speelt een rol bij de werking van spieren en de overdracht van zenuwprikkels

Je gebruikt gemiddeld 589 milligram magnesium per dag.

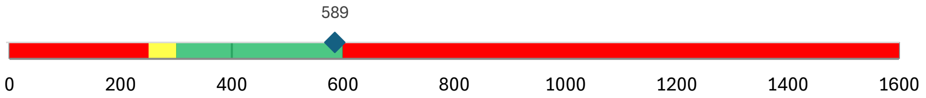

Je voeding bevat een prima hoeveelheid magnesium.

IJzer
IJzer is een mineraal dat belangrijk is voor de vorming van het hemoglobine in onze rode bloedcellen. Deze cellen vervoeren het zuurstof door ons lichaam. IJzer komt in 2 vormen voor: het heemijzer en het non-heemijzer. Het heemijzer zit in dierlijke producten en wordt beter opgenomen dan het non-heemijzer dat in plantaardige producten zit. IJzertekort is te herkennen aan vermoeidheid, bleke huid, rusteloze benen en snel buiten adem zijn.

Je gebruikt gemiddeld 19,2 milligram ijzer per dag.

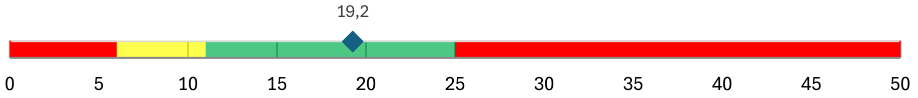

Je voeding bevat een prima hoeveelheid ijzer.

Selenium
Selenium is een sporenelement dat als antioxidant functioneert. Selenium zit veel in dierlijke producten (vis en vlees) maar ook plantaardige voedingsmiddelen.

Je gebruikt gemiddeld 109 microgram selenium per dag.

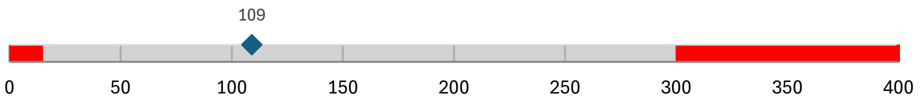

Selenium komt erg verspreid in de voeding voor. Er is geen duidelijke streefwaarde voor voldoende inname van selenium.

Zink
Zink is een sporenelement dat een belangrijk onderdeel is van veel enzymen die in het lichaam de stofwisseling regelen. Zink is onder andere nodig bij de opbouw van eiwitten, de groei en ontwikkeling van weefsel en bij een goede werking van het afweer- ofwel immuunsysteem

Je gebruikt gemiddeld 16,9 milligram zink per dag.

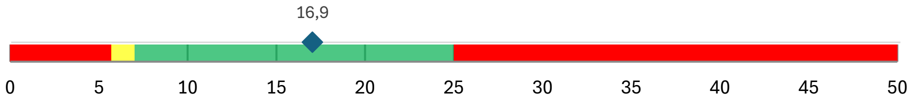

Je voeding bevat een prima hoeveelheid zink

Jodium [ID]
Jodium is een sporenelement dat betrokken is bij de productie van schildklierhormonen. Schildklierhormonen zijn betrokken bij de stofwisseling, goede groei en ontwikkeling van het zenuwstelsel. Door een jodium tekort gaat de schildklier trager werken en opzwellen. Jodium zit in zeevis, eieren, zuivelproducten en zeewier. Toegevoegd jodium zit in vleeswaren, gejodeerd zout (25 mg per kg) en in hogere concentraties in gejodeerd bakkerszout (65 mg per kg).

Je gebruikt gemiddeld 294 milligram jodium per dag.

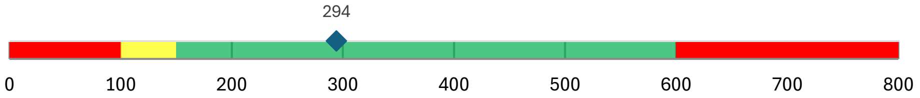

Prima. Je voeding bevat voldoende jodium.

Fosfor [P]
Er is onvoldoende onderbouwing voor goede aanbevolen hoeveelheden. In Nederland komen normaal gesproken geen tekorten voor. Fosfor zit vooral in eiwitrijke producten zoals melk, kaas, vis, vlees, peulvruchten en volkorenproducten.

Je gebruikt gemiddeld 2431 milligram fosfor per dag.
